# NMR-Based Metabolomics Analysis Predicts Response to Neoadjuvant Chemotherapy for Triple-Negative Breast Cancer

**DOI:** 10.3389/fmolb.2021.708052

**Published:** 2021-11-02

**Authors:** Xiangming He, Jinping Gu, Dehong Zou, Hongjian Yang, Yongfang Zhang, Yuqing Ding, Lisong Teng

**Affiliations:** ^1^ The First Affiliated Hospital, Zhejiang University School of Medicine (FAHZU), Hangzhou, China; ^2^ Chinese Academy of Sciences, Institute of Basic Medicine and Cancer (IBMC), The Cancer Hospital of the University of Chinese Academy of Sciences (Zhejiang Cancer Hospital), Hangzhou, China; ^3^ Key Laboratory for Green Pharmaceutical Technologies and Related Equipment of Ministry of Education, College of Pharmaceutical Sciences, Zhejiang University of Technology, Hangzhou, China

**Keywords:** metabolomics, triple-negative breast cancer, metabolic pathway, ROC curve, NMR spectroscopy

## Abstract

Triple-negative breast cancer (TNBC) is the most fatal type of breast cancer (BC). Due to the lack of relevant targeted drug therapy, in addition to surgery, chemotherapy is still the most common treatment option for TNBC. TNBC is heterogeneous, and different patients have an unusual sensitivity to chemotherapy. Only part of the patients will benefit from chemotherapy, so neoadjuvant chemotherapy (NAC) is controversial in the treatment of TNBC. Here, we performed an NMR spectroscopy–based metabolomics study to analyze the relationship between the patients’ metabolic phenotypes and chemotherapy sensitivity in the serum samples. Metabolic phenotypes from patients with pathological partial response, pathological complete response, and pathological stable disease (pPR, pCR, and pSD) could be distinguished. Furthermore, we conducted metabolic pathway analysis based on identified significant metabolites and revealed significantly disturbed metabolic pathways closely associated with three groups of TNBC patients. We evaluated the discriminative ability of metabolites related to significantly disturbed metabolic pathways by using the multi-receiver–operating characteristic (ROC) curve analysis. Three significantly disturbed metabolic pathways of glycine, serine, and threonine metabolism, valine, leucine, and isoleucine biosynthesis, and alanine, aspartate, and glutamate metabolism could be used as potential predictive models to distinguish three types of TNBC patients. These results indicate that a metabolic phenotype could be used to predict whether a patient is suitable for NAC. Metabolomics research could provide data in support of metabolic phenotypes for personalized treatment of TNBC.

## Introduction

According to the American Cancer Society estimates, in the female patients, breast cancer (BC) was the tumor with the highest incidence (about 30%) among the new invasive cancer cases in the US in 2020; in addition, BC had the second highest mortality rate, accounting for 15% among the new cancer death cases (Siegel et al., 2020). Approximately 10–20% of all invasive BC cases were triple-negative breast cancer (TNBC) (Kumar and Aggarwal, 2016). Due to the lack of estrogen receptor (ER), progesterone receptor (PR), and human epidermal growth factor receptor 2 (HER2) expression, TNBC lacked standardized treatment strategies (Yin et al., 2020). Chemotherapy is still the most common treatment option for TNBC (Masuda et al., 2017). Neoadjuvant chemotherapy (NAC) is controversial in the treatment of TNBC. A part of the TNBC patients were sensitive to chemotherapy drugs, and about 30–40% of patients’ pathological symptoms disappeared completely. This was defined as pathological complete response (pCR) (Liedtke et al., 2008; Gluck et al., 2012). Patients whose pathological symptoms have not changed at all were defined to have pathological stable disease (pSD). Some patients’ pathological symptoms were somewhere in between, and this was defined as pathological partial response (pPR). On the contrary, the cancer recurrence rate and metastasis rate of patients with residual disease after NAC have greatly increased (Liedtke et al., 2008; Masuda et al., 2013). After NAC differences in clinical response and survival tips, it is necessary to consider a more detailed classification in clinical TNBC. With the rise of metabolomics research, differences in metabolic phenotypes could provide us with a new idea of NAC for TNBC.

Metabolomics is the study of the multi-parametric metabolic response of living systems to pathophysiological stimuli or genetic modification (Nicholson et al., 1999). Metabolomics is a part of systems biology, which is downstream concerning the other -omic sciences (Vignoli et al., 2019). Metabolomics has a wide range of applications, including human health and diseases (Johnson et al., 2016), animals (Kirwan 2013), plants (Pontes et al., 2017), microorganisms (Ramirez-Gaona et al., 2017), and other areas (Kim H.-Y. et al., 2016; Munger et al., 2017). More and more researchers were using metabolomics technology to study tumor metabolism (Armitage and Ciborowski, 2017; Kumar and Misra, 2019). In the BC field, metabolomics has been fully applied (McCartney et al., 2018). Four metabolites of glutamine, isoleucine, threonine, and linolenic acid could be used as potential markers for predicting response to NAC for BC, by comprehensive use of nuclear magnetic resonance (NMR) spectroscopy and mass spectrometry (MS) techniques (Wei et al., 2013). However, studies on the prognosis of TNBC surgery have not been performed.

On the contrary, metabolomics was also applied to individualize treatment (Jacob et al., 2019). Our early study used NMR-based metabolomics for finding the new biomarkers of colorectal cancer (Gu et al., 2019a). Mohammad *et al.* reviewed the application of metabolomics in the prognosis of acute coronary syndrome (Pouralijan Amiri et al., 2019). Similarly, metabolomics was also applied to bariatric surgery (Samczuk et al., 2018). In this study, we used metabolomics for the evaluation of NAC for TNBC. Our work looks forward to discovering metabolic phenotypes and differential metabolic pathways between the patients with pCR, pPR, and pSD.

## Materials and Methods

### Chemical Reagents

Deuterated reagents of D_2_O and sodium 3-(trimethylsilyl)propionate-2,2,3,3-d4 (DSS) were purchased from Cambridge Isotope Laboratories, Inc. (Andover, MA, United States). Chromatographic grade methanol was bought from Sigma-Aldrich (St. Louis, MO, United States). Other analytical grade reagents (NaH_2_PO_4_·2H_2_O and K_2_HPO_4_·3H_2_O) were purchased from J&K Scientific Ltd. (Beijing, China). All ultra-pure water used in this study was produced by a Milli-Q IQ 7000 system.

### Selection of TNBC Patients and Collection of Serum Samples

TNBC patients were recruited and treated at the Department of Breast Surgery, Zhejiang Cancer Hospital (tumor hospital affiliated to the University of Chinese Academy of Sciences). These female patients were enrolled in the study between 2019 and 2020. This study was performed in accordance with protocols approved by the Zhejiang Cancer Hospital Ethics Committee. The clinicopathological characteristics of participating subjects are summarized in Supplementary Table S1. There were 52 patients in our study, of which 8 had pCR, 16 had pSD, and 28 had pPR. There was no difference in age and BMI index of these patients. Based on T category, the classification of patients was mainly concentrated in III and IV stages. The criteria for patient selection included 1) pathologically confirmed primary TNBC; 2) being in line with NAC indications; 3) age of 20–65 years; and 4) performance status (PS) score 0–1. The criteria for patient exclusion included 1) non-primary TNBC; 2) combination with other malignant tumors; 3) not meeting NAC indications; 4) combination with blood system diseases and kidney diseases, including hemophilia, aplastic anemia and myelodysplastic syndromes, immune thrombocytopenia, sickle cell disease, sickle cell trait, and other hemoglobinopathies, diabetes, and thalassemias; 5) patients with advanced BC; 6) age >65°years or <20°years; and 7) those who cannot tolerate chemotherapy and surgery, or those who have a PS score >1.

The effect of NAC in the treatment of TNBC was comprehensively obtained by magnetic resonance imaging (MRI) and two-dimensional or three-dimensional ultrasound and mammography with histopathology. According to these test results, patients were divided into three groups, including pCR, pPR, and pSD. Here, pCR indicates all tumor tissue is disappeared, pPR indicates tumor volume is reduced by more than 30%, while the tumor volume is reduced by less than 30% or increased by not more than 20% in pSD (Neubauer et al., 2008).

Each patient had a light diet for 48 h before blood collection. After blood collection (5 ml), it was coagulated and centrifuged (4°C, 4,000 rcf, 15 min) to obtain serum. All serum samples were frozen and stored in the −80 C refrigerator until the NMR experiment.

### Pretreatment of Serum Samples and Acquisition of NMR Spectra

Before NMR data acquisition, the serum samples were thawed on ice. 300 μL serum was mixed with 600 μL methanol (Tiziani et al., 2008). Then, the mixed samples were stored in the −20 C refrigerator for 30 min. The macromolecules in sera underwent denaturation and precipitation and were removed by centrifugation (12,000 g, 4°C, 30 min). Then, all these supernatant solvents were removed by the lyophilizer. The lyophilized metabolites were redissolved in 450 μL of ultrapure water, and then 50 μL of phosphate buffer (1.5 M K_2_HPO_4_/NaH_2_PO_4_, pH 7.4, D_2_O) was added for stabilizing the pH of serum and deuterium lock-in NMR measurements. All samples were analyzed in the BRUKER AVANCE III HD 600 MHz spectrometer (BRUKER BioSpin, Germany). The one-dimensional 1H spectra were operated in the TXI probe at 300 K by using a pulse sequence with water suppression (NOESYPR1D [RD-90°-t_1_-90°-τ_m_-90°-ACQ]) with 3s relaxation delay and 120 m mixing time. The detailed acquisition parameters were described in the following kinds of literature (Gu et al., 2019b; Rohnisch et al., 2018; Shao et al., 2014). Then, the metabolites were identified from the NMR spectra according to the following reference (Rohnisch et al., 2018) and the HMDB (http://www.hmdb.ca/) (Wishart et al., 2018). Meanwhile, the two-dimensional (2D) NMR spectrum named “13C-^1^H HSQC” (heteronuclear single-quantum coherence spectroscopy) was used for the identification of metabolites (Bingo et al., 2016).

### Multivariate Statistics

Data preprocessing including data organization, removal of undesired areas, and binning was performed with MATLAB 2015b (MathWorks, Inc., United States). Minor adjustments in peak alignment between different samples were performed using the *icoshift* algorithm in MATLAB 2015b (Savorani et al., 2010). At the same time, visualization of the data was also carried out in MATLAB. According to the identified metabolites, we developed and utilized a metabolite database in this study for metabolite quantification. Using the same method of metabolite quantification from the literature of Cuperlovic-Culf et al. (2012), the relative concentrations of identified metabolites were calculated, which were based on multivariable linear regression of spectra with properly aligned metabolite data. On the contrary, the calculation of the relative concentration of the identified metabolites is also referred to as the AQuA (Rohnisch et al., 2018). Before multivariate statistical analysis, all data are normalized and par scaled. Then, principal component analysis (PCA) was performed to show clusters among all samples (Trygg et al., 2007). The partial least squares-discrimination analysis (PLS-DA) was applied for distinguishing the metabolic phenotypes among three groups (Trygg et al., 2007), and the corresponding response permutation test (RPT) was used for verifying the robustness of PLS-DA models (Lin et al., 2019). The orthogonal PLS-DA (OPLS-DA) was applied for differential metabolite analysis by using the variable importance in projection (VIP) (Cloarec et al., 2005) and the correlation coefficients (r) for the variables that are related to the first predictive component (tp1) (Cho et al., 2008). Besides, probability *p* values of the Kruskal–Wallis test and fold changes were also calculated between the pSD group, the pPR group, and the pCR group for assessing the statistical significance of differential metabolites. These four parameters (VIP value, correlation coefficients (r), *p* value, and fold change) were employed in the enhanced volcano plots for visualizing the differential metabolites (Hur et al., 2013; Lin et al., 2019).

### Identifying the Disturbed Metabolic Pathways

Metabolic pathway analysis was performed to identify significantly disturbed pathways associated with the three groups of TNBC patients in the Pathway Analysis module of MetaboAnalyst 5.0 (www.metaboanalyst.ca/) according to the relative concentration of the metabolites. Two parameters, statistical *p* value and pathway impact value, were used to evaluate the importance of the metabolic pathway. By matching the different metabolites with the metabolites in each metabolic pathway, the *p* value was calculated by the hyper-geometric test (Goeman and Bühlmann, 2007). At the same time, the pathway impact value was calculated from the topological analysis using the out-degree centrality algorithm through matched differential metabolites in metabolic pathways (Chong et al., 2018). According to the approaches described in other previous works (Gu et al., 2016; Gu et al., 2020a), we identified significantly disturbed metabolic pathways associated with *p* less than 0.05 and pathway impact values greater than 0.3.

### Analyzing the Discriminative Ability of Disturbed Metabolic Pathways

Metabolomic analysis could help develop potential biomarkers for early diagnosis in multiple medical fields (Ni et al., 2014; Nobakht 2018; Gu et al., 2019a). In this study, the multi-receiver–operating characteristic (multi-ROC) curve analysis was operated on assessing discriminant capabilities of the metabolites involved in the significantly disturbed metabolic pathways (Zweig and Campbell, 1993; Gu et al., 2020b). In multi-ROC curve analysis, the logistic regression arithmetic was used for the classification of these three groups of patients, and the area under the ROC curve (AUC) value was used for evaluating the prediction performance of the metabolites in the disturbed metabolic pathway as the AUC was greater than 0.70 (Mandrekar, 2010).

## Results

### Characteristics of Enrolled TNBC Patients

In this prospective study, detailed clinical characteristics of the participants are summarized in Supplementary Table S1. In our study, we used the TNM system to stage cancer, which is determined after cancer is assigned a letter to describe it, including tumor (T), node (N), and metastasis (M). On the contrary, a number after T (such as T1, T2, T3, or T4) might describe the tumor size. Supplementary Table S1 shows that no significant differences were observed in age, BMI, and clinical T stage, between these three groups (*p* > 0.05).

### Metabolic Profiles of Serum Samples

**FIGURE 1:**
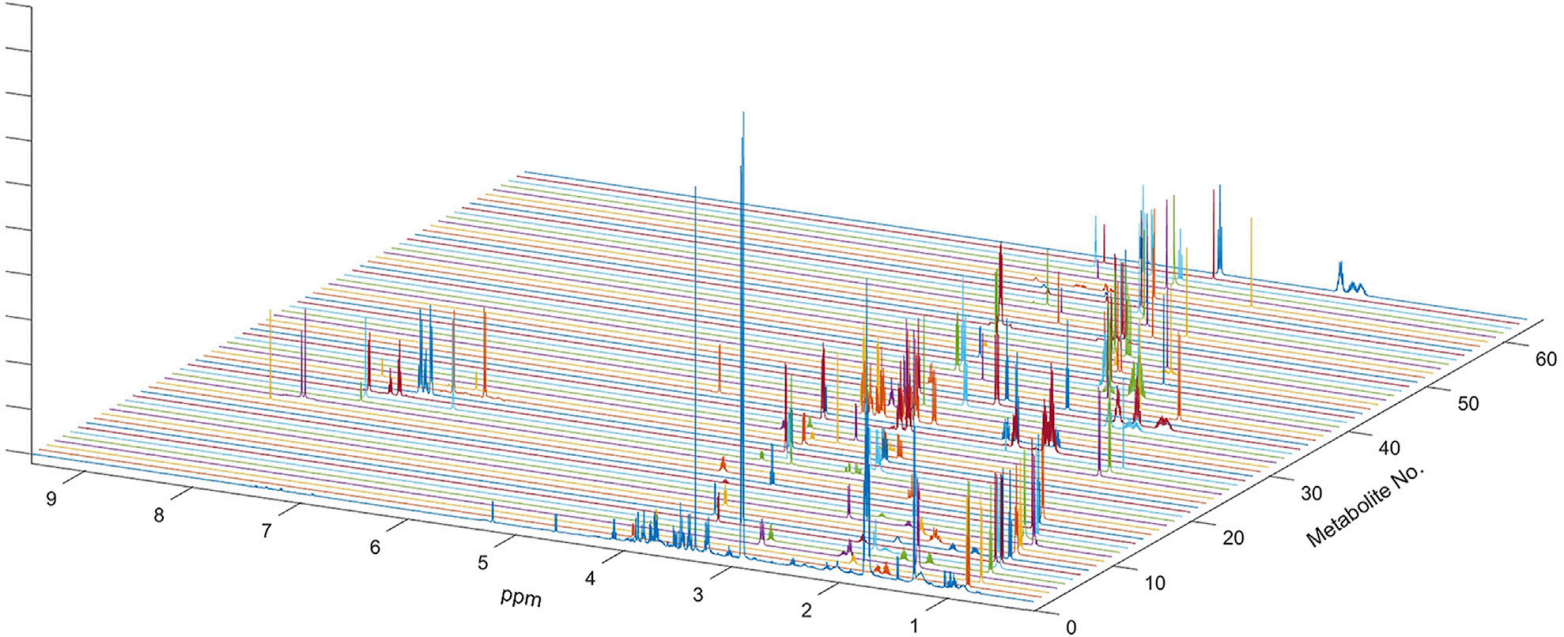
NMR spectrum of metabolites used for multi-linear regression analysis of the global spectrum. Sixty-three metabolites used in the analysis included all metabolites previously determined in the HMDB and also confirmed on the Chenomx NMR Suite. One-dimensional spectra of all sixty-three metabolites are shown along with the outline of the average spectrum for the serum sample. Complete spectra of all metabolites were used in multivariate linear regression analysis.

**FIGURE 2:**
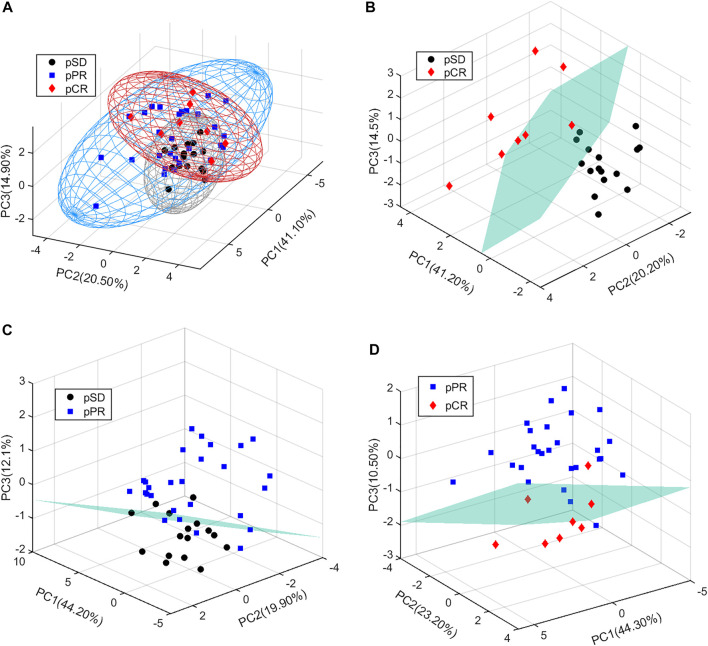
PCA score plots of the relative concentration of metabolites’ data from three groups of TNBC patients: **(A)** all patients; **(B)** pSD patients vs. pCR patients; **(C)** pSD patients vs. pPR patients; **(D)** pPR patients vs. pCR patients.

In the present study, all serum samples were collected from TNBC patients before neoadjuvant chemotherapy. A total of 63 metabolites were identified and relatively quantified from the NMR spectra (Supplementary Table S2, Figure 1, and Supplementary Figure S1), which were calculated out by using the automated method based on multivariable linear regression (Cuperlovic-Culf et al., 2012) in MATLAB (version 2015b, MathWorks, Inc., United States). Then, the multivariate statistical analysis was utilized to analyze the quantitative data of metabolites. Using the first three components, the PCA score plots are shown in Figure 2. The metabolic phenotypes of the three groups could be roughly distinguished (Figure 2A). Overall, the pCR group was distinguished from the pSD group (Figure 2B), and the pPR group was roughly distinguished from the pSD group and pCR group (Figures 2C,D).

Furthermore, supervised multivariate statistical analysis was also applied to distinguish the metabolic profiles. These PLS-DA score plots and corresponding RPTs indicated the metabolic phenotypes of the three groups could be distinguishable (Supplementary Figure S2).

### Comparison of the Relative Concentration of Different Metabolites in TNBC Patients

According to the relative quantitative value of 63 metabolites in serum samples, the mean and standard error of the mean (SEM) were calculated for each group (Table 1). Then, we performed the Kruskal–Wallis multiple-comparisons test to identify differential metabolites with *p* < 0.05 (Table 1 and Supplementary Figure S3). By comparing the serum metabolites of the three groups of patients, it was found that a total of 26 metabolites changed in the three groups. In the pCR group, there were 10 metabolites with the highest relative concentration, including τ-methylhistidine, phenylalanine, π-methylhistidine, lactic acid, glucose, alanine, glutamic acid, citric acid, dimethylamine, and phosphocholine. In the pSD group, these were seven metabolites with the highest relative concentration, including valine, 2-aminobutanoic acid, propionic acid, ethanol, proline, asparagine, and N,N-dimethylglycine. In the pPR group, there were six metabolites with the highest relative concentration, including 2-hydroxyisovaleric acid, acetoacetate, trimethylamine, creatine, myo-inositol, and ornithine, and there were five metabolites with the lowest relative concentration, including isoleucine, phenylalanine, threonine, dimethylamine, and glycerophosphocholine.

**TABLE 1:** Comparison of metabolite levels among the three groups on relative integrals calculated from 1D ^1^H-NMR spectra of TNBC patients’ serum samples.

	Mean ± SEM			Pairwise comparisons of Kruskal–Wallis test		
	pSD	pPR	pCR	pPR vs. pSD	pPR vs. pCR	pCR vs. pSD
2-Hydroxybutyric acid	1.859 ± 0.052	1.877 ± 0.059	1.827 ± 0.124	0.656	0.497	0.638
2-Hydroxyisovaleric acid	0.612 ± 0.028	0.558 ± 0.028	0.540 ± 0.045	0.012	0.573	0.019
Isocaproic acid	0.067 ± 0.014	0.063 ± 0.016	0.100 ± 0.045	0.703	0.139	0.215
3-Methyl-2-oxovaleric acid	0.690 ± 0.023	0.662 ± 0.016	0.636 ± 0.024	0.058	0.092	0.005
Isovaleric acid	0.454 ± 0.046	0.489 ± 0.042	0.385 ± 0.090	0.279	0.068	0.212
Valine	0.066 ± 0.016	0.106 ± 0.018	0.162 ± 0.038	0.002	0.024	0.001
Isoleucine	0.326 ± 0.023	0.280 ± 0.021	0.317 ± 0.055	0.006	0.247	0.794
Leucine	1.355 ± 0.032	1.421 ± 0.057	1.544 ± 0.184	0.058	0.247	0.089
2-Aminobutanoic acid	0.450 ± 0.067	0.385 ± 0.073	0.188 ± 0.072	0.204	0.001	0.000
2-Oxoisocaproate	0.264 ± 0.016	0.257 ± 0.014	0.284 ± 0.049	0.507	0.327	0.465
Isobutyric acid	0.034 ± 0.007	0.033 ± 0.006	0.025 ± 0.011	0.812	0.194	0.157
Propionic acid	0.107 ± 0.004	0.098 ± 0.004	0.102 ± 0.007	0.006	0.342	0.348
Isopropanol	0.079 ± 0.007	0.075 ± 0.008	0.081 ± 0.005	0.427	0.264	0.683
Ethanol	0.151 ± 0.008	0.140 ± 0.006	0.133 ± 0.013	0.045	0.354	0.027
3-Hydroxybutyric acid	0.798 ± 0.118	0.753 ± 0.082	0.692 ± 0.142	0.543	0.488	0.278
Formic acid	0.005 ± 0.001	0.005 ± 0.001	0.006 ± 0.001	0.285	0.468	0.198
Hypoxanthine	0.006 ± 0.002	0.005 ± 0.002	0.004 ± 0.003	0.526	0.598	0.347
τ-Methylhistidine	0.075 ± 0.035	0.123 ± 0.030	0.169 ± 0.070	0.045	0.248	0.036
Histidine	0.584 ± 0.032	0.592 ± 0.037	0.530 ± 0.076	0.721	0.187	0.224
Hippuric acid	0.063 ± 0.013	0.080 ± 0.016	0.075 ± 0.030	0.123	0.765	0.488
Phenylalanine	0.174 ± 0.013	0.173 ± 0.025	0.218 ± 0.035	0.931	0.058	0.047
Tyrosine	0.214 ± 0.031	0.215 ± 0.024	0.216 ± 0.044	0.955	0.964	0.933
π-Methylhistidine	0.532 ± 0.018	0.578 ± 0.025	0.646 ± 0.074	0.004	0.132	0.019
Threonine	0.298 ± 0.023	0.261 ± 0.021	0.296 ± 0.055	0.023	0.277	0.965
Lactic acid	2.844 ± 0.300	3.363 ± 0.420	4.821 ± 0.778	0.044	0.007	0.001
3-Hydroxyisovaleric acid	0.229 ± 0.018	0.205 ± 0.017	0.250 ± 0.043	0.065	0.086	0.412
Proline	3.285 ± 0.140	2.978 ± 0.130	2.995 ± 0.161	0.002	0.857	0.013
Pyroglutamic acid	0.362 ± 0.042	0.349 ± 0.028	0.375 ± 0.041	0.601	0.335	0.689
Glucose	6.449 ± 0.591	7.571 ± 1.089	7.970 ± 1.421	0.042	0.678	0.043
Serine	0.997 ± 0.044	1.070 ± 0.081	1.055 ± 0.087	0.125	0.812	0.267
Glycerol	0.300 ± 0.047	0.262 ± 0.039	0.273 ± 0.086	0.244	0.827	0.627
Glycine	0.198 ± 0.025	0.225 ± 0.030	0.225 ± 0.050	0.168	0.692	0.364
Arginine	3.505 ± 0.094	3.582 ± 0.110	3.564 ± 0.234	0.303	0.848	0.666
Lysine	3.229 ± 0.112	3.341 ± 0.136	3.354 ± 0.243	0.221	0.937	0.381
2-Oxoglutaric acid	0.010 ± 0.004	0.009 ± 0.003	0.013 ± 0.016	0.624	0.627	0.726
Alanine	0.242 ± 0.048	0.300 ± 0.050	0.513 ± 0.085	0.111	0.001	0.000
Acetic acid	0.088 ± 0.006	0.098 ± 0.007	0.094 ± 0.011	0.062	0.635	0.346
Acetoacetate	0.012 ± 0.006	0.032 ± 0.012	0.023 ± 0.011	0.005	0.327	0.134
Glutamic acid	0.384 ± 0.068	0.402 ± 0.064	0.512 ± 0.072	0.678	0.037	0.017
Glutamine	0.863 ± 0.080	0.828 ± 0.092	0.936 ± 0.158	0.603	0.287	0.448
Pyruvate	0.053 ± 0.003	0.053 ± 0.004	0.062 ± 0.008	0.855	0.081	0.065
N-Acetylglycine	0.132 ± 0.009	0.119 ± 0.014	0.108 ± 0.021	0.131	0.414	0.075
Citric acid	0.006 ± 0.002	0.008 ± 0.002	0.016 ± 0.008	0.196	0.128	0.043
Methionine	0.199 ± 0.008	0.203 ± 0.010	0.211 ± 0.017	0.531	0.415	0.232
Acetone	0.018 ± 0.001	0.018 ± 0.002	0.020 ± 0.002	0.355	0.063	0.167
Aspartic acid	0.136 ± 0.018	0.139 ± 0.014	0.155 ± 0.013	0.823	0.135	0.121
Methylguanidine	0.024 ± 0.002	0.022 ± 0.003	0.026 ± 0.006	0.302	0.223	0.441
Asparagine	1.565 ± 0.112	1.452 ± 0.078	1.220 ± 0.211	0.121	0.084	0.013
Trimethylamine	4.513 ± 0.212	5.007 ± 0.298	4.912 ± 0.576	0.012	0.784	0.265
Sarcosine	0.016 ± 0.004	0.015 ± 0.003	0.021 ± 0.009	0.788	0.276	0.375
Dimethylamine	0.010 ± 0.001	0.009 ± 0.001	0.013 ± 0.002	0.674	0.021	0.027
N,N-Dimethylglycine	6.994 ± 0.217	6.424 ± 0.219	5.423 ± 0.443	0.002	0.001	0.000
Creatine	0.046 ± 0.014	0.069 ± 0.012	0.062 ± 0.022	0.022	0.702	0.224
Dimethyl sulfone	0.034 ± 0.002	0.036 ± 0.002	0.040 ± 0.005	0.401	0.186	0.088
Choline	0.217 ± 0.023	0.216 ± 0.014	0.196 ± 0.028	0.944	0.335	0.309
Phosphocholine	0.245 ± 0.035	0.279 ± 0.032	0.390 ± 0.054	0.189	0.003	0.002
Glycerophosphocholine	0.407 ± 0.036	0.325 ± 0.051	0.395 ± 0.127	0.020	0.502	0.799
Succinic acid	0.062 ± 0.004	0.060 ± 0.004	0.060 ± 0.007	0.403	0.952	0.608
Betaine	0.116 ± 0.033	0.098 ± 0.031	0.076 ± 0.034	0.448	0.421	0.215
Trimethylamine N-oxide	0.236 ± 0.043	0.209 ± 0.030	0.216 ± 0.083	0.225	0.884	0.567
myo-Inositol	1.022 ± 0.029	1.077 ± 0.042	1.057 ± 0.065	0.027	0.587	0.343
Creatinine	0.484 ± 0.018	0.502 ± 0.022	0.472 ± 0.043	0.277	0.208	0.658
Ornithine	8.057 ± 0.245	8.080 ± 0.264	7.454 ± 0.476	0.912	0.037	0.046

SEM means the standard error of the mean, confidence interval.

### Determination of Differential Metabolites Between Different TNBC Patients

**FIGURE 3:**
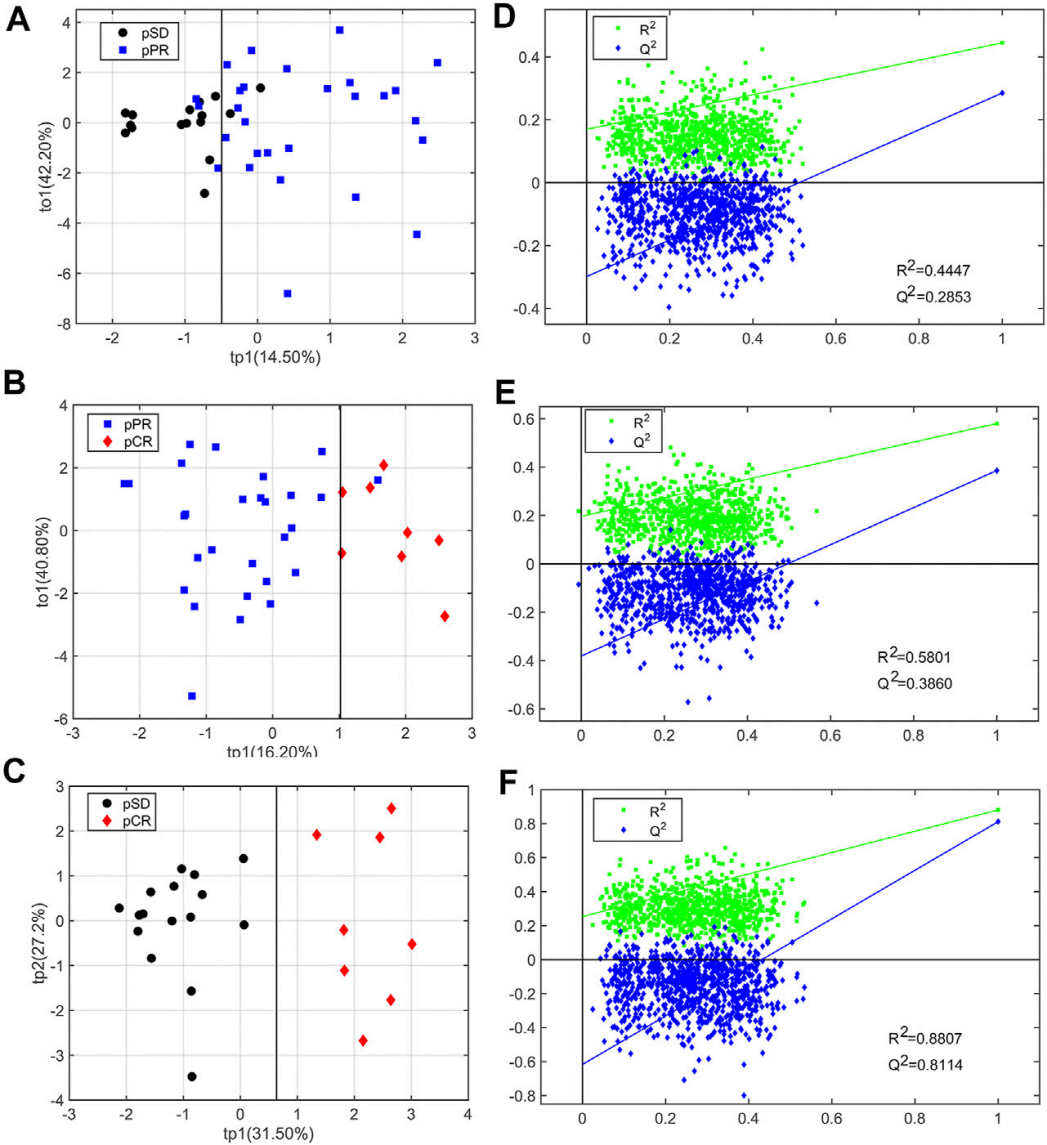
OPLS-DA score plots and corresponding permutation tests of the relative concentration of metabolites’ data from three groups of TNBC patients: **(A, D**) pSD patients vs. pPR patients; **(B, E)** pSD patients vs. pCR patients; **(C, F)** pPR patients vs. pCR patients. In the RPT plots, the green square is R^2^ (cum), denoting the explained variance of the model. The blue diamond is Q^2^ (cum), standing for the predictive ability of the model.

**FIGURE 4:**
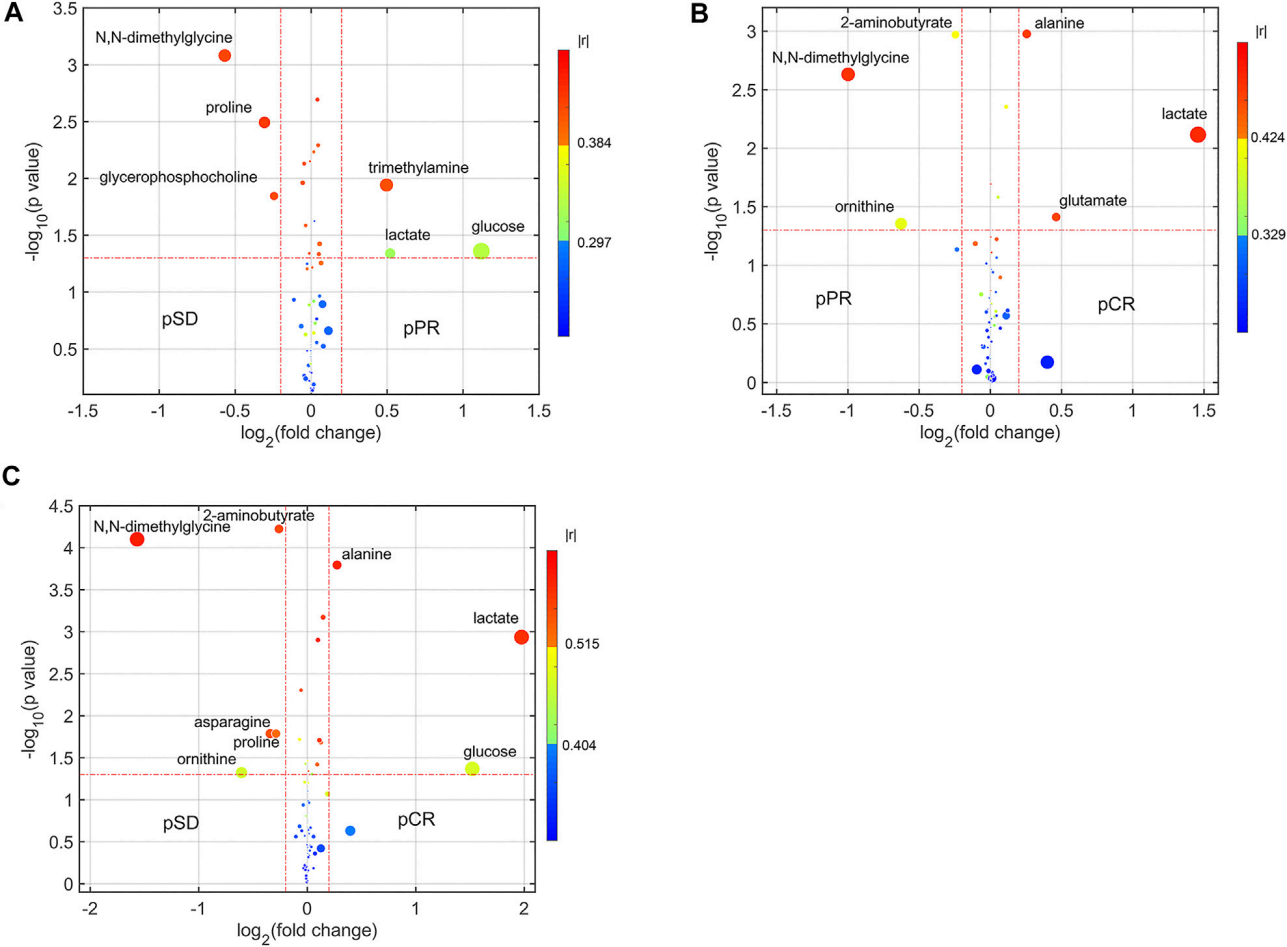
Enhanced volcano plots showing significantly different metabolites: **(A)** pSD patients vs. pPR patients; **(B)** pPR patients vs. pCR patients; **(C)** pSD patients vs. pCR patients. The volcano plot shows log_2_ (fold change) on the *x*-axis and -log_10_ (*p* value) on the y-axis. Each point represents a metabolite. Circles’ size and color are determined based on the variable importance in projection (VIP) and absolute correlation coefficient values (|r|), respectively. For each comparison, the larger the VIP value, the larger the size of the circle, and the warmer color corresponds to higher |r|; the gradient blue means |r| is less than 0.297; the gradual bright yellow means |r| is greater than 0.297 and is less than 0.384; the gradient red means |r| is greater than 0.384.

For analyzing the differential metabolites, the four-dimensional enhanced volcano plots were used for data visualization (Lin et al., 2017). Based on OPLS-DA models, the VIP value and correlation coefficients (r) were calculated. The score plots of OPLS-DA models also showed that the metabolic profiles of different groups (pCR, pPR, and pSD) were differentiable (Figures 3A–C). And the corresponding RPTs demonstrated that the OPLS-DA models were not overfitting (Figures 3D–F). In the enhanced volcano plot (Figure 4), the differential metabolites were determined using the following four criteria: VIP value > 1, *p* value < 0.05, absolute log_2_ (fold change) > 0.2, and correlation coefficient (r) > corresponding threshold (|r|>0.297 in pPR vs. pSD; |r|>0.329 in pCR vs. pPR; |r|>0.404 in pCR vs. pSD). The differential metabolites are located at the upper-left and upper-right areas of the volcano plot with larger circular shapes and gradually warm colors. In Figure 4A (pPR vs. pSD), six metabolites were a significant difference. Compared with the pSD group, three of the metabolites (trimethylamine, glucose, and lactic acid) were increased and three metabolites (N,N-dimethylglycine, proline, and glycerophosphocholine) were decreased in the pPR group. The relevant statistical parameters in Figure 4A are shown in Supplementary Table S3. Compared with the pPR group, three of the metabolites (lactic acid, glutamic acid, and alanine) were increased and three metabolites (2-aminobutanoic acid, N,N-dimethylglycine, and ornithine) were decreased in the pCR group (Figure 4B). Similarly, the relevant statistical parameters in Figure 4B are shown in Supplementary Table S4. Compared with the pSD group, eight metabolites were a significant difference, including three of the metabolites (lactic acid, alanine, and glucose) which increased and five metabolites (2-aminobutanoic acid, N,N-dimethylglycine, asparagine, proline, and ornithine) which decreased in the pCR group (Figure 4C). The relevant statistical parameters in Figure 4C are shown in Supplementary Table S5.

### Significantly Disturbed Metabolic Pathways in Different Groups

**FIGURE 5:**
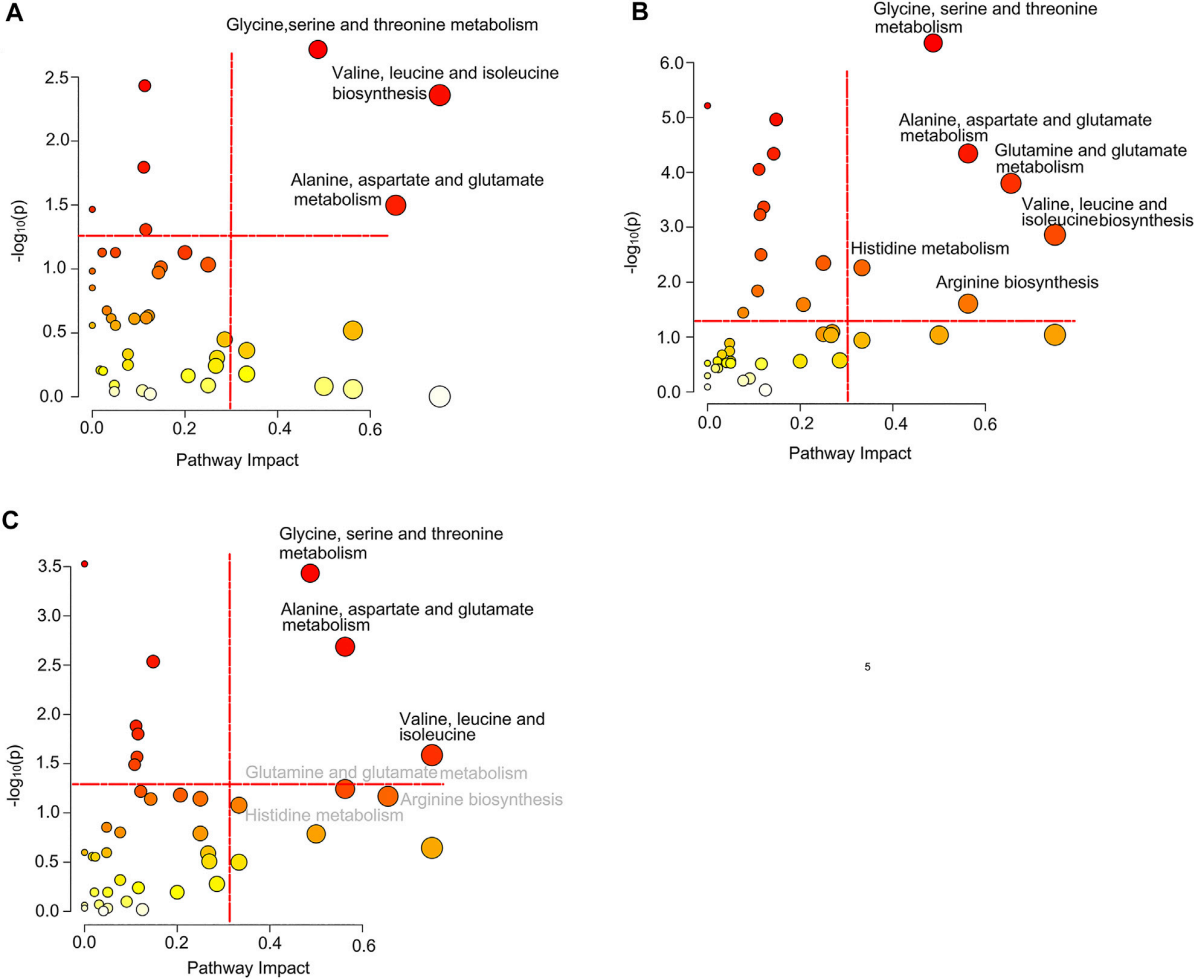
Significantly disturbed metabolic pathways calculated in the comparison of three groups of TNBC patients: **(A)** pSD patients vs. pPR patients; **(B)** pSD patients vs. pCR patients; **(C)** pPR patients vs. pCR patients.

Based on the differential metabolites, we identified significantly disturbed metabolic pathways through pairwise comparison (Figure 5). On comparison between the pSD group and the pPR group, three metabolic pathways were changed, including glycine, serine, and threonine metabolism, valine, leucine and isoleucine biosynthesis, and alanine, aspartate, and glutamate metabolism (Figure 5A). Simultaneously, on comparison between the pCR group and the pSD group, more metabolic pathways were disturbed, including glycine, serine, and threonine metabolism, valine, leucine, and isoleucine biosynthesis, alanine, aspartate, and glutamate metabolism, glutamine and glutamate metabolism, histidine metabolism, and arginine biosynthesis (Figure 5B). On comparison between pCR and pPR groups, we found the disturbed metabolic pathways were the same as the metabolic pathways which were changed in comparison between the pSD and pPR groups (Figure 5C).

### Potential Discriminant Analysis of Disturbed Metabolic Pathways in Different TNBC Patients

**FIGURE 6:**
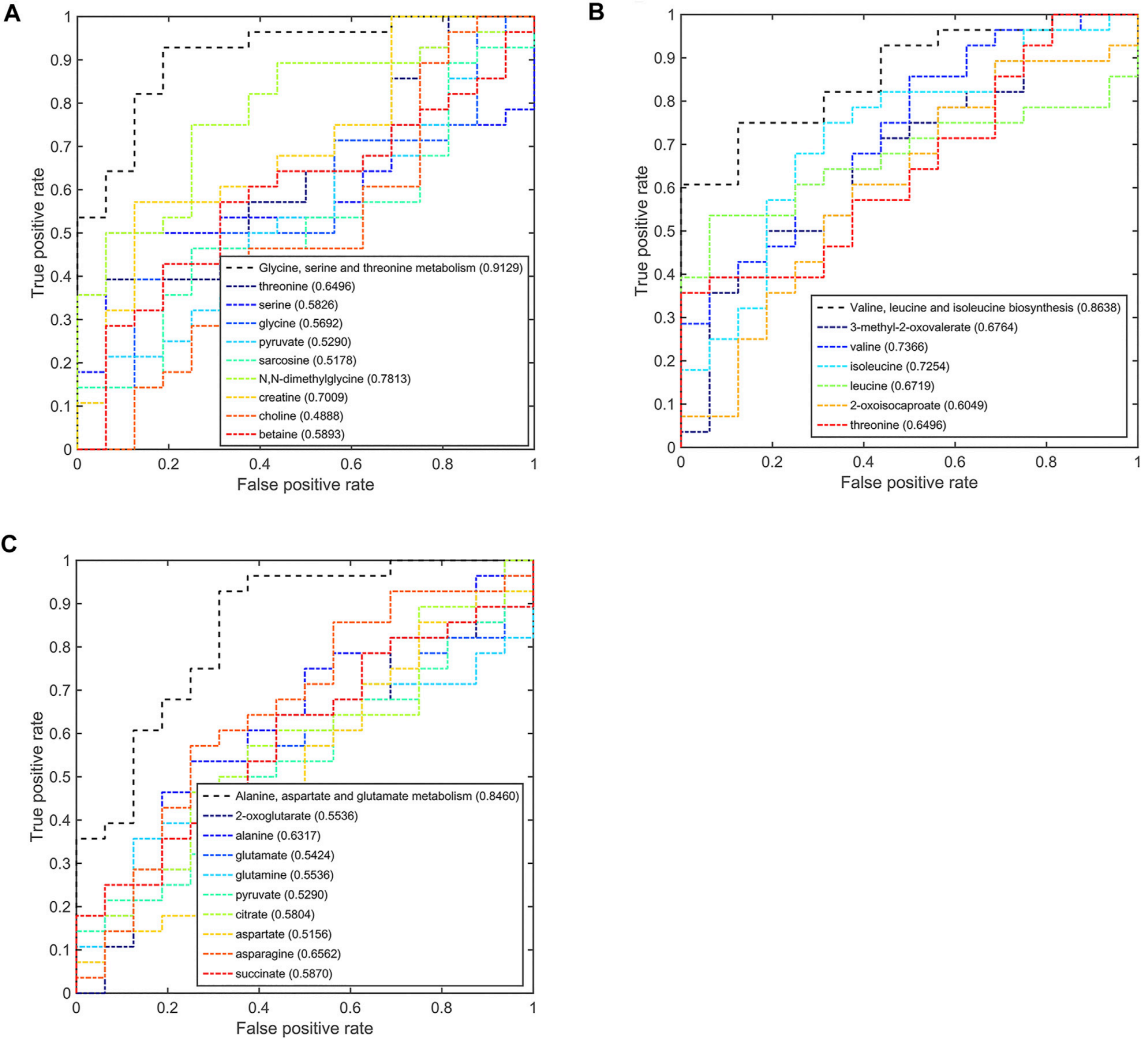
Multi-ROC curves assessing discriminant capabilities of the significantly disturbed metabolic pathways in the pPR patients compared with the pSD patients. The AUC values shown in brackets are used to evaluate the performances of various biomarker models: **(A)** glycine, serine, and threonine metabolism; **(B)** valine, leucine, and isoleucine biosynthesis; **(C)** alanine, aspartate, and glutamate metabolism.

**FIGURE 7:**
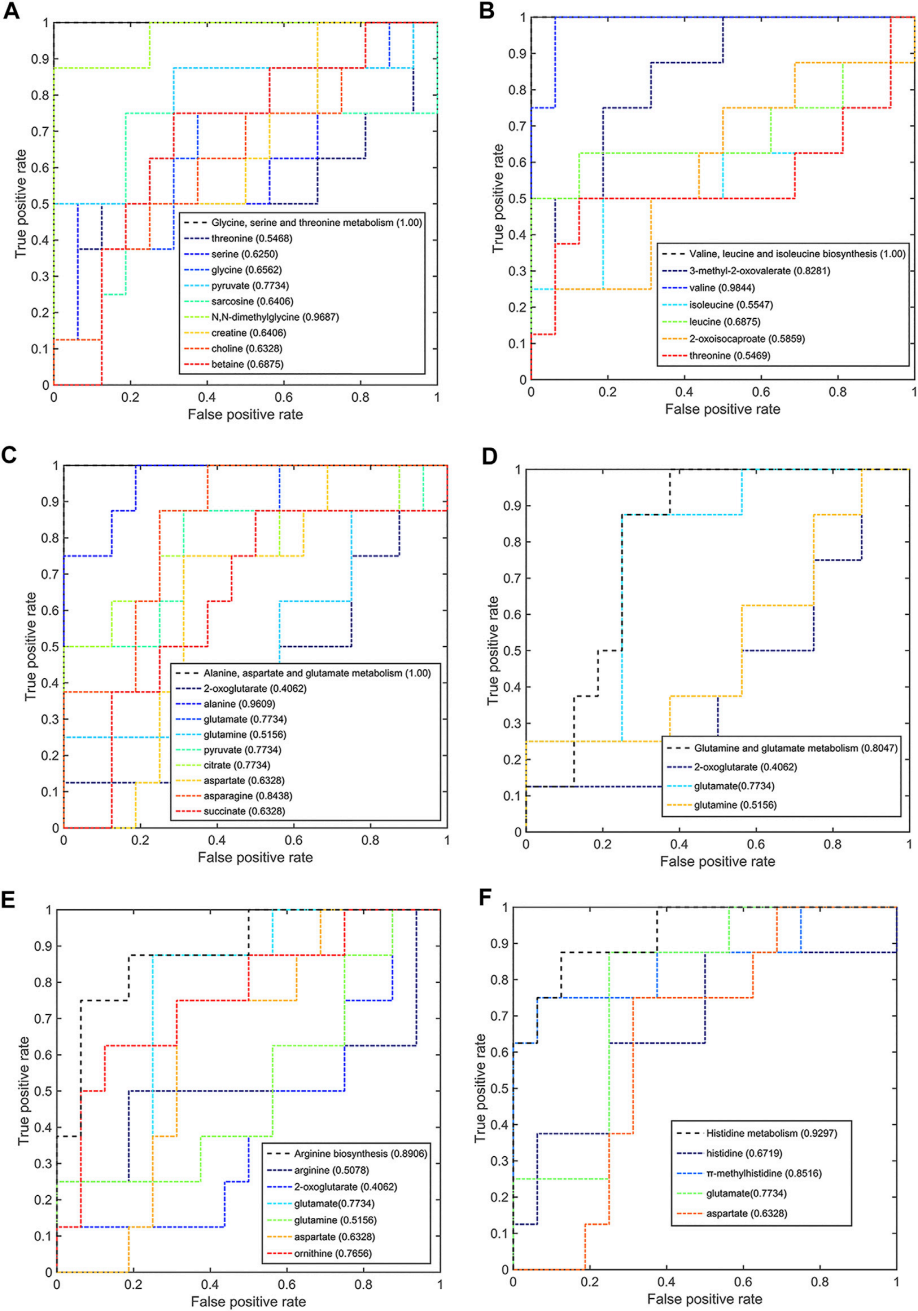
Multi-ROC curves assessing discriminant capabilities of the significantly disturbed metabolic pathways in the pCR patients compared with the pSD patients. The AUC values shown in brackets are used to evaluate the performances of various biomarker models: **(A)** glycine, serine, and threonine metabolism; **(B)** valine, leucine, and isoleucine biosynthesis; **(C)** alanine, aspartate, and glutamate metabolism; **(D)** glutamine and glutamate metabolism; **(E)** arginine biosynthesis; **(F)** histidine metabolism.

**FIGURE 8:**
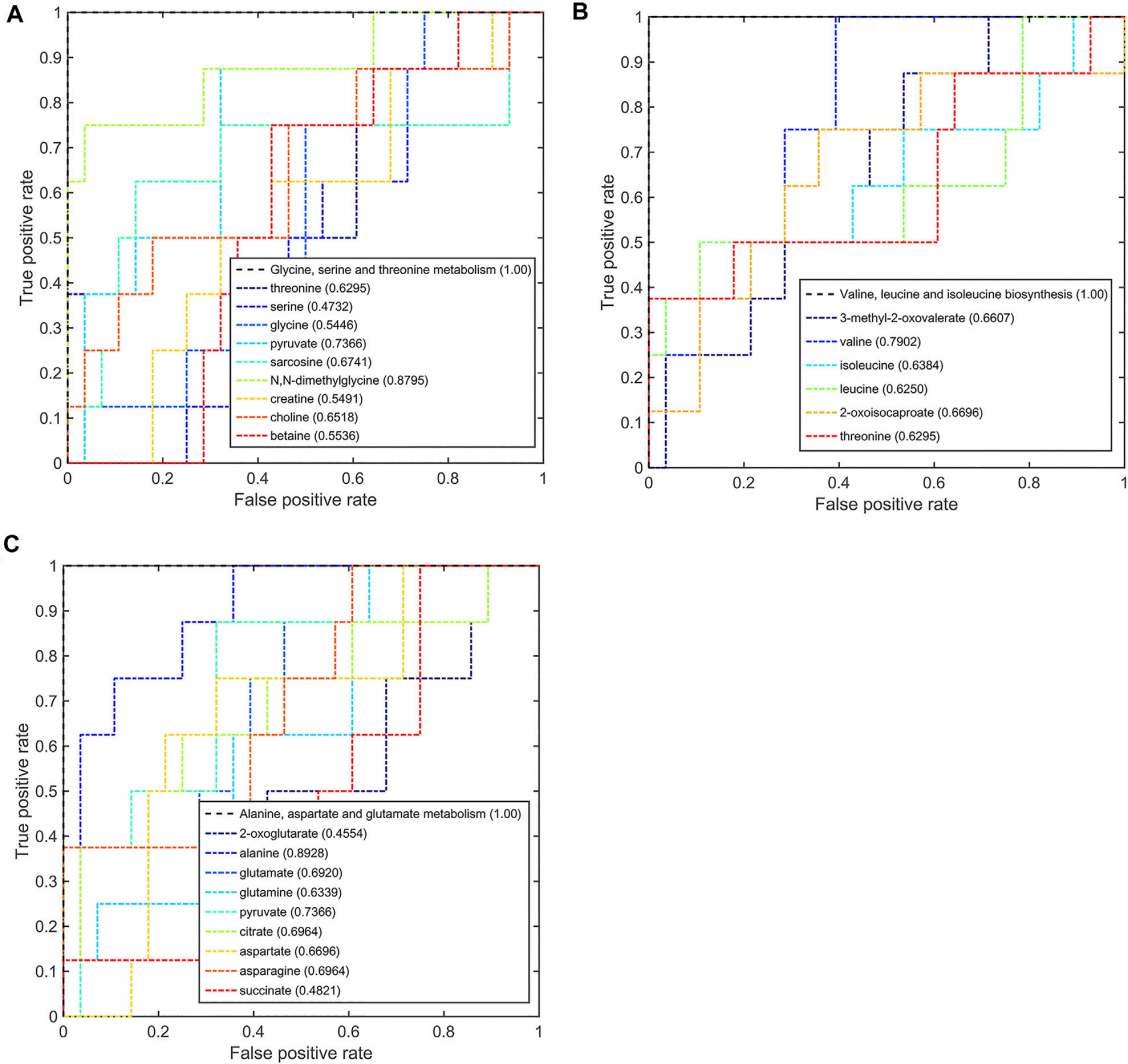
Multi-ROC curves assessing discriminant capabilities of the significantly disturbed metabolic pathways in the pCR patients compared with the pPR patients. The AUC values shown in brackets are used to evaluate the performances of various biomarker models: **(A)** glycine, serine, and threonine metabolism; **(B)** valine, leucine, and isoleucine biosynthesis; **(C)** alanine, aspartate, and glutamate metabolism.

Based on the discriminant capabilities of the significant metabolites predicted from the multivariate ROC curve analysis, we analyzed the potential discriminative ability of disturbed metabolic pathways which could metabolically discriminate the different TNBC groups (Figures 6–8). Compared to the pSD group, three significant pathways displayed good discriminant capabilities in the pPR group with larger AUC values of 0.9129 for glycine, serine, and threonine metabolism, 0.8638 for valine, leucine, and isoleucine biosynthesis, and 0.8460 for alanine, aspartate, and glutamate metabolism (Figure 6). More significantly, the AUC values of N,N-dimethylglycine, valine, isoleucine, and creatine were higher than the threshold (0.7813, 0.7366, 0.7254, and 0.7009) in these pathways. These results showed that N,N-dimethylglycine, valine, isoleucine, and creatine could be used as potential biomarkers to distinguish between the pPR group and the pSD group.

Meanwhile, six significant pathways also showed good discriminant capabilities when the pCR group was compared with the pSD group (Figure 7), of which three of the same significant metabolic pathways showed excellent distinguishing ability (AUC values = 1) and the other three significant metabolic pathways also had a good distinguishing ability with a larger AUC value of 0.8047 for glutamine and glutamate metabolism, 0.8906 for arginine biosynthesis, and 0.9297 for histidine metabolism. The metabolites involved in these significant metabolic pathways also had a good ability to distinguish between pCR and pSD groups. The AUC values of N,N-dimethylglycine and pyruvate were higher than the threshold (0.9687 and 0.7734) in the metabolic pathway of glycine, serine, and threonine metabolism (Figure 7A). Valine and 3-methyl-2-oxovaleric acid had an excellent distinguishing ability with larger AUC values (0.9844 and 0.8281, Figure 7B). In the metabolic pathway of alanine, aspartate, and glutamate metabolism, the metabolites of alanine, glutamic acid, pyruvate, citric acid, and asparagine had AUC values that exceed the threshold (0.9609, 0.7734, 0.7734, 0.7734, and 0.8438, Figure 7C). The same, the metabolite of glutamic acid showed the same discriminative ability in the metabolic pathway of glutamine and glutamate metabolism (Figure 7D). Similarly, ornithine had good discriminative ability in the metabolic pathway of arginine biosynthesis with 0.7856 AUC value (Figure 7E), and π-methylhistidine had good discriminative ability in the metabolic pathway of histidine metabolism with 0.8516 AUC value (Figure 7F). According to this multi-ROC curve analysis, the metabolites of N,N-dimethylglycine, pyruvate, valine, 3-methyl-2-oxovaleric acid, citric acid, asparagine, ornithine, and π-methylhistidine could be used as potential biomarkers to distinguish between the pCR group and the pSD group.

On comparison between the pCR group and pPR group patients, these three significant pathways also had a good discriminative ability with larger AUC values (Figure 8). In the metabolic pathway of glycine, serine, and threonine metabolism, the AUC values of N,N-dimethylglycine and pyruvate were higher than the threshold (0.8795 and 0.7366, Figure 8A). In the metabolic pathway of valine, leucine, and isoleucine biosynthesis, valine was the metabolite that mainly contributed to the discriminative ability with a larger AUC value (0.7902, Figure 8B). In the metabolic pathway of alanine, aspartate, and glutamate metabolism, only alanine and pyruvate contributed to the discriminative ability of this metabolic pathway (0.8928 and 0.7366, Figure 8C). According to this multi-ROC curve analysis, N,N-dimethylglycine, pyruvate, valine, and alanine could be used as potential biomarkers to distinguish between the pCR group and the pPR group.

According to the AUC values from the multi-ROC curves by differential metabolites, the AUC values of the pairwise comparison of N,N-dimethylglycine and valine were greater than 0.7. These two metabolites (N,N-dimethylglycine and valine) could be utilized as most potential biomarkers to distinguish among these three groups.

## Discussion

In this experimental study, we used the NMR-based metabolomics technology to predict the different sensitivities of NAC for TNBC patients. Our study found that the metabolic phenotype could classify the sensitivity of NAC, although each group of TNBC patients has different clinical and histopathological parameters. Some studies used transcriptomics to study the chemotherapy sensitivity of TNBC. Ozge Saatci *et al.* found lysyl-oxidase (LOX) to be a key inducer of chemoresistance in TNBC by whole-transcriptome sequencing (RNA-seq) (Saatci et al., 2020). In the same way, some research studies used metabolomics technology to analyze the predictive aspects of NAC of cancer (Wei et al., 2013; Hou et al., 2014; Yang et al., 2018). Meanwhile, our research was more focused on the differences in the overall metabolic pathways to distinguish the three groups of TNBC patients.

In our study, the resulting prediction models (OPLS-DA models) have high sensitivity and specificity. Through pairwise comparisons (pSD vs. pCR; pSD vs. pPR; pPR vs. pSD) by using the AUC values in multi-ROC curve analysis, three significant metabolic pathways of glycine, serine, and threonine metabolism, valine, leucine, and isoleucine biosynthesis, and alanine, aspartate, and glutamate metabolism could distinguish three groups in pairs. The metabolic pathway of glycine, serine, and threonine metabolism is linked to human BC invasion by comparing metabolic profiling of BC cells with different metastatic potentials (Kim S. et al., 2016). N,N-Dimethylglycine is involved in glycine, serine, and threonine metabolism, and also the methylation product of glycine. Biochemical methylation reaction mediates the transfer of methyl groups and regulates life activities. Ming Zhang *et al.* found the seven differentially methylated sites (DMSs) that were highly correlated with cell cycle as potential specific diagnostic biomarkers for BC patients (Zhang et al., 2020). Branched-chain amino acid metabolism was reprogrammed during tumorigenesis in many types of human cancers (Holecek 2018; Peng et al., 2020; Sivanand and Vander Heiden, 2020), including glioblastoma (Zhang et al., 2021), non-small-cell lung cancer (NSCLC) (Mayers et al., 2016), BC (Zhang, 2017), and ovarian cancer (Wang et al., 2015). In our work, we found the patients with different responses to NAC (pSD, pPR, and pCR) had different reprogramming metabolic pathways of valine, leucine, and isoleucine biosynthesis. Alanine, aspartate, and glutamate metabolism was reported to function as an alternative carbon source that fuels tumor metabolism (Sousa et al., 2016).

On the contrary, the other three metabolic pathways only could be used to distinguish between the pCR group and the pSD group. Glutamine and glutamate metabolism was perturbed in many types of cancers (Mates et al., 2019). Glutamine and glutamate metabolism has indispensable functions to provide amino acids, lipids, nucleotides, hexosamines, and polyamines, but also to render metabolic energy (ATP) (Mates et al., 2020). Meanwhile, glutamine and glutamate metabolism could regulate glutathione (GSH), the most important intracellular antioxidant molecule (Mates et al., 2018). Cancer cells frequently increase oxidative damage in response to changes in glutamine and glutamate metabolism (Mates et al., 2020). In our study, the significant metabolic pathway of glutamine and glutamate metabolism could distinguish different metabolic phenotypes between the pCR group and the pPR group. This result indicates that the oxidative stress state of the BC patients’ microenvironment is different. Arginine biosynthesis was linked to the metabolic regulation of nitric oxide synthesis in cancer (Keshet and Erez, 2018). Paniz Jasbi *et al.* also found the arginine/proline metabolism was disturbed in the BC patients by using the targeted plasma metabolomics (Jasbi et al., 2019). Von Mach-Szczypiński *et al.* found histidine metabolism was abnormal in tissues of primary ductal BC (von Mach-Szczypinski et al., 2009a; von Mach-Szczypinski et al., 2009b). Similarly, histidine metabolism was abnormal in the serum of primary ductal BC (Sieja et al., 2005). Our research results also verify this result.

Since the chemotherapy response prediction for cancer remains challenging around the world, this promising metabolomics approach might open a new view for patients to select the promising treatment or even a truly “personalized treatment” in clinical practice. Compared with other studies on single or multiple molecules as potential biomarkers, our research was more focused on the overall differences in metabolic pathways or metabolic phenotypes as potential biomarkers.

Our study analyzed differences in the metabolic phenotypes of TNBC patients with different sensitivity to neoadjuvant chemotherapy by using NMR-based metabolomics and then constructed a prediction model based on the metabolic phenotype. Three significant metabolic pathways of glycine, serine, and threonine metabolism, valine, leucine, and isoleucine biosynthesis, and alanine, aspartate, and glutamate metabolism could distinguish groups of patients with no, partial, or complete response. Additional three significant metabolic pathways of glutamine and glutamate metabolism, arginine biosynthesis, and histidine metabolism could distinguish groups of patients with no or complete response. Although this study only involved a small number of patient cohorts, the results have shown that these several metabolic pathways have good distinguishing ability for different patients with no, partial, or complete response. Of course, we need more clinical cohort samples for verifying these results. This method could be used as a preoperative choice for efficacy evaluation for patients with BC neoadjuvant chemotherapy.

## Limitation

This study has the limitation that we lack of verification of other data of transcriptomics, proteomics, etc. Furthermore, given our relatively small sample size, our observation still remains to be verified in a large cohort. Thus, follow-up studies involving long-term studies of a large cohort of TNBC patients receiving NAC are required.

## Data Availability

The data presented in the study are deposited in the Mendeley Data repository, accession number DOI: 10.17632/bbz8353xwh.1
