# Supplementary material for: NMR-Based Metabolomics Analysis Predicts Response to Neoadjuvant Chemotherapy for Triple-Negative Breast Cancer
Source: Front Mol Biosci. 2021 Nov 2;8:708052. doi: 10.3389/fmolb.2021.708052 (PMC8592909; doi:10.3389/fmolb.2021.708052)
Supplement: Supplementary file 1 [file DataSheet1.pdf]

Supplemental Materials

NMR-based Metabolomics Analysis Predict Response to Neoadjuvant Chemotherapy for Triple Negative Breast Cancer

Xiangming He, Jinping Gu, Dehong Zou, Hongjian Yang, Yongfang Zhang, Yuqing Ding, Lisong Teng

4 Supplementary Figures

5 Supplementary Tables

Supplementary Figure Legend

Figure S1. Reporter signals (dark red) from the metabolite library, target signals (grey) from experimental spectra and their corresponding target positions indicated by arrows. Therefore, the positions of the library signals were adjusted to be in close agreement with the experimentally observed signals. Metabolites have been arranged in the predetermined order, as it is used for the manual quantification procedure.

Figure S2. PLS-DA scores plots and corresponding permutation tests of relative concentration of metabolites data from three group of TNBC' patients. (A, D) pSD patients vs. pPR patients; (B, E) pSD patients vs. pCR patients; (C, F) pPR patients vs. pCR patients. In the PRTs plots, the green square is R^2 (cum), denoting the explained variance of the model. The blue diamond is Q^2 (cum), standing for the predictive ability of the model.

Figure S3. The comparison of relative intensities of differential metabolites between the three groups.

Figure S4. The typical HSQC spectrum of serum sample.

Table S1. Summary of clinical and pathological features of the TNBC patients' studied

Patient features	pSD	pPR	pCR	pPR vs. pSD	pPR vs. pCR	pCR vs. pSD
Number of patients	16	28	8	/	/	/
Age	29-65	32-63	33-62	0.543	0.339	0.776
Average Age	47.8	49.2	46.7	/	/	/
BMI	20.1-29.4	19.2-29.3	18.7-30.3	0.334	0.678	0.567
Average BMI	26.72	27.88	27.16	/	/	/
Tumor stage						
Stage I	0	0	1	/	/	/
Stage II	2	4	2	/	/	/
Stage III	7	10	1	/	/	/
Stage IV	7	14	4	/	/	/

Table S2. The information of the metabolites in the NMR spectrum

No.	Metabolite Name	HMDB No.	Library signals; ^{1}H/ppm & ^{13}C/ppm
1	2-hydroxybutyric acid	HMDB0000008	(0.886, 11.31); (1.649, 29.81); (1.722, 29.81); (3.990, 76.17)
2	2-hydroxyisovaleric acid	HMDB0000407	(0.793, 18.26); (0.848, 18.26); (0.955, 21.27); (2.005, 34.16); (3.837, 79.98)
3	isocaproic acid	HMDB0000689	(0.864, 24.42); (1.506, 30.01); (1.435, 37.68); (2.177, 38.34)
4	3-methyl-2-oxovaleric acid	HMDB0000491	(0.878, 13.29); (1.059, 16.39); (1.121, 16.39); (1.459, 27.03); (1.690, 27.03); (2.925, 46.41)
5	isovaleric acid	HMDB0000718	(0.896, 24.67); (1.938, 28.84); (2.038, 49.91)
6	valine	HMDB0000883	(0.976, 19.41); (1.031, 20.75); (2.258, 31.89); (3.599, 63.34)
7	isoleucine	HMDB0000172	(0.926, 13.91); (0.995, 17.37); (1.245, 27.24); (1.453, 26.99); (1.962, 38.68); (3.653, 65.52)
8	leucine	HMDB0000687	(0.950, 24.36); (1.703, 42.59); (3.741, 56.21)
9	2-aminobutanoic acid	HMDB0000452	(0.969, 11.08); (1.893, 26.60); (3.701, 58.78)
10	2-oxoisocaproate	HMDB0000695	(0.929, 24.44); (2.088, 26.67); (2.599, 50.82)
11	isobutyric acid	HMDB0001873	(1.049, 22.01); (2.375, 39.57)
12	propionic acid	HMDB0000237	(1.038, 12.98); (2.168, 33.48)
13	isopropanol	HMDB0000863	(1.159, 26.36); (4.006; 67.05)
14	ethanol	HMDB0000108	(1.172, 19.59); (3.644, 60.31)
15	3-hydroxybutyric acid	HMDB0000011	(1.192, 24.40); (2.302, 49.13); (2.399, 49.09); (4.147, 68.39)
16	formic acid	HMDB0000142	(8.390, 172.41)
17	hypoxanthine	HMDB0000157	(7.977, 144.44); (8.115, 140.07)
18	τ-methylhistidine	HMDB0000001	(3.064, 31.14); (3.161, 31.23); (3.684, 36.07); (3.957, 57.64); (7.001, 122.46); (7.671, 141.12)
19	histidine	HMDB0000177	(3.208, 30.08); (3.267, 30.20); (3.294, 30.20); (4.004, 57.28); (7.136, 119.99); (8.025, 138.36)
20	hippuric acid	HMDB0000714	(3.956, 46.75); (7.543, 131.54); (7.625, 134.91); (7.821, 129.94)
21	phenylalanine	HMDB0000159	(3.109, 39.15); (3.274, 39.15); (3.975, 58.93); (7.315, 132.12); (7.366, 130.43); (7.419, 131.81)
22	tyrosine	HMDB0000158	(3.0695, 38.27); (3.175, 38.27); (3.933, 58.98); (6.892, 118.89); (7.194, 133.48)

23	π-methylhistidine	HMDB0000479	(3.218, 27.67); (3.275, 27.67); (3.701, 34.48); (3.933, 56.19); (7.054, 127.20); (7.937, 141.14)
24	threonine	HMDB0000167	(1.316, 22.30); (3.571, 63.46); (4.244, 68.91)
25	lactic acid	HMDB0000190	(1.336, 22.91); (4.125, 71.11)
26	3-hydroxyisovaleric acid	HMDB0000754	(1.293, 30.67); (2.386, 52.10)
27	proline	HMDB0000162	(1.992, 26.45); (2.067, 31.84); (2.344, 31.72); (3.323, 48.96); (3.407, 48.96); (4.127, 64.04)
28	pyroglutamic acid	HMDB0000267	(2.010, 27.98); (2.385, 32.28); (2.488, 27.98); (4.162, 60.97)
29	glucose	HMDB0000122	(3.226, 77.02); (3.398, 72.45); (3.458, 78.64); (3.521, 74.32); (3.720, 63.53); (3.701, 75.60); (3.813, 74.15); (3.821, 63.32); (3.889, 63.39); (5.221, 94.88)
30	serine	HMDB0000187	(3.832, 59.18); (3.954, 63.08)
31	glycerol	HMDB0000131	(3.556, 65.41); (3.646, 65.49); (3.773, 74.97)
32	glycine	HMDB0000123	(3.547, 44.30)
33	arginine	HMDB0000517	(1.680, 26.45); (1.906, 30.49); (3.235, 43.32); (3.761, 57.26)
34	lysine	HMDB0000182	(1.430, 24.04); (1.718, 29.15); (1.884, 32.65); (3.017, 42.12); (3.747, 57.45)
35	2-oxoglutaric acid	HMDB0000208	(2.423, 33.42); (2.997, 38.63)
36	alanine	HMDB0000161	(1.436, 19.01); (1.490, 19.03); (3.775, 53.56)
37	acetic acid	HMDB0000042	(1.906, 26.09)
38	acetoacetate	HMDB0000060	(2.271, 32.25); (3.432, 56.18)
39	glutamic acid	HMDB0000148	(2.085, 29.82); (2.340, 36.36); (3.742, 57.64)
40	glutamine	HMDB0000641	(2.122, 29.28); (2.439, 33.93); (3.758, 57.23)
41	pyruvate	HMDB0000243	(2.359, 29.22)
42	N-acetylglycine	HMDB0000532	(2.027, 24.60); (3.737, 46.05)
43	citric acid	HMDB0000094	(2.521, 48.71); (2.659, 48.71)
44	methionine	HMDB0000696	(2.103, 16.56); (2.179, 32.72); (2.634, 31.58); (3.848, 56.84)
45	acetone	HMDB0001659	(2.222, 32.92)
46	aspartic acid	HMDB0000191	(2.645, 39.35); (2.667, 39.34); (2.689, 39.33); (2.712, 39.33); (2.803, 39.48); (2.843, 39.39); (3.908, 55.08)

47	methylguanidine	HMDB0001522	(2.812, 30.079)
48	asparagine	HMDB0000168	(2.843, 37.35); (2.943, 37.43); (3.993, 54.13)
49	trimethylamine	HMDB0000906	(2.882, 47.47)
50	sarcosine	HMDB0000271	(3.947, 58.36); (4.085, 65.43); (4.167, 65.41)
51	dimethylamine	HMDB0000087	(2.711, 37.30)
52	N,N-dimethylglycine	HMDB0000092	(2.912, 46.34); (3.709, 62.66)
53	creatine	HMDB0000064	(3.027, 39.50); (3.918, 56.43)
54	dimethyl sulfone	HMDB0004983	(3.140, 44.24)
55	choline	HMDB0000097	(3.187, 56.70), (3.502, 70.15); (4.052, 58.49)
56	phosphocholine	HMDB0001565	(3.197, 56.52); (3.575, 68.90); (4.149, 60.61)
57	glycerophosphocholine	HMDB0000086	(3.218, 56.64); (3.610, 64.63); (3.675, 64.67); (3.670, 68.57); (3.903, 73.31); (3.874, 69.14); (3.945, 69.23); (4.321, 62.07)
58	succinic acid	HMDB0000254	(2.394, 36.83)
59	betaine	HMDB0000043	(3.252, 55.86); (3.889, 68.64)
60	trimethylamine N-oxide	HMDB0000925	(3.255, 62.19)
61	myo-Inositol	HMDB0000211	(3.270, 77.16); (3.525, 73.96); (3.608, 75.13); (4.055, 74.93)
62	creatinine	HMDB0000562	(3.039, 32.97); (4.053, 59.24)
63	ornithine	HMDB0000214	(1.741, 25.52); (1.813, 25.52); (1.926, 30.27); (3.047, 41.83); (3.766, 57.05)

Table S3. Significant metabolites with four statistical parameters in the pSD vs. pPR, including *p* value, log_2(FC), VIP and p(corr).

	p value	log_2(FC)	VIP	p(corr)
N,N-dimethylglycine	0.001	-1.484	2.620	-0.628
proline	0.003	-1.237	2.213	-0.703
glycerophosphocholine	0.014	-1.183	1.199	-0.609
trimethylamine	0.011	1.408	2.859	0.581
lactate	0.046	1.434	1.781	0.316
glucose	0.043	2.177	4.450	0.333

Table S4. Significant metabolites with four statistical parameters in the pPR vs. pCR, including *p* value, log_2(FC), VIP and p(corr).

	p value	log_2(FC)	VIP	p(corr)
2-aminobutyrate	0.000	-1.199	1.294	-0.694
N,N-dimethylglycine	0.000	-2.971	3.464	-0.879
ornithine	0.048	-1.519	2.105	-0.473
alanine	0.000	1.207	1.403	0.846
lactate	0.001	3.937	3.580	0.795
glutamate	0.021	1.093	0.807	0.534

Table S5. Significant metabolites with four statistical parameters in the pSD vs. pCR, including *p* value, log_2(FC), VIP and p(corr).

	p value	log_2(FC)	VIP	p(corr)
2-aminobutyrate	0.001	-1.186	1.081	-0.422
N,N-dimethylglycine	0.002	-2.002	3.070	-0.749
asparagine	0.074	-1.175	0.857	-0.320
proline	0.868	1.012	0.613	0.096
ornithine	0.044	-1.543	2.372	-0.406
alanine	0.001	1.193	1.323	0.704
lactate	0.008	2.746	4.206	0.792
glucose	0.668	1.318	2.974	-0.065

Figure S1

2-hydroxybutyric acid

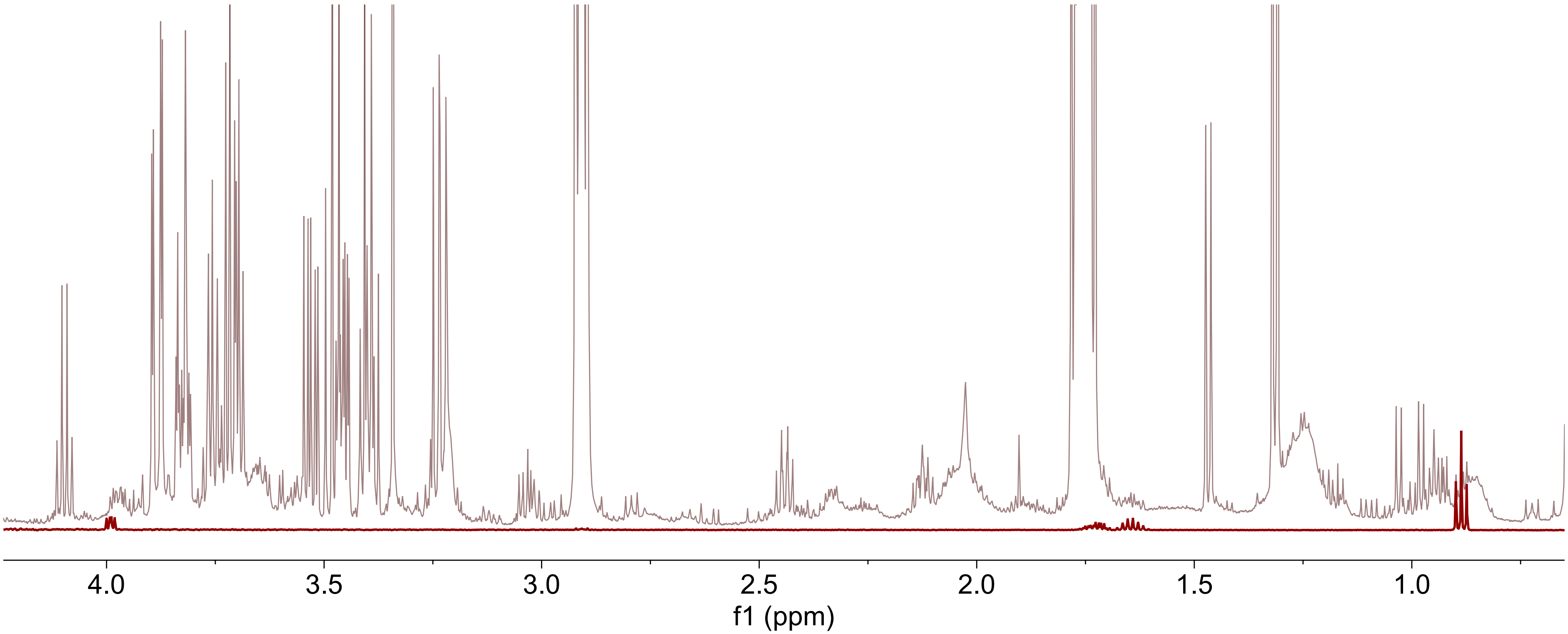

2-hydroxyisovaleric acid

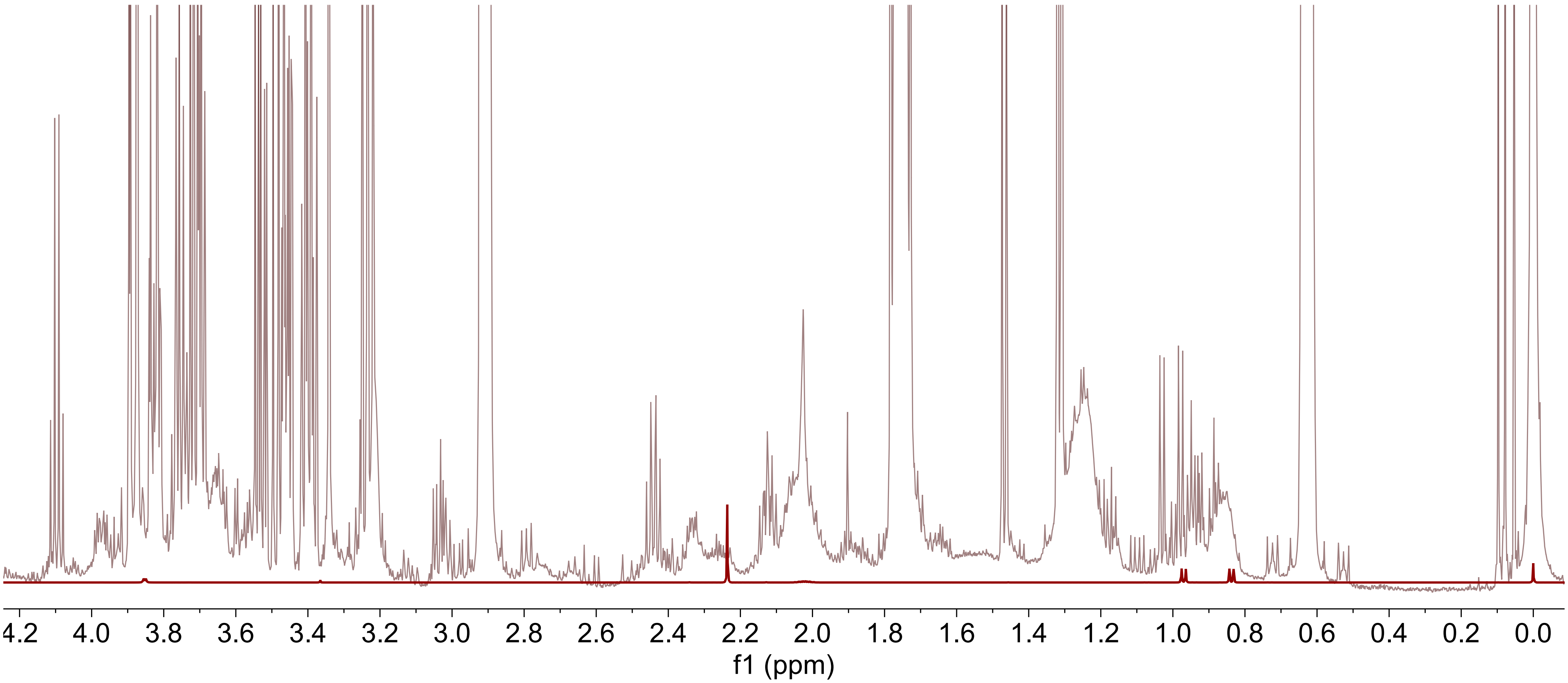

isocaproic acid

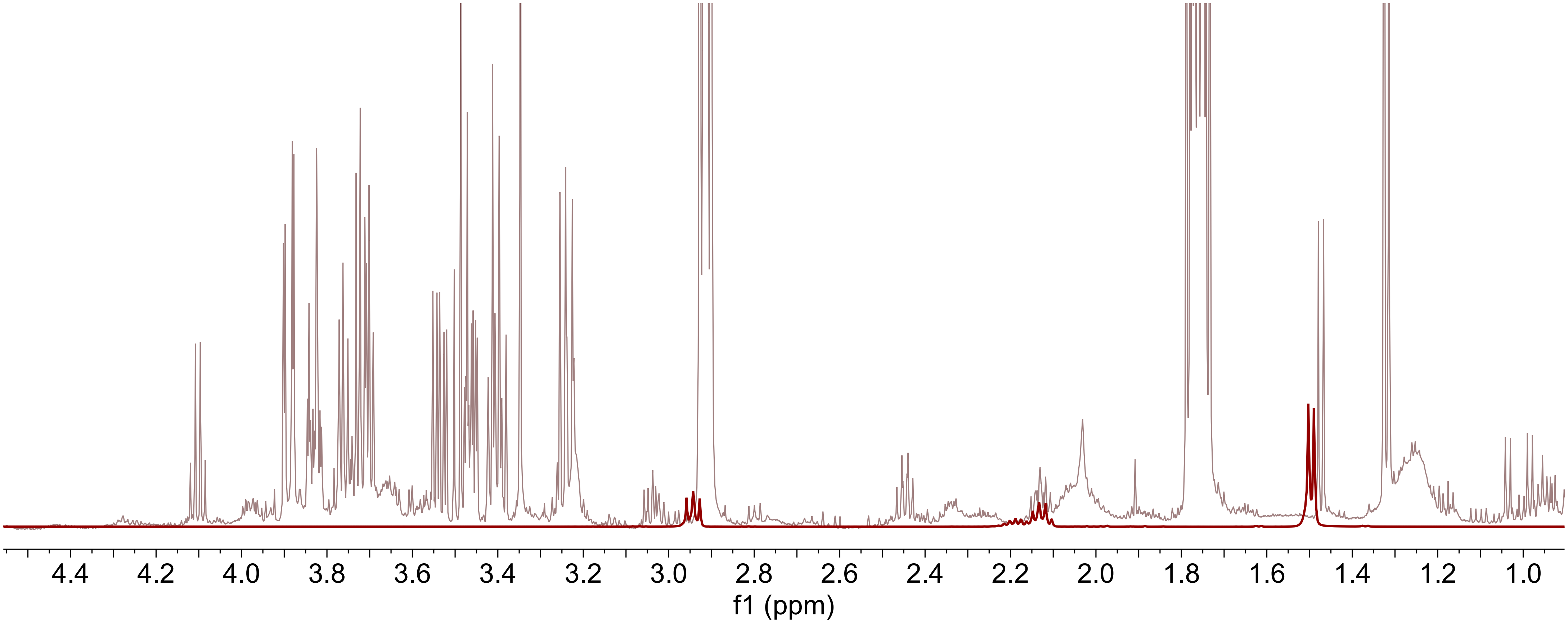

3-methyl-2-oxovaleric acid

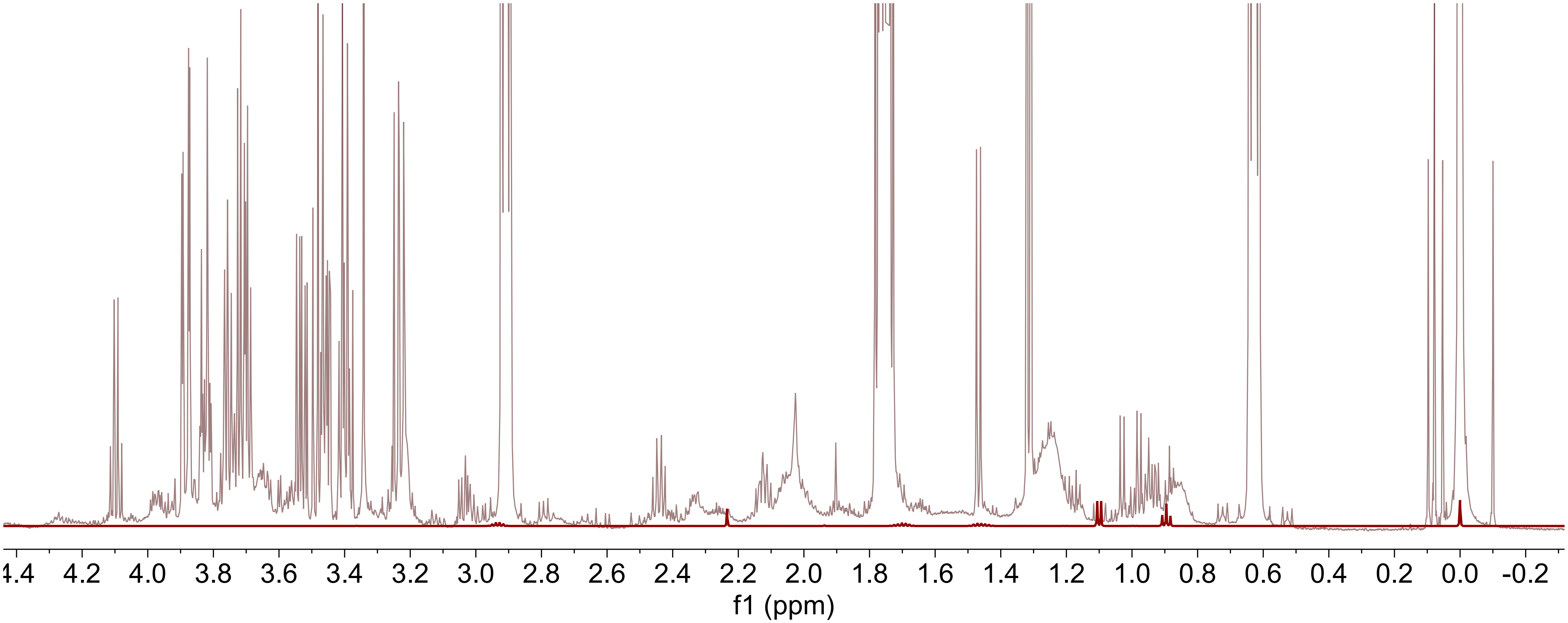

isovaleric acid

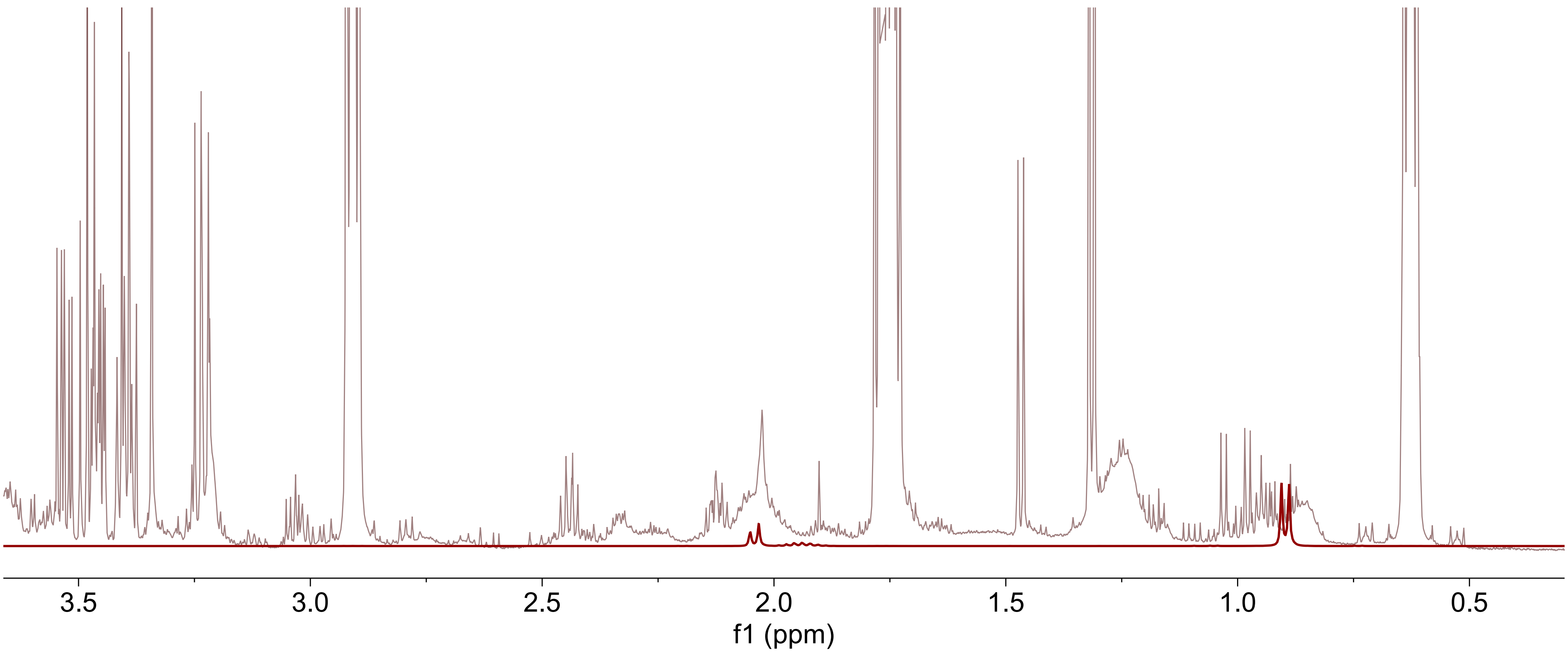

valine

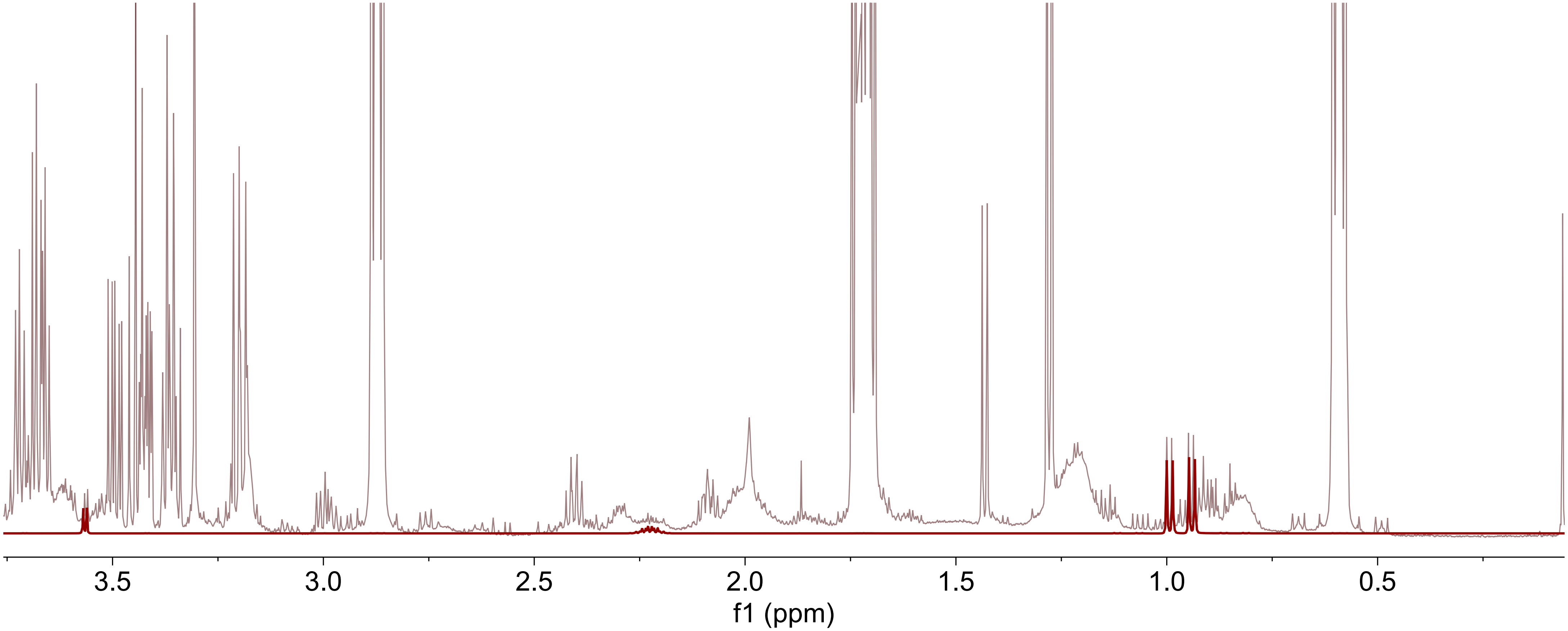

isoleucine

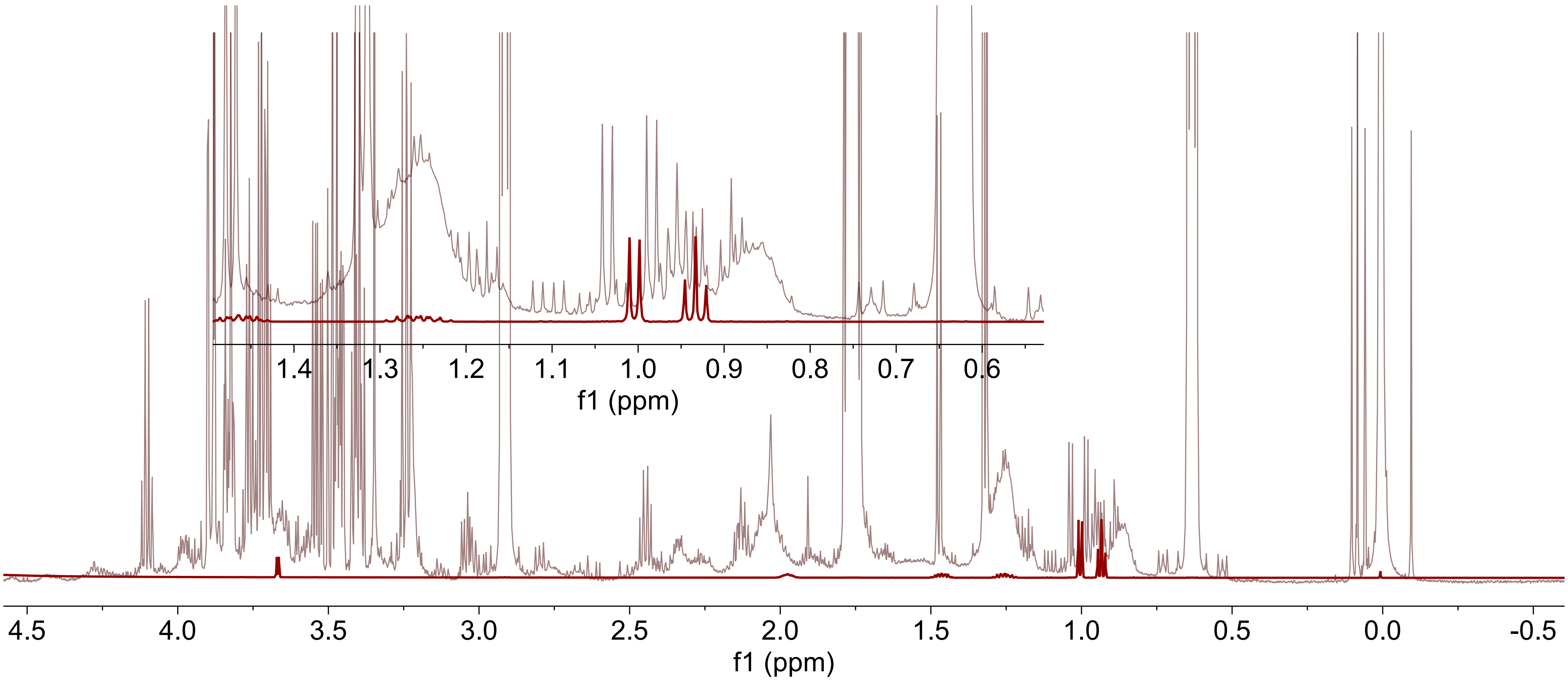
1.4
1.3
1.2
1.1
1.0
0.9
0.8
0.7
0.6
f1 (ppm)
4.5
4.0
3.5
3.0
2.5
2.0
1.5
1.0
0.5
0.0
-0.5
f1 (ppm)

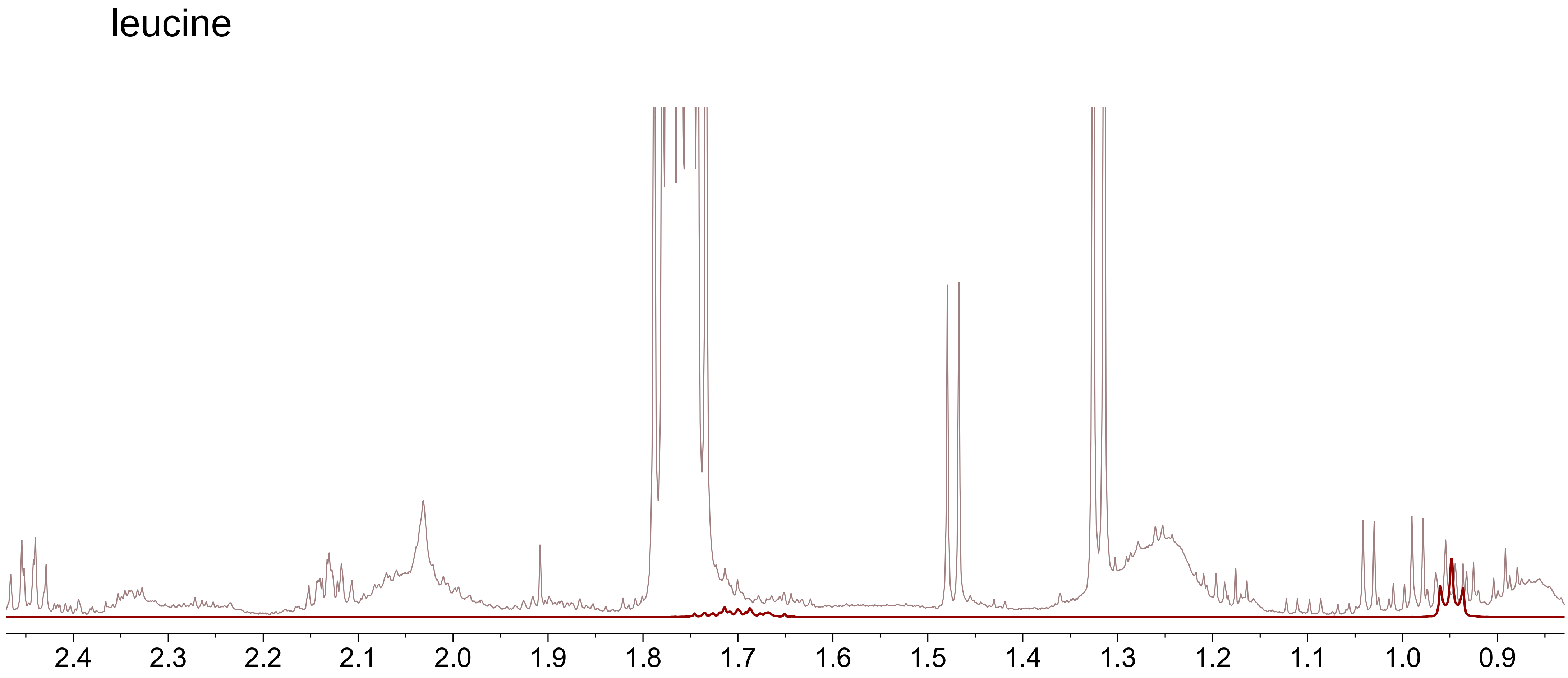

leucine
2.4
2.3
2.2
2.1
2.0
1.9
1.8
1.7
1.6
1.5
1.4
1.3
1.2
1.1
1.0
0.9

2-aminobutanoic acid

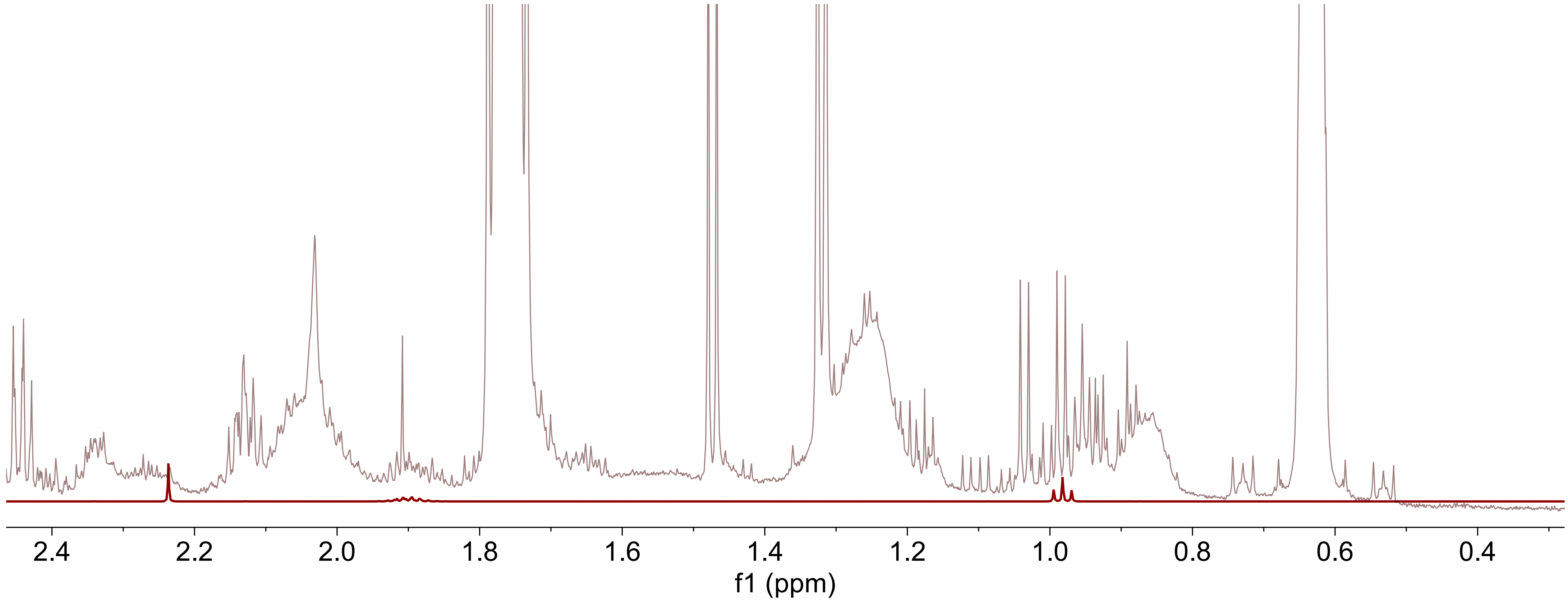

2-oxoisocaproate

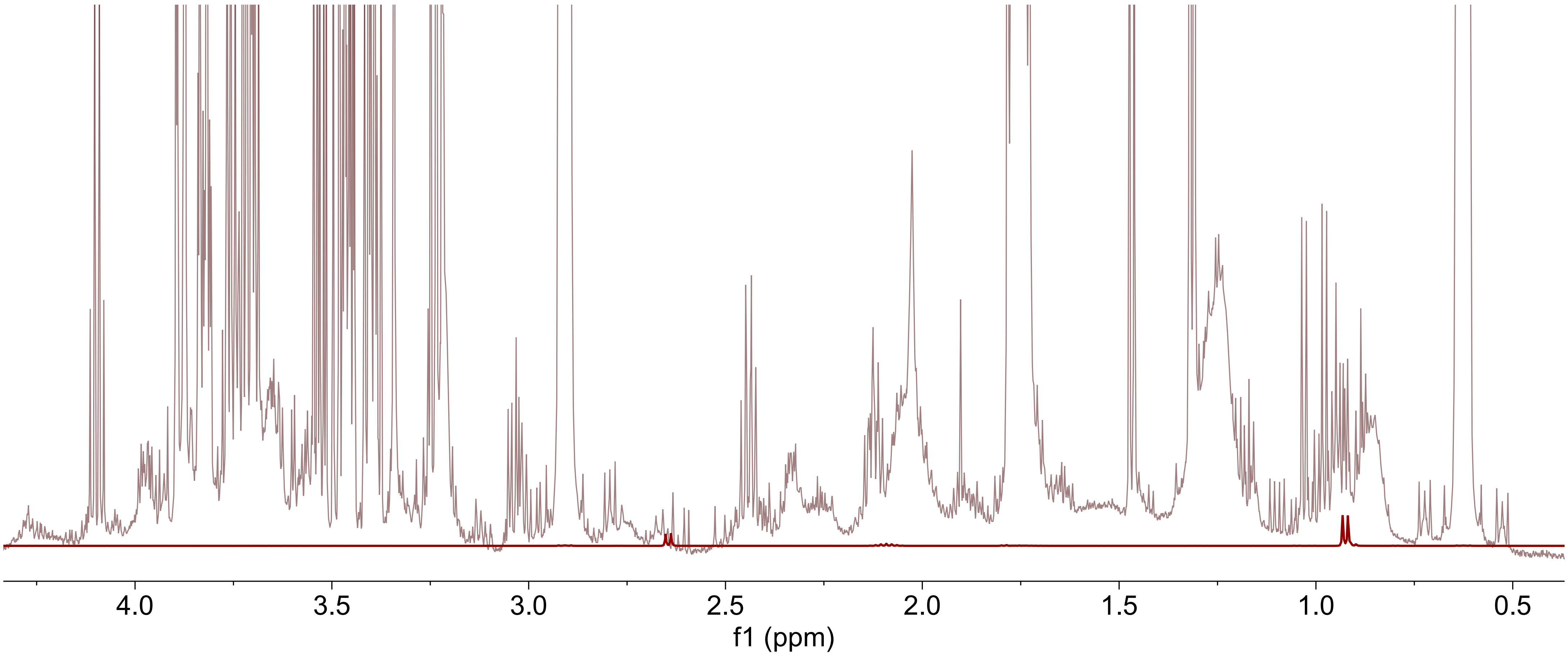

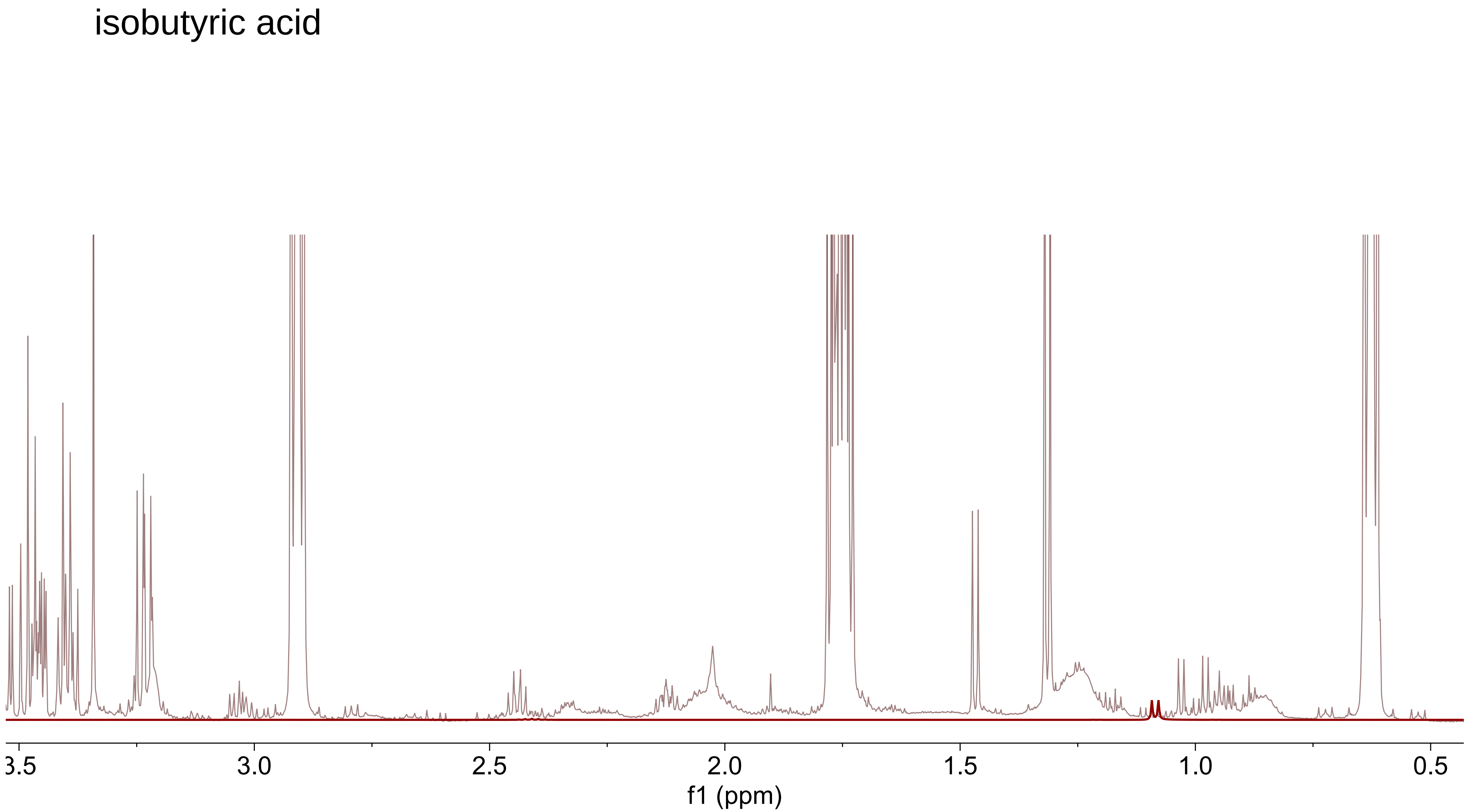
isobutyric acid
3.5
3.0
2.5
2.0
1.5
1.0
0.5
f1 (ppm)

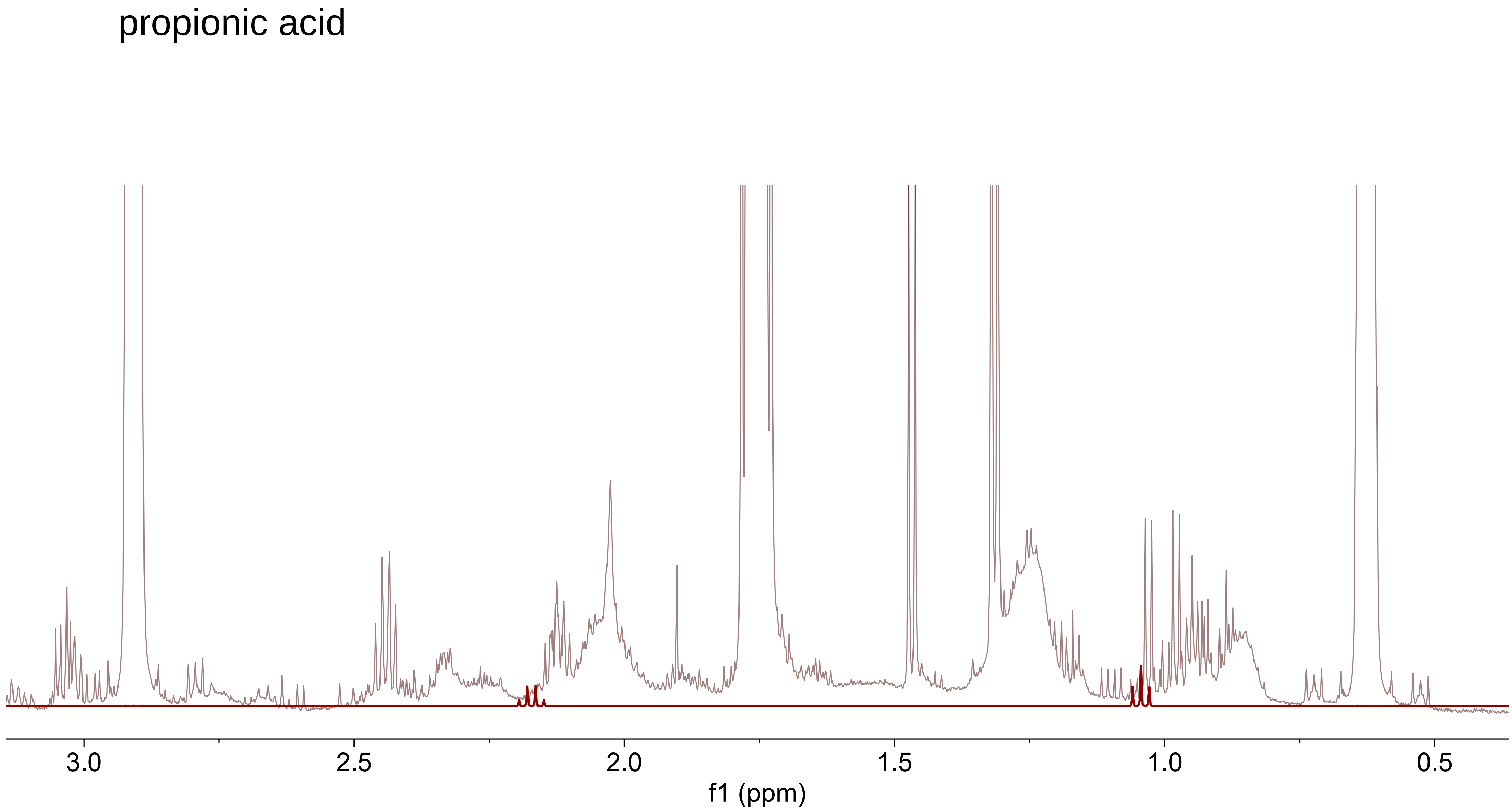
propionic acid
3.0
2.5
2.0
1.5
1.0
0.5
f1 (ppm)

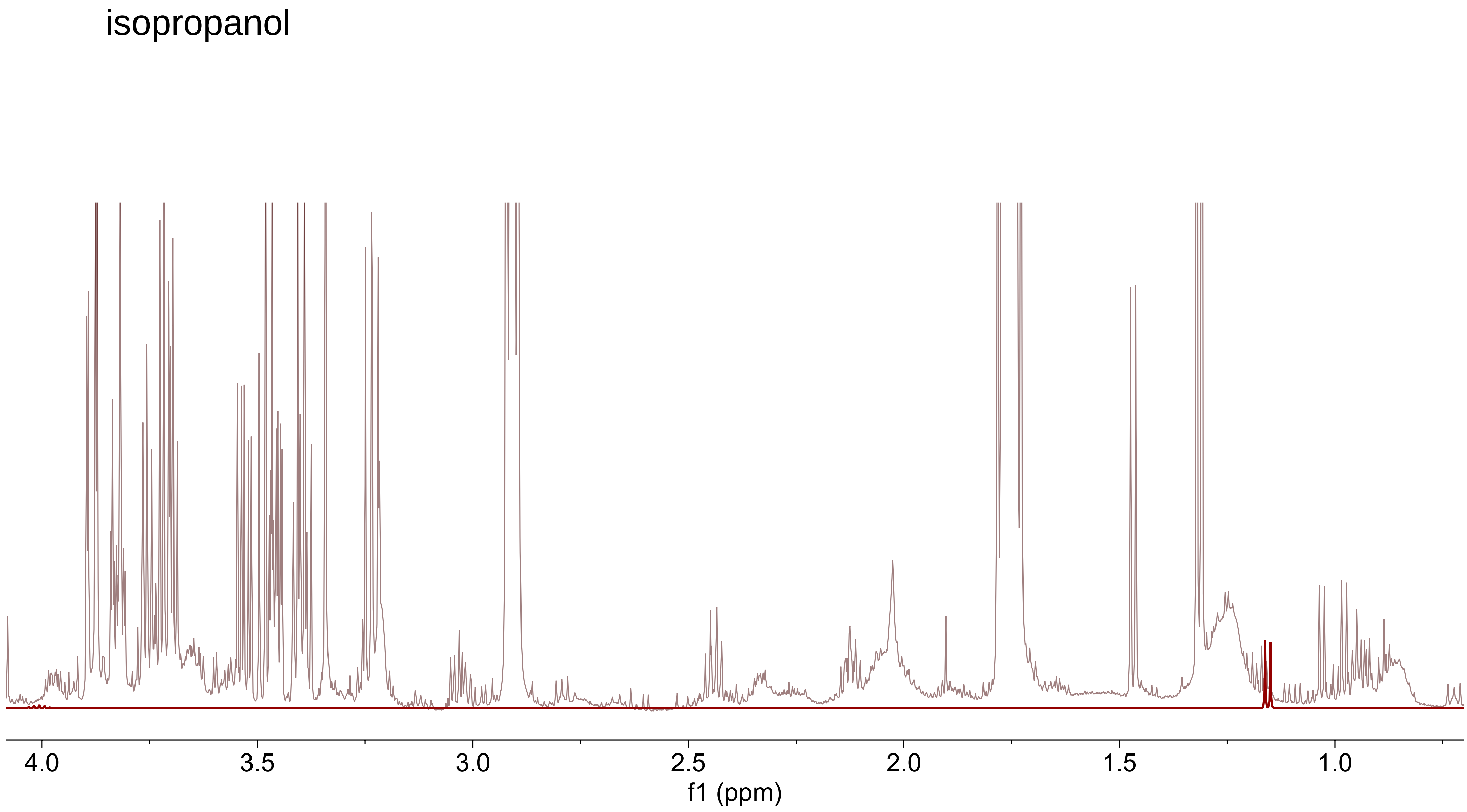

isopropanol
4.0
3.5
3.0
2.5
2.0
1.5
1.0
f1 (ppm)

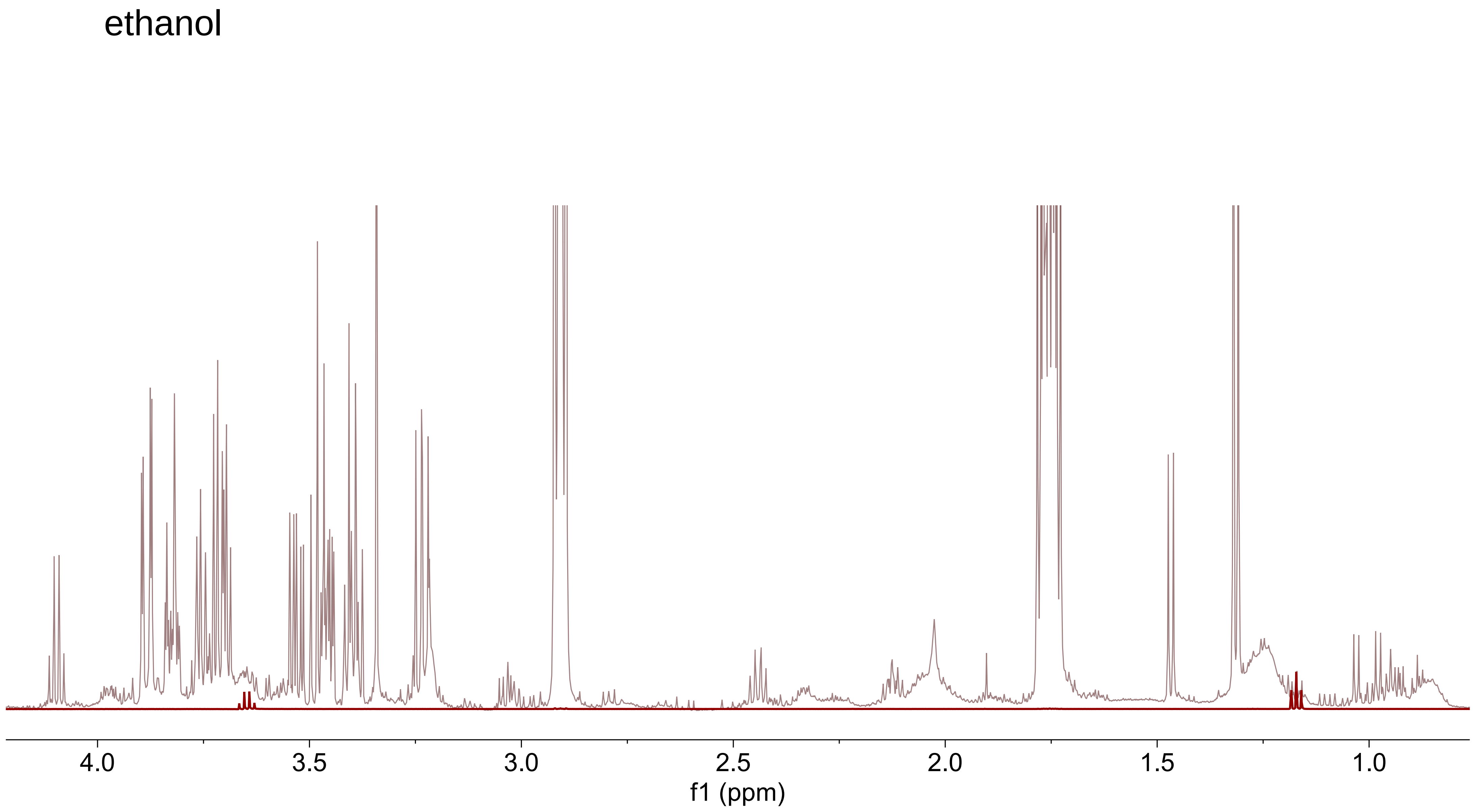
ethanol
4.0
3.5
3.0
2.5
2.0
1.5
1.0
f1 (ppm)

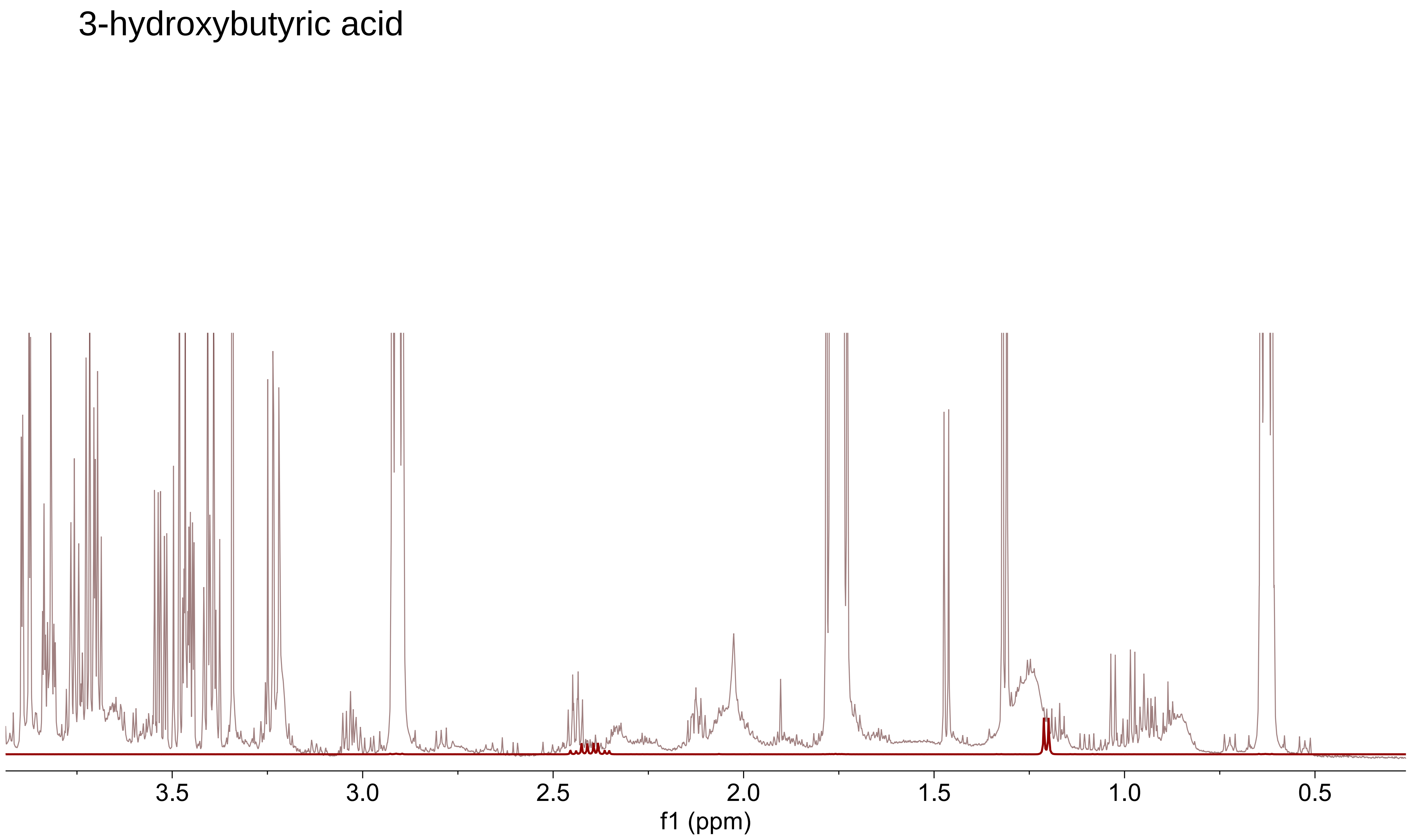
3-hydroxybutyric acid
3.5
3.0
2.5
2.0
1.5
1.0
0.5
f1 (ppm)

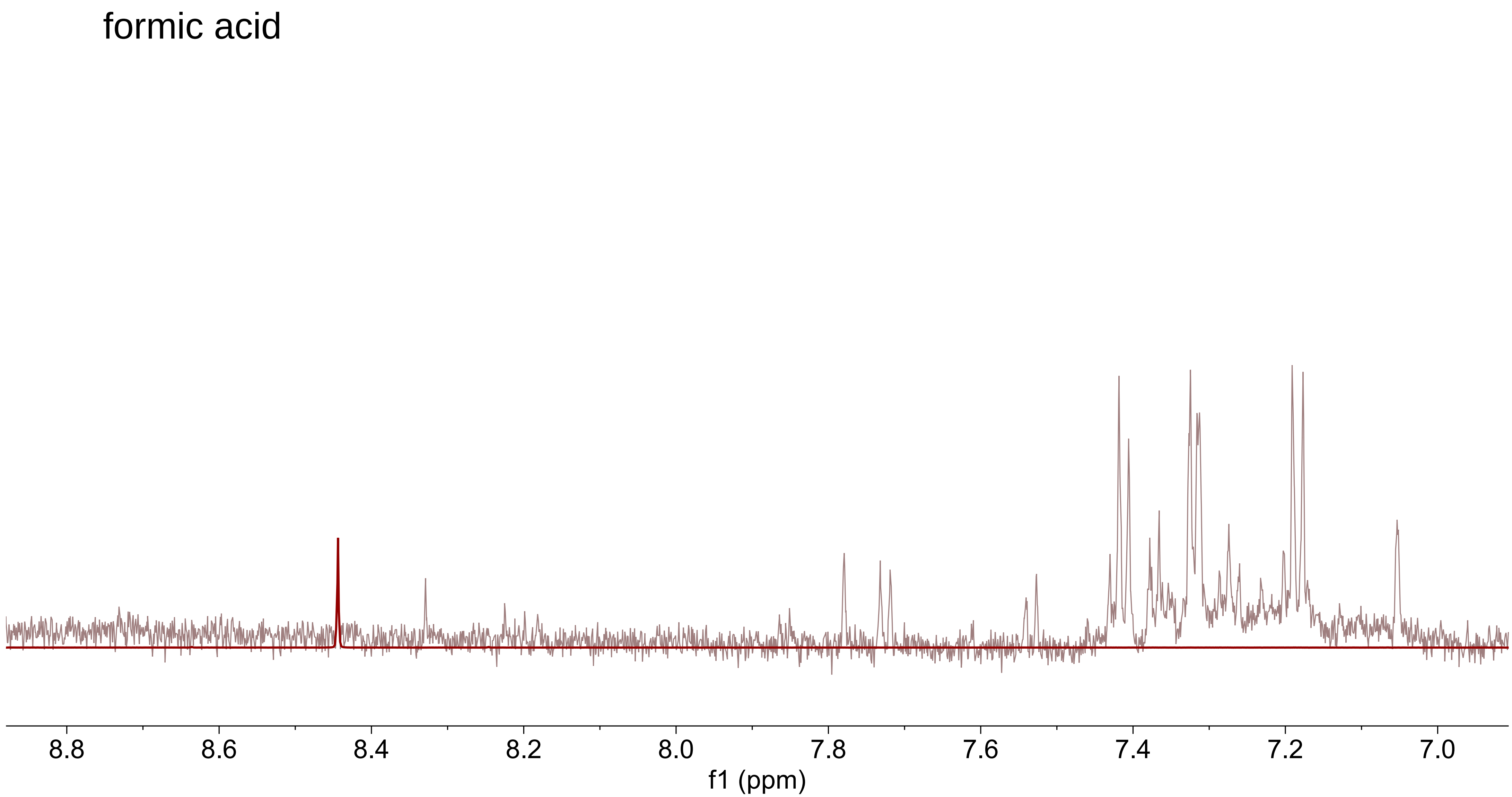
formic acid
8.8
8.6
8.4
8.2
8.0
7.8
7.6
7.4
7.2
7.0
f1 (ppm)

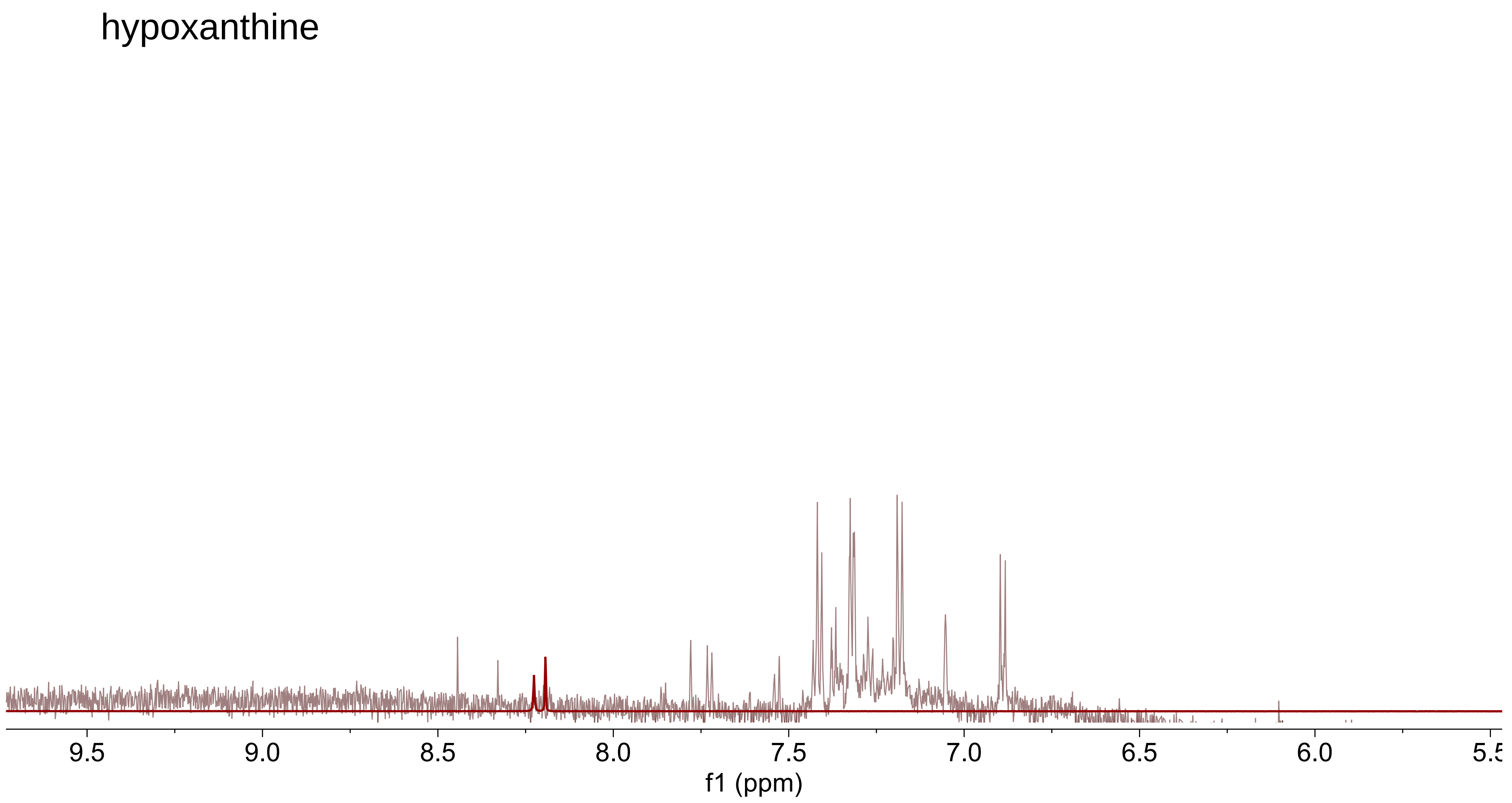
hypoxanthine
9.5
9.0
8.5
8.0
7.5
7.0
6.5
6.0
5.5
f1 (ppm)

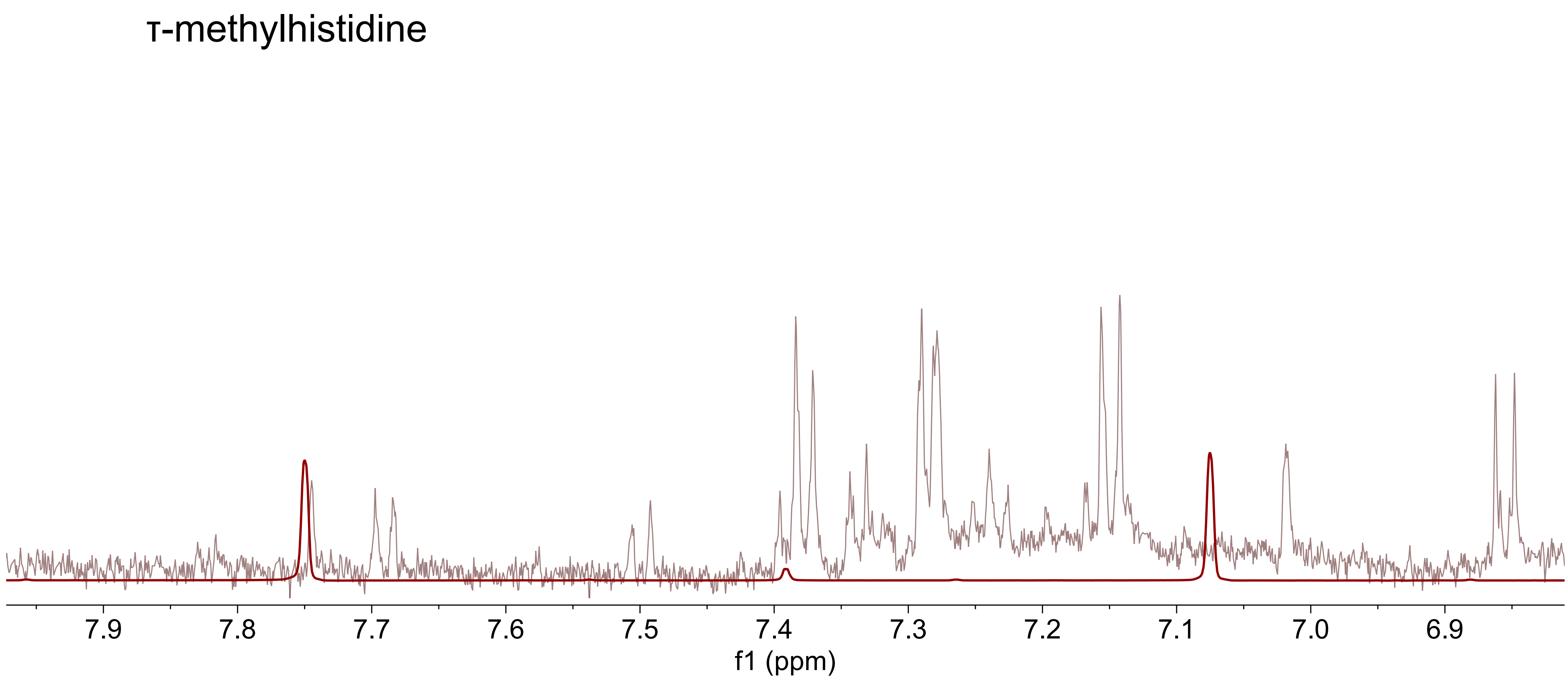
τ-methylhistidine
7.9
7.8
7.7
7.6
7.5
7.4
7.3
7.2
7.1
7.0
6.9
f1 (ppm)

histidine

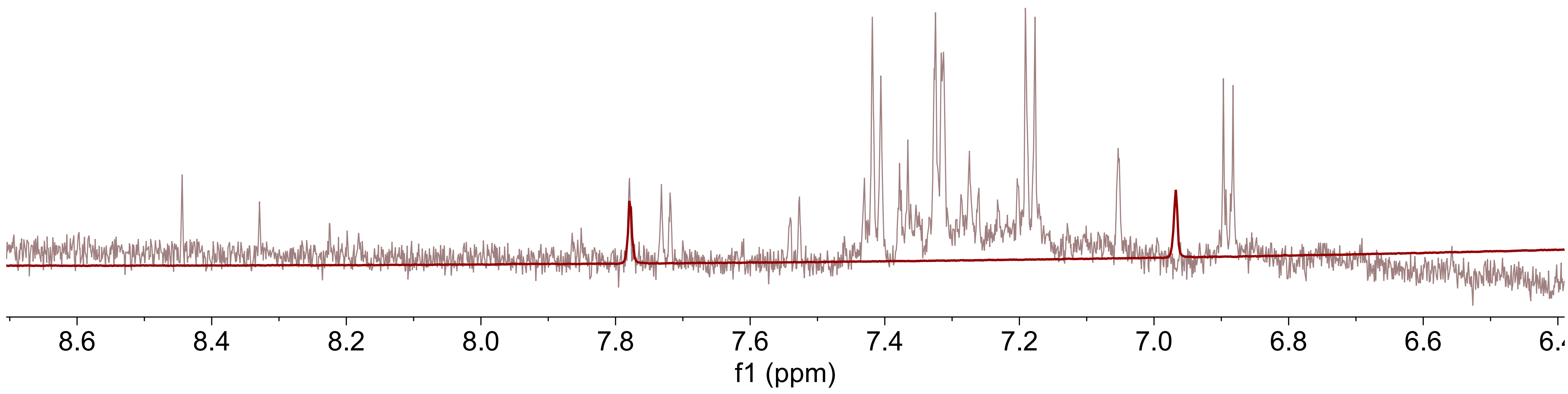

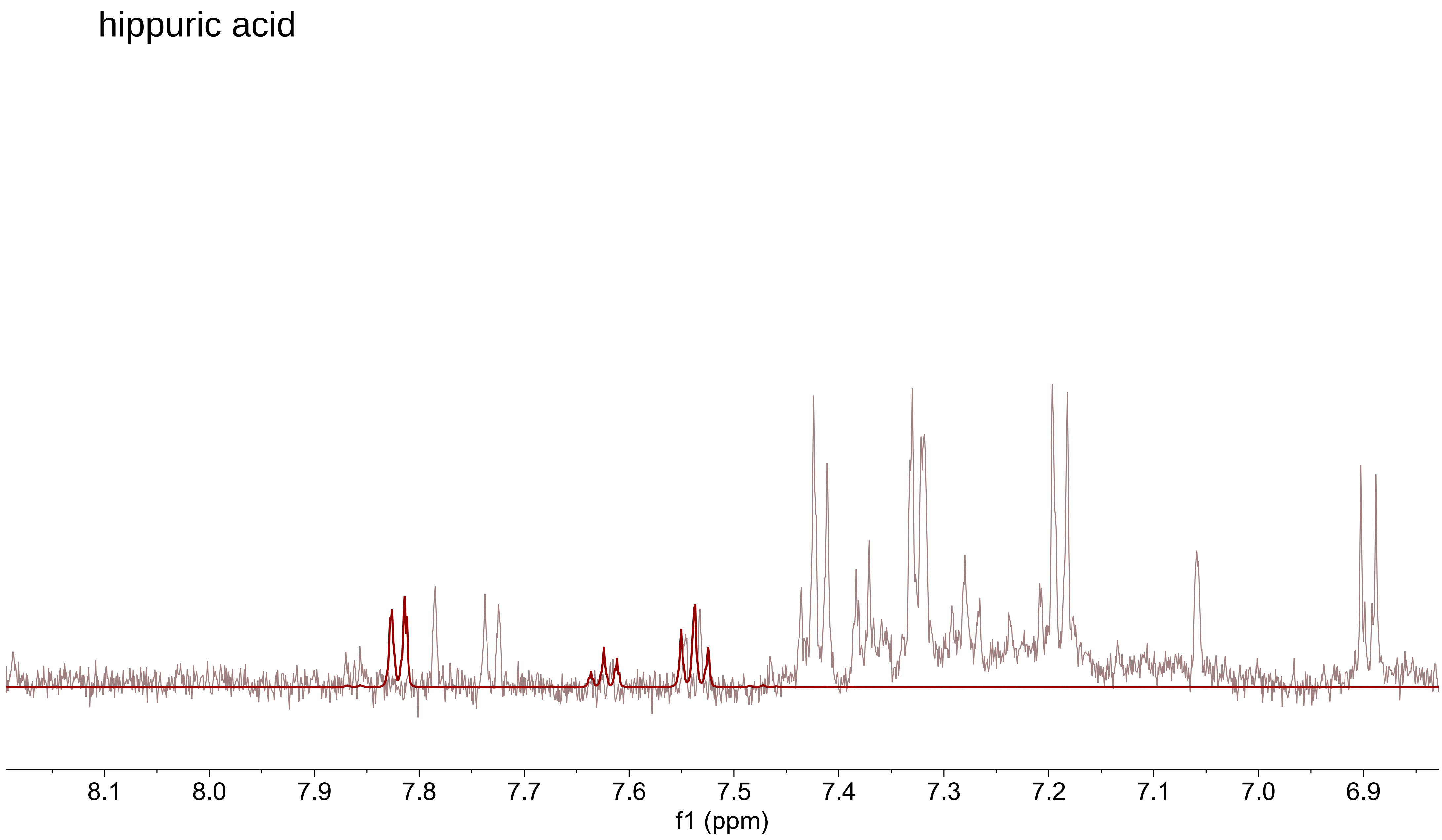
hippuric acid
8.1
8.0
7.9
7.8
7.7
7.6
7.5
7.4
7.3
7.2
7.1
7.0
6.9
f1 (ppm)

phenylalanine

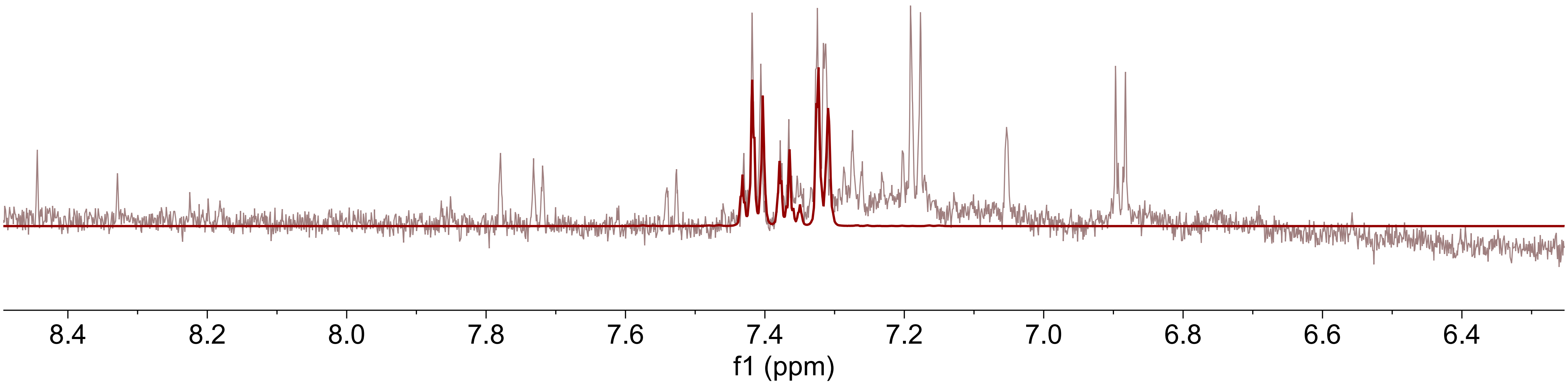

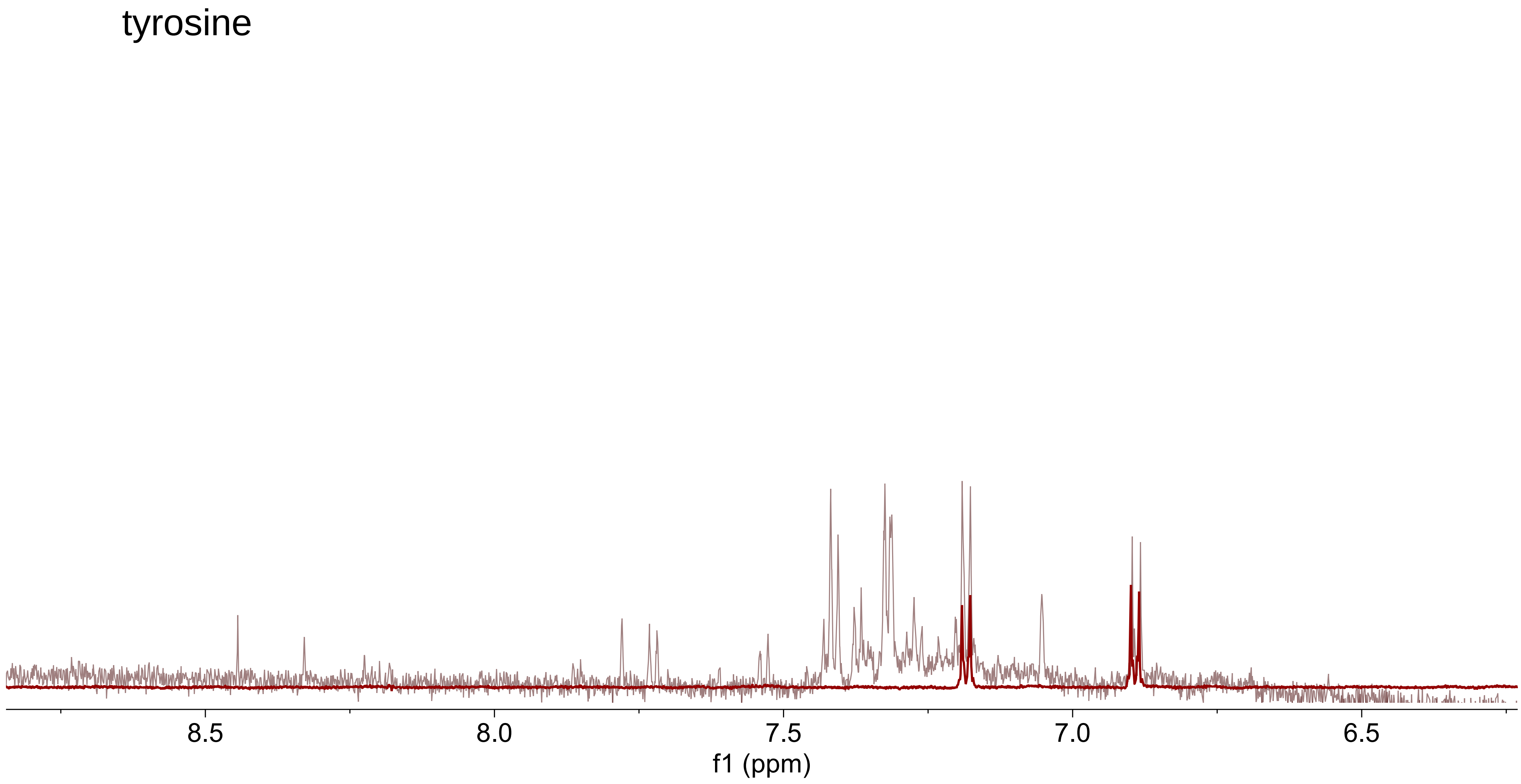
tyrosine
8.5
8.0
7.5
7.0
6.5
f1 (ppm)

π-methylhistidine

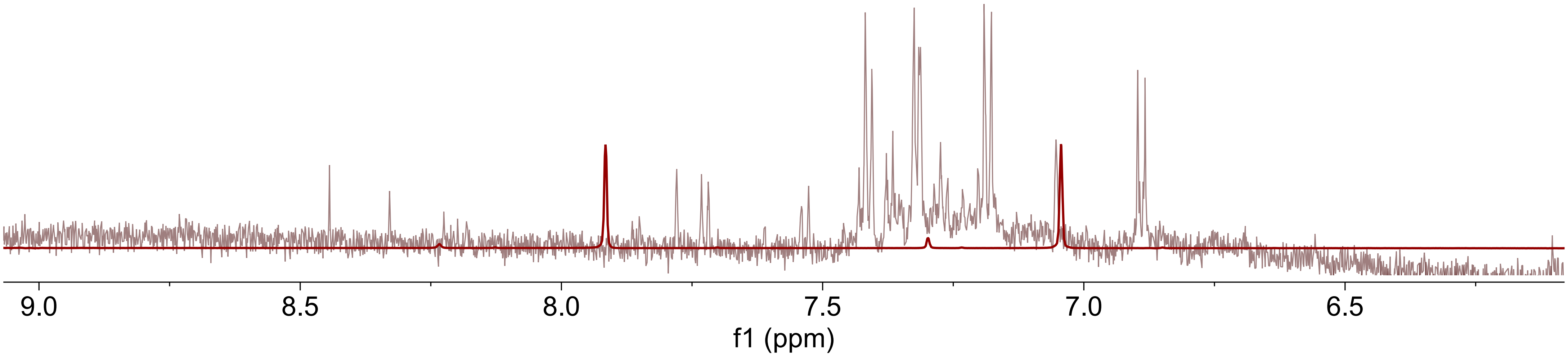

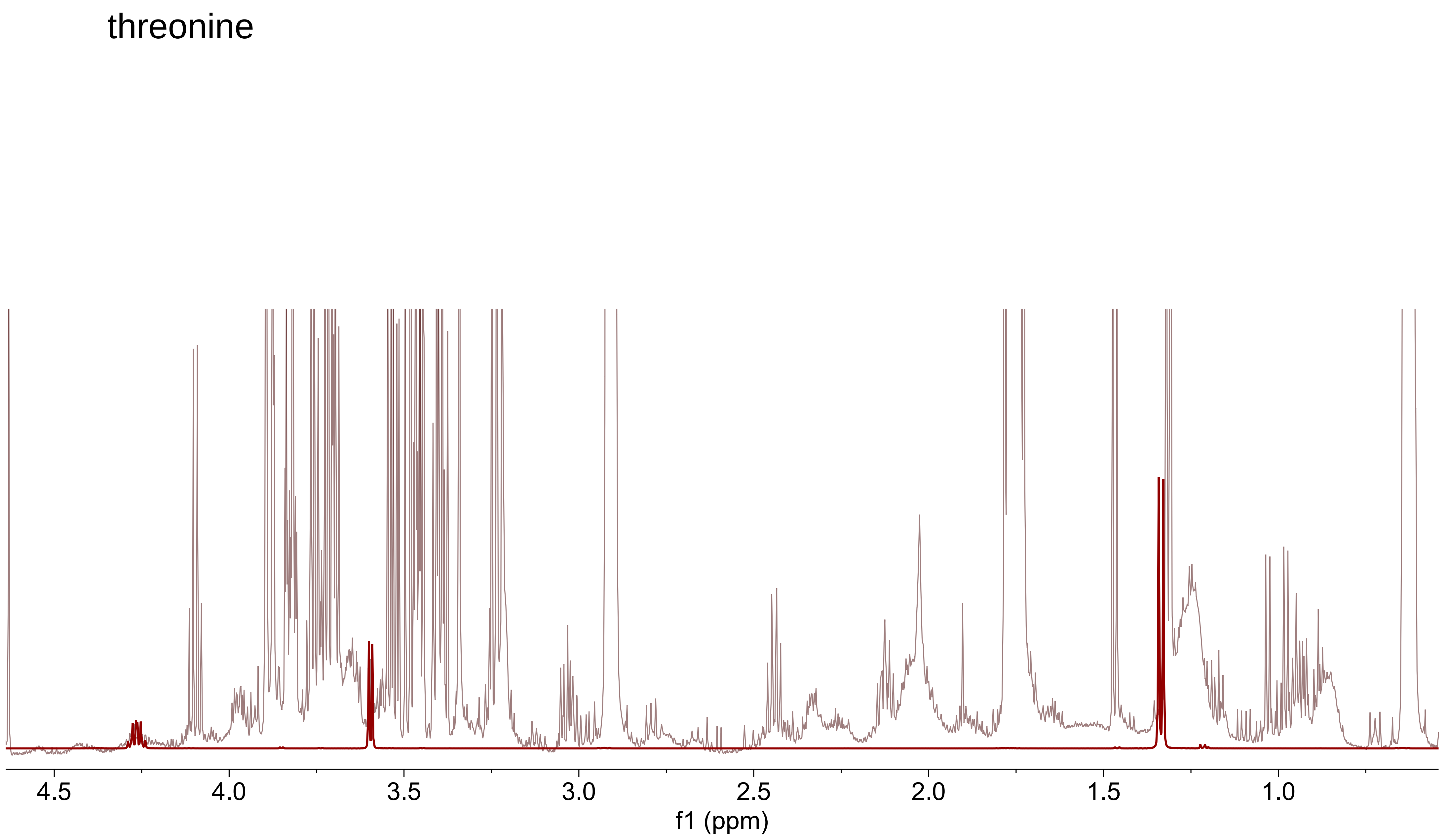
threonine
4.5
4.0
3.5
3.0
2.5
2.0
1.5
1.0
f1 (ppm)

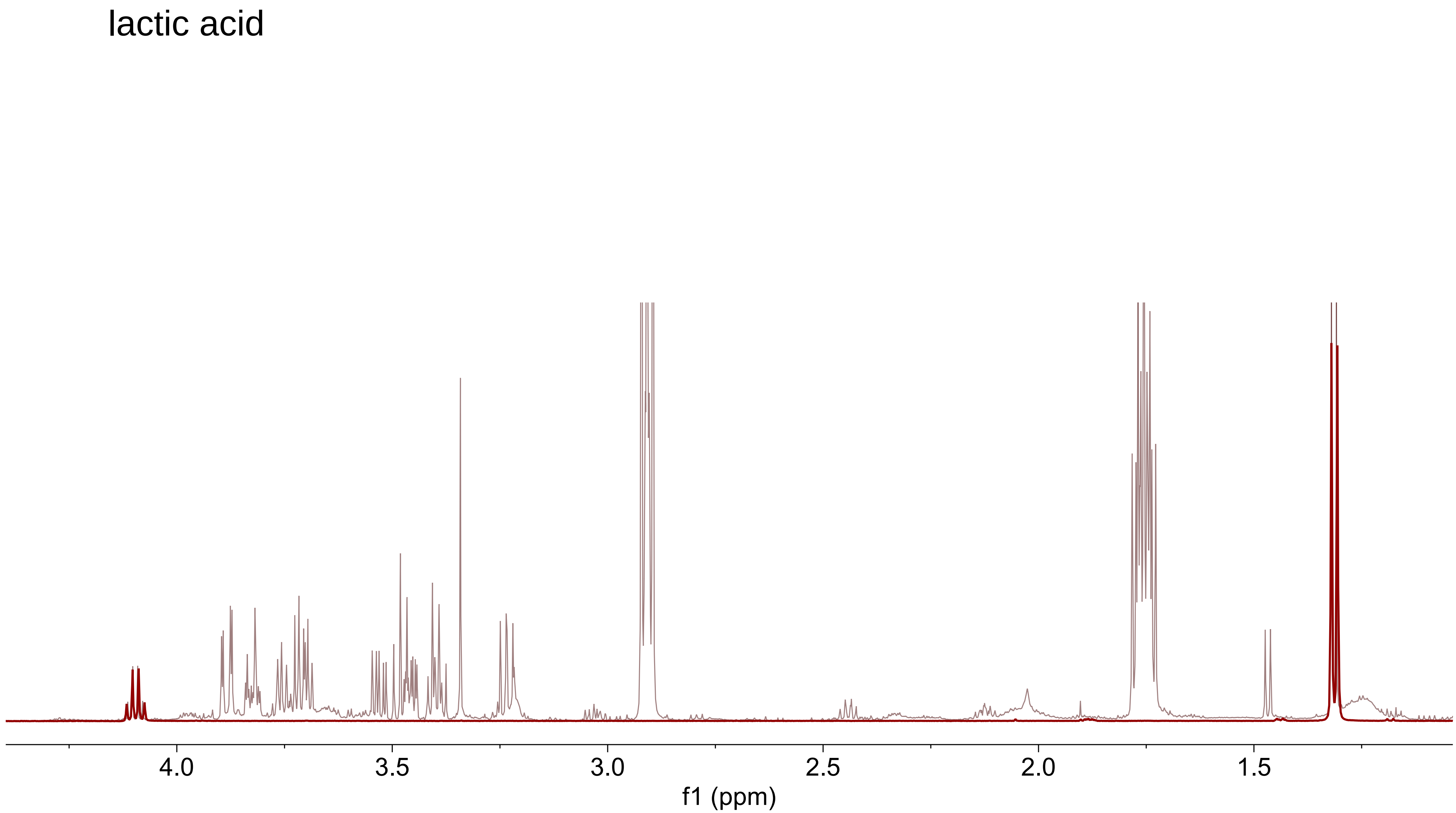
lactic acid
4.0
3.5
3.0
2.5
2.0
1.5
f1 (ppm)

3-hydroxyisovaleric acid

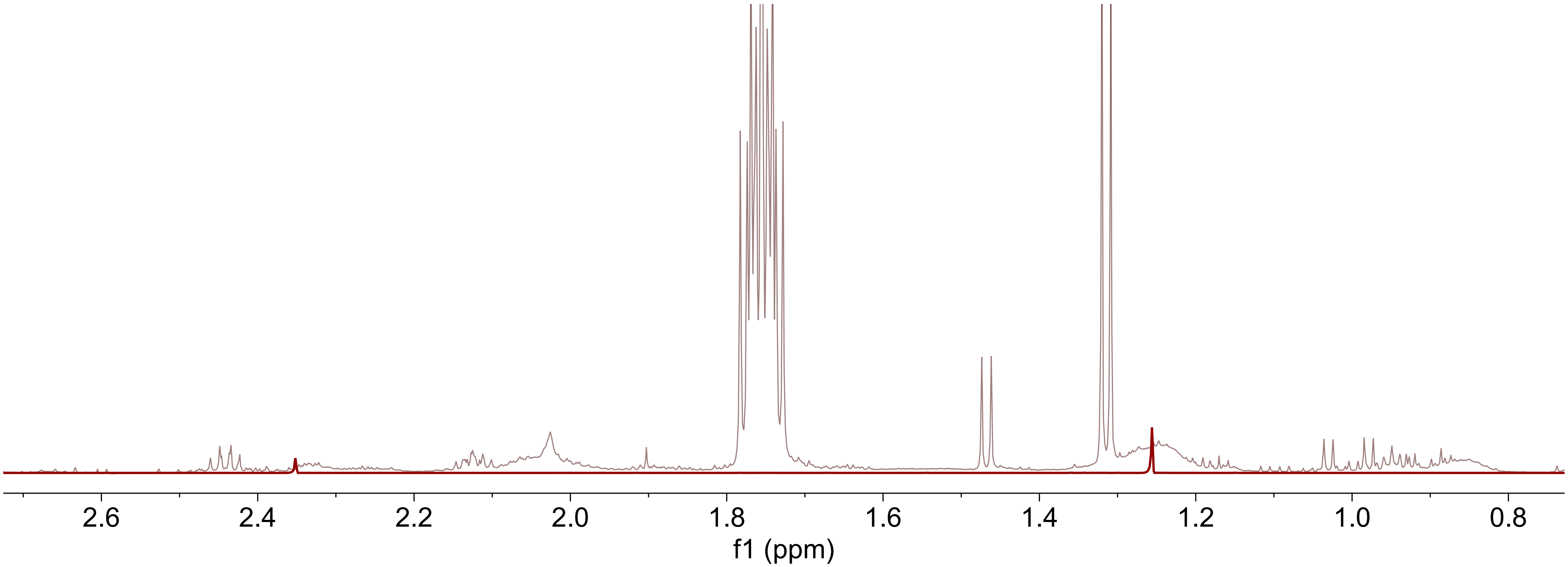

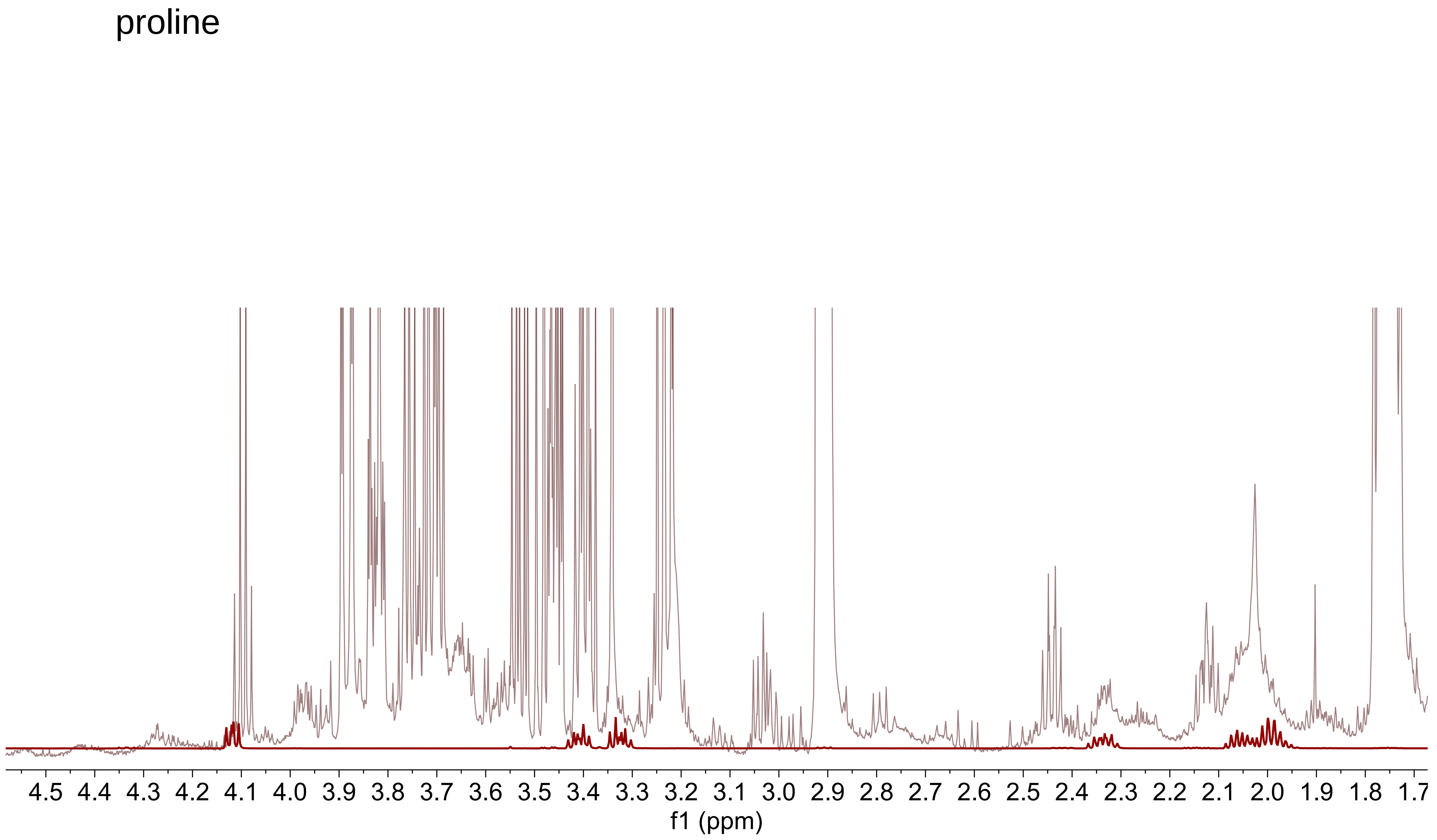
proline
4.5 4.4 4.3 4.2 4.1 4.0 3.9 3.8 3.7 3.6 3.5 3.4 3.3 3.2 3.1 3.0 2.9 2.8 2.7 2.6 2.5 2.4 2.3 2.2 2.1 2.0 1.9 1.8 1.7
f1 (ppm)

pyroglutamic acid

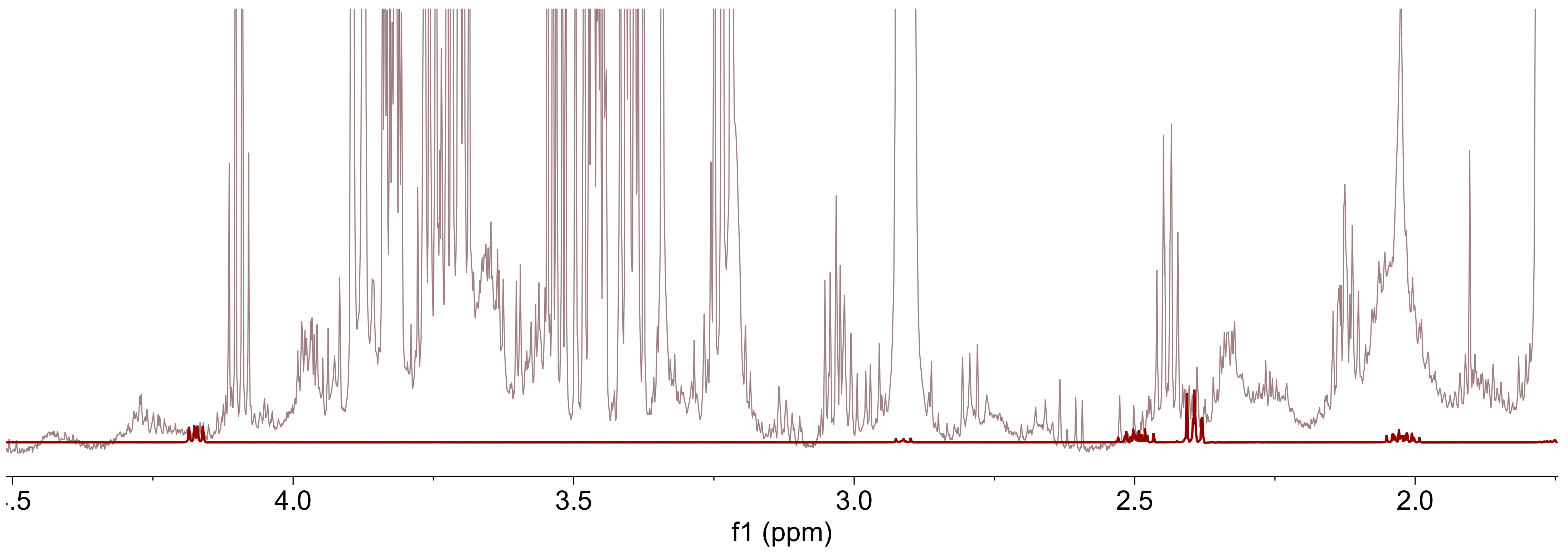

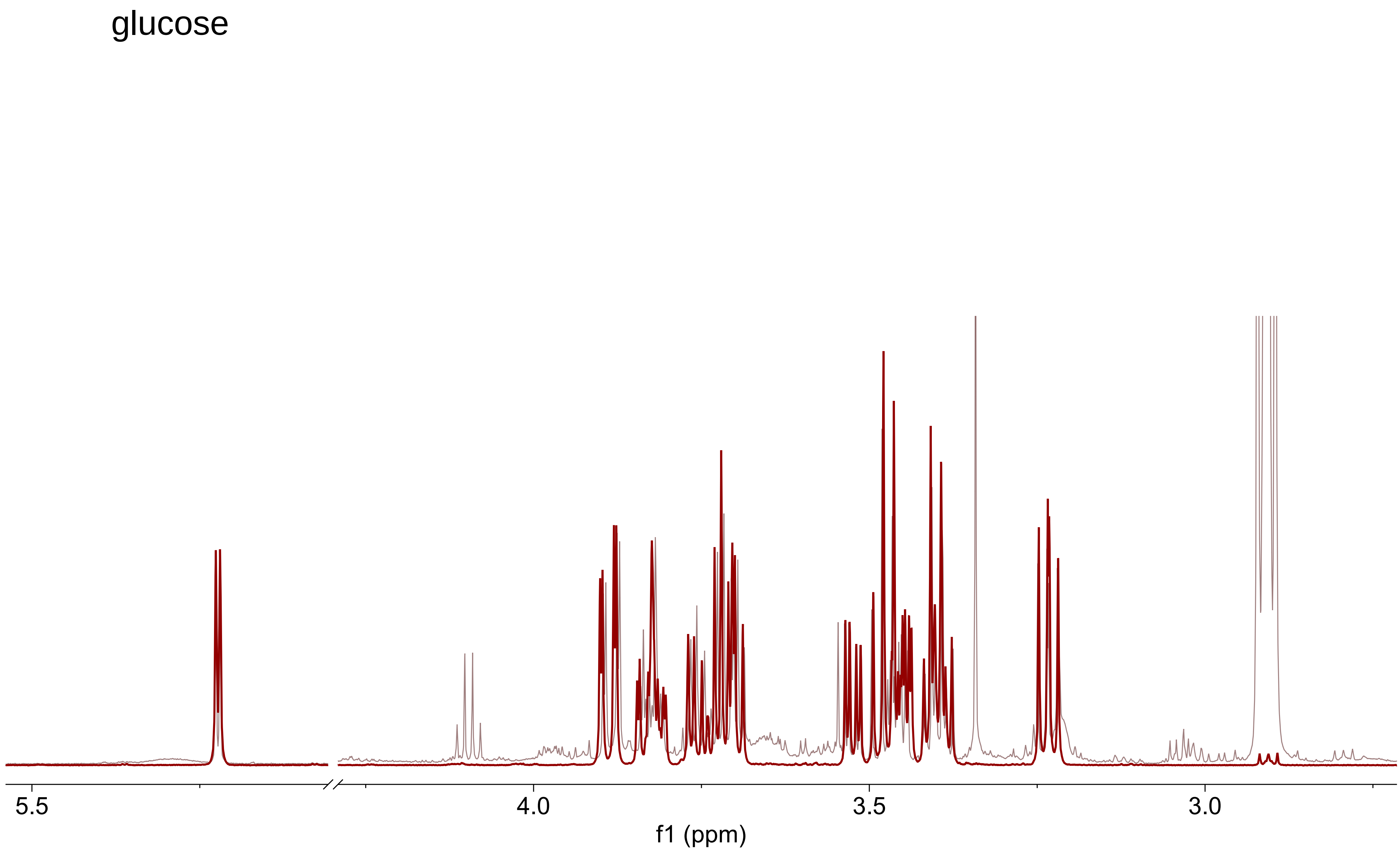

glucose
5.5
4.0
3.5
3.0
f1 (ppm)

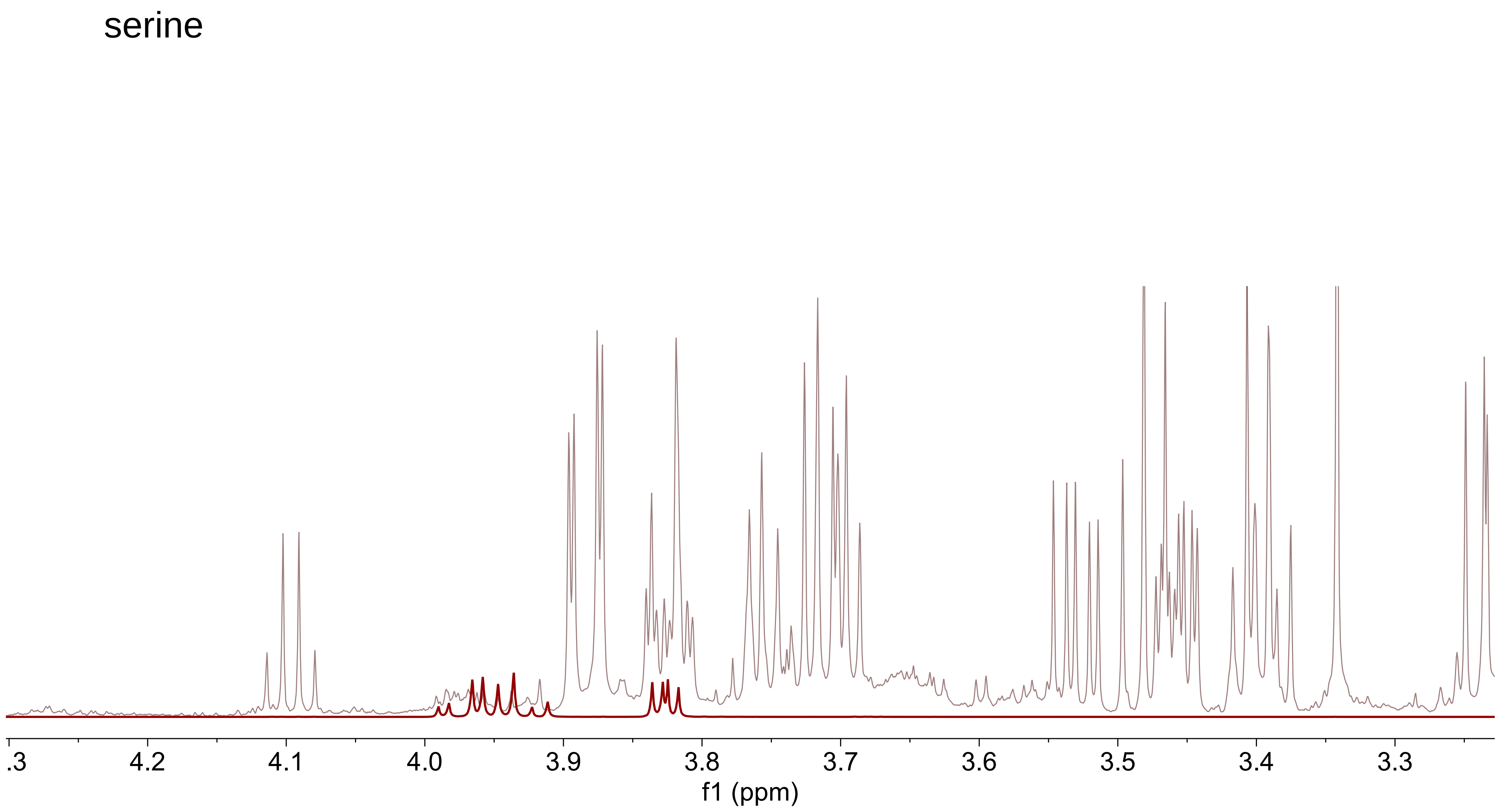

serine
.3
4.2
4.1
4.0
3.9
3.8
3.7
3.6
3.5
3.4
3.3
f1 (ppm)

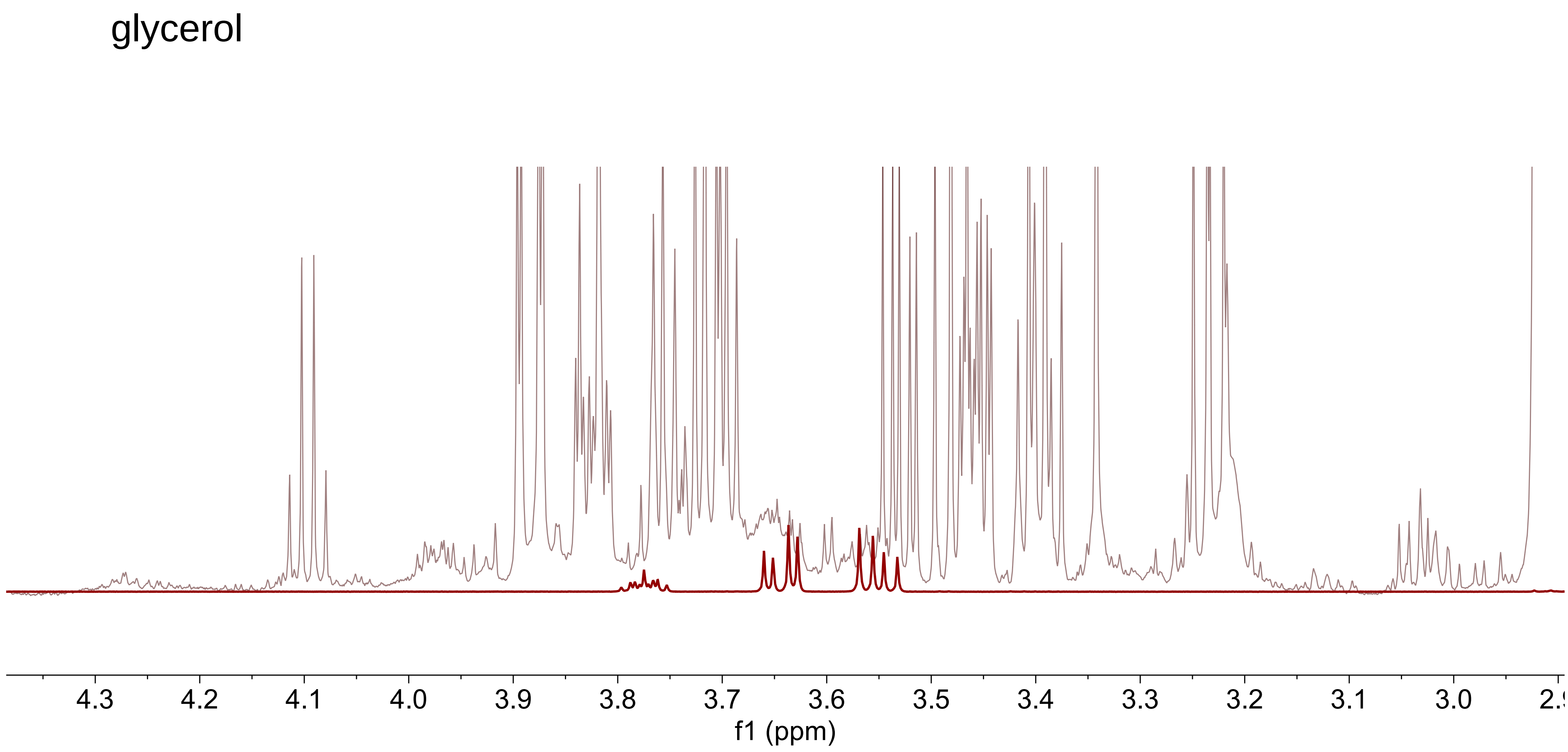
glycerol
4.3
4.2
4.1
4.0
3.9
3.8
3.7
3.6
3.5
3.4
3.3
3.2
3.1
3.0
f1 (ppm)

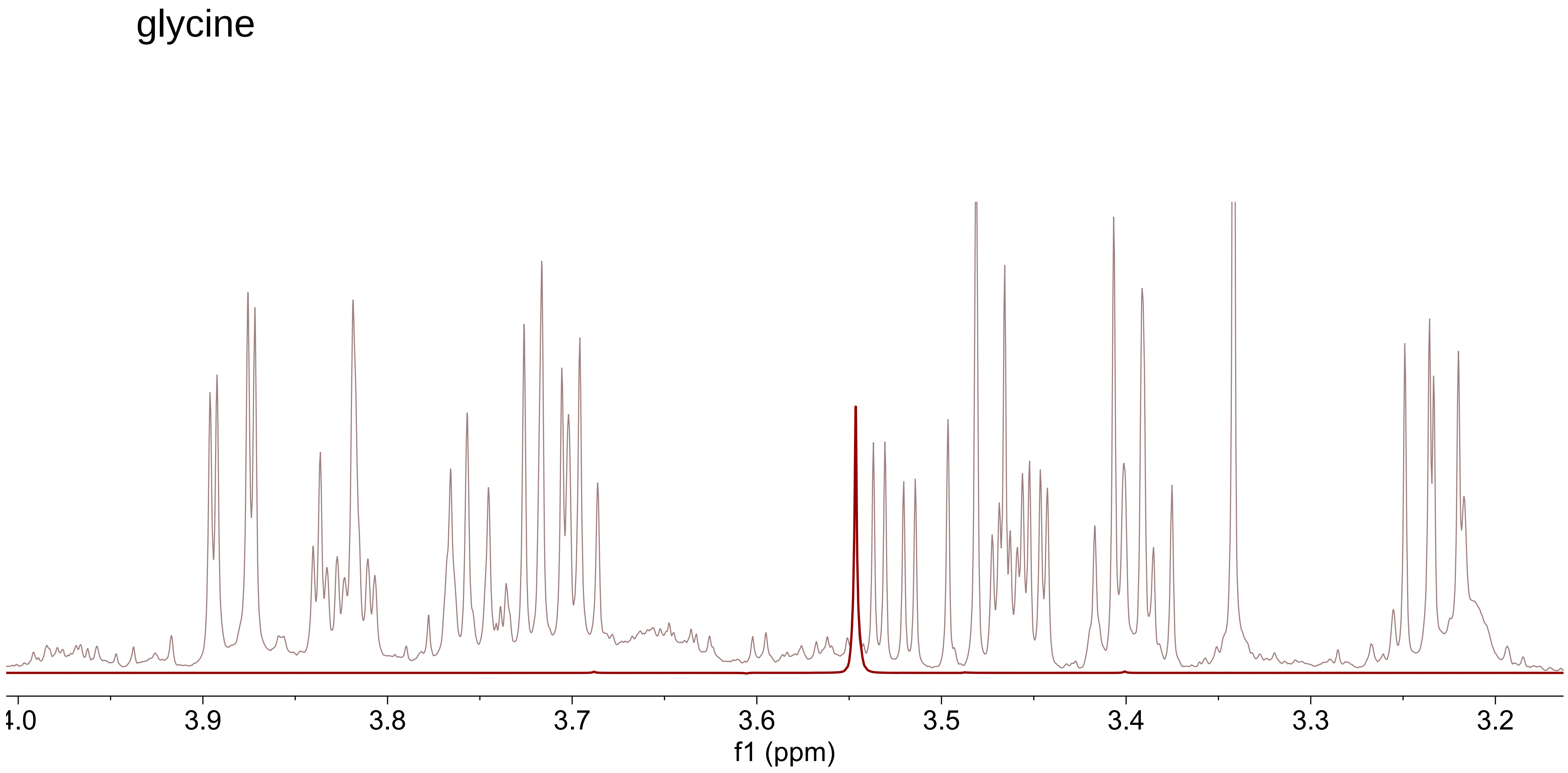
glycine
4.0
3.9
3.8
3.7
3.6
3.5
3.4
3.3
3.2
f1 (ppm)

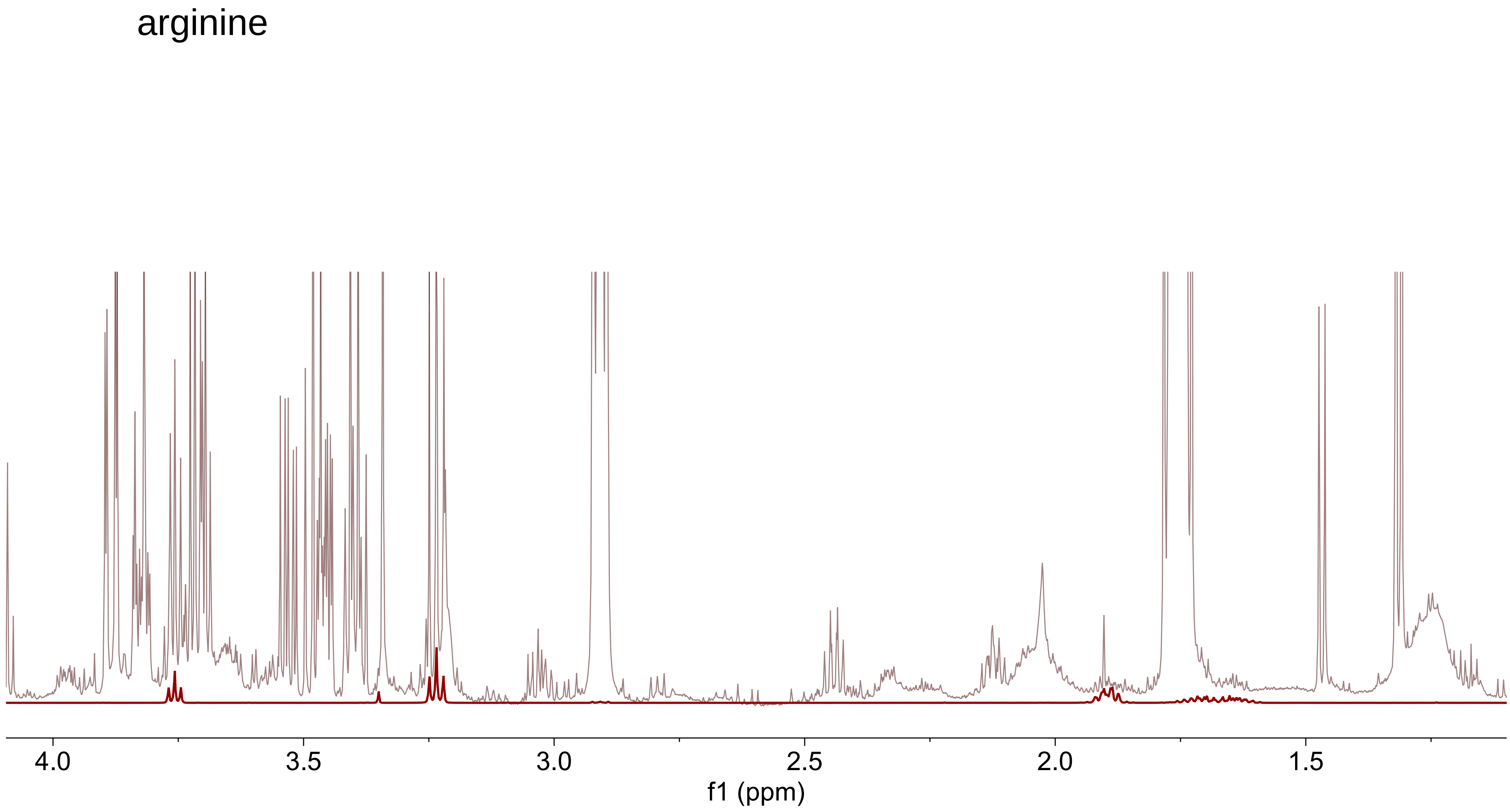

arginine
4.0
3.5
3.0
2.5
2.0
1.5
f1 (ppm)

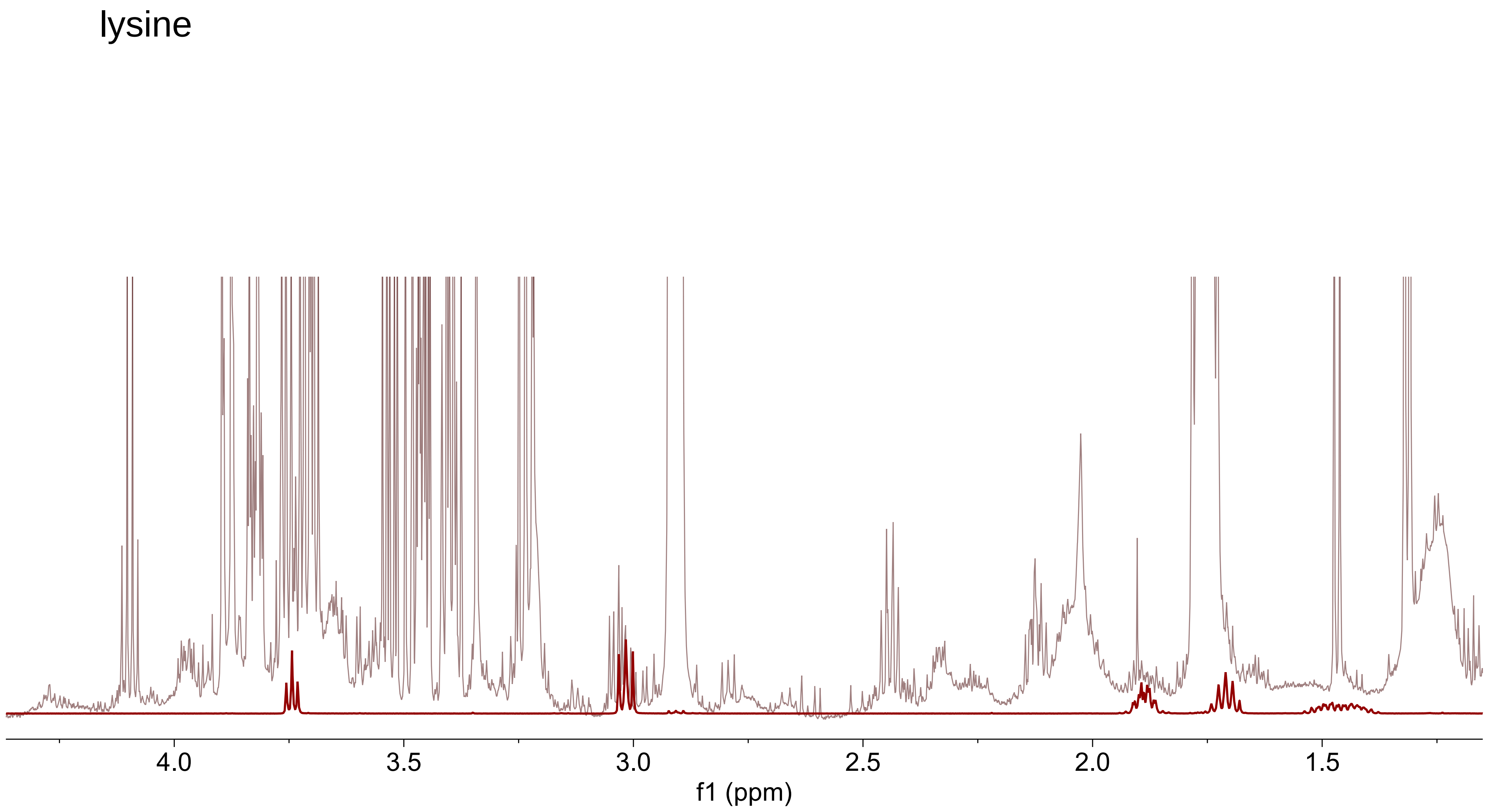
lysine
4.0
3.5
3.0
2.5
2.0
1.5
f1 (ppm)

2-oxoglutaric acid

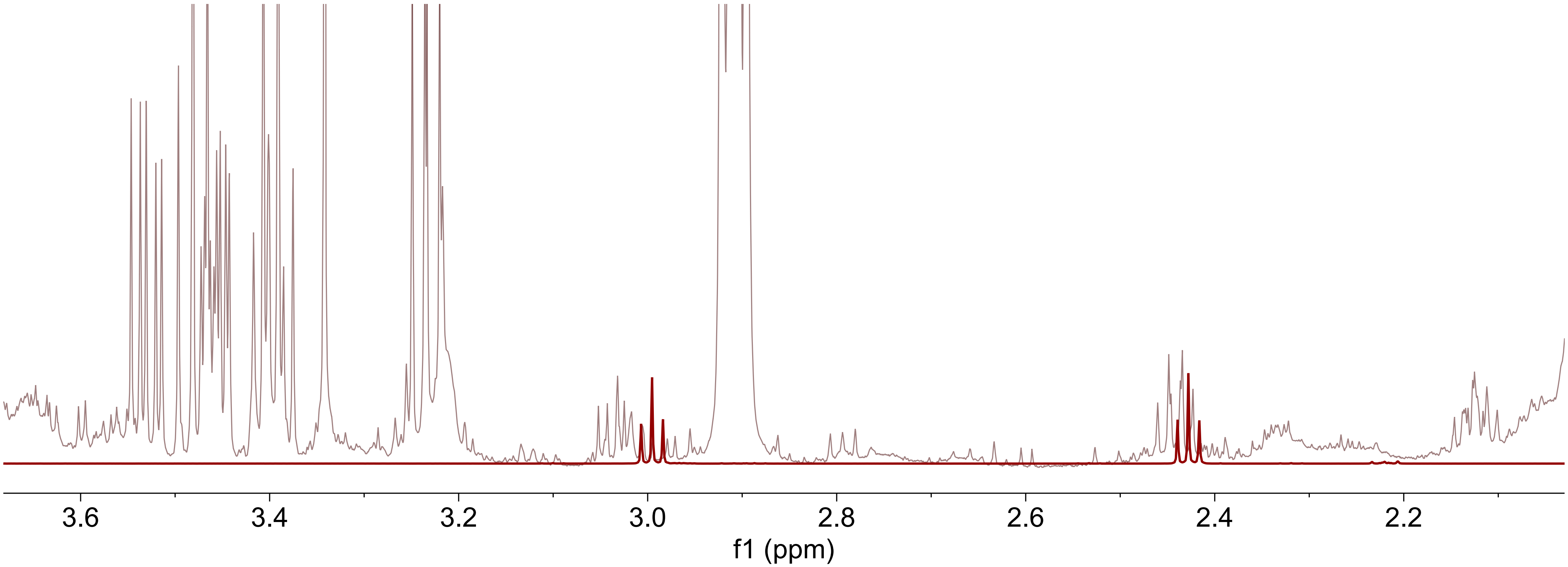

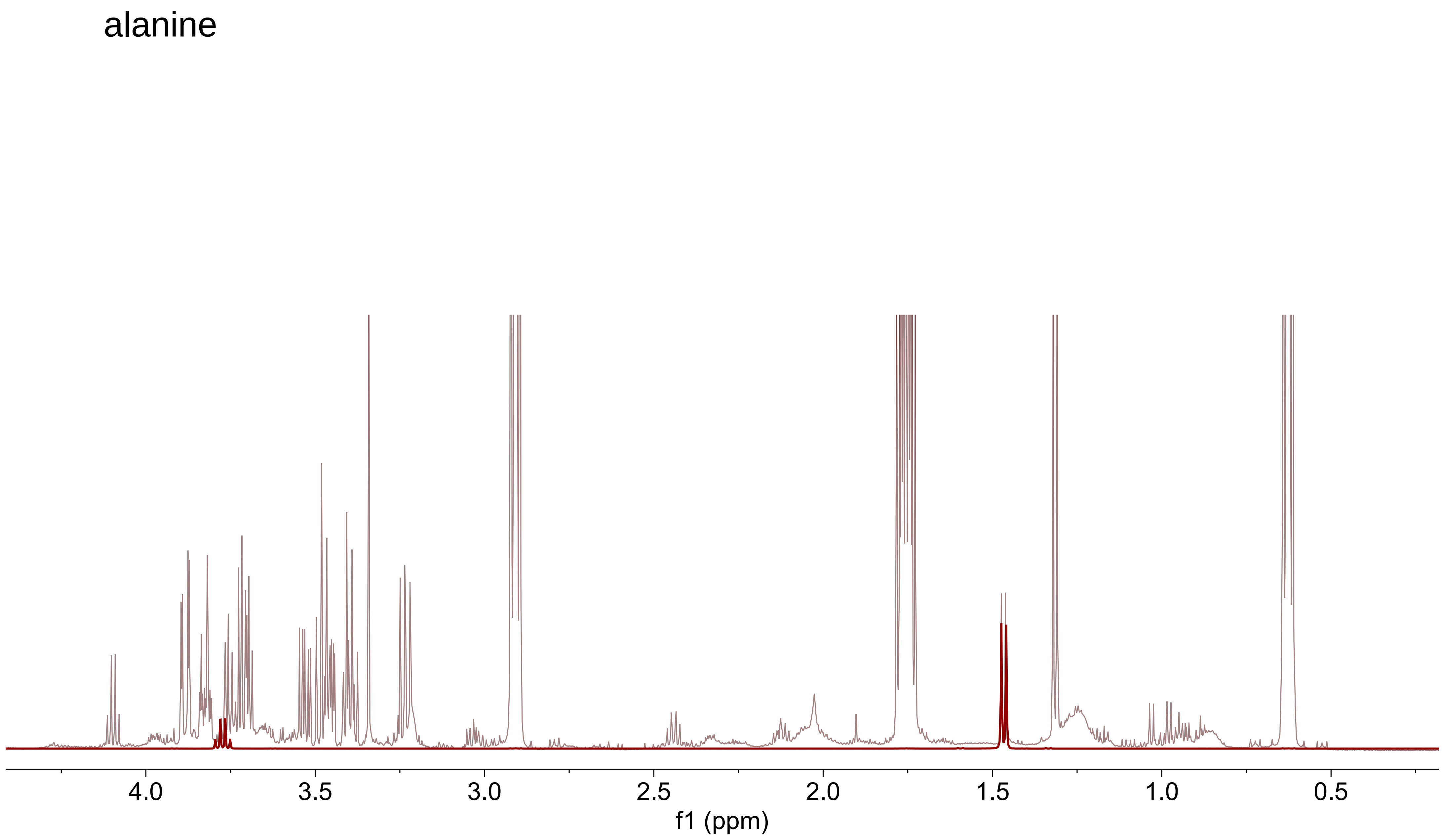
alanine
4.0
3.5
3.0
2.5
2.0
1.5
1.0
0.5
f1 (ppm)

acetic acid

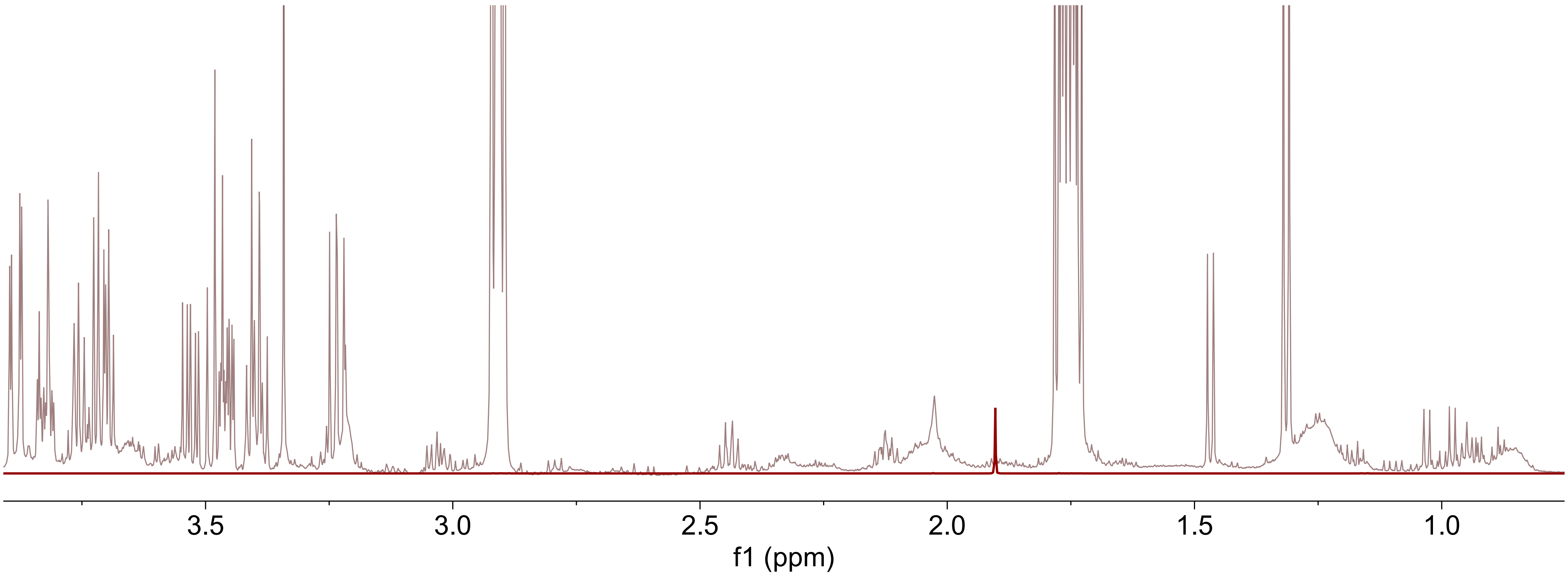

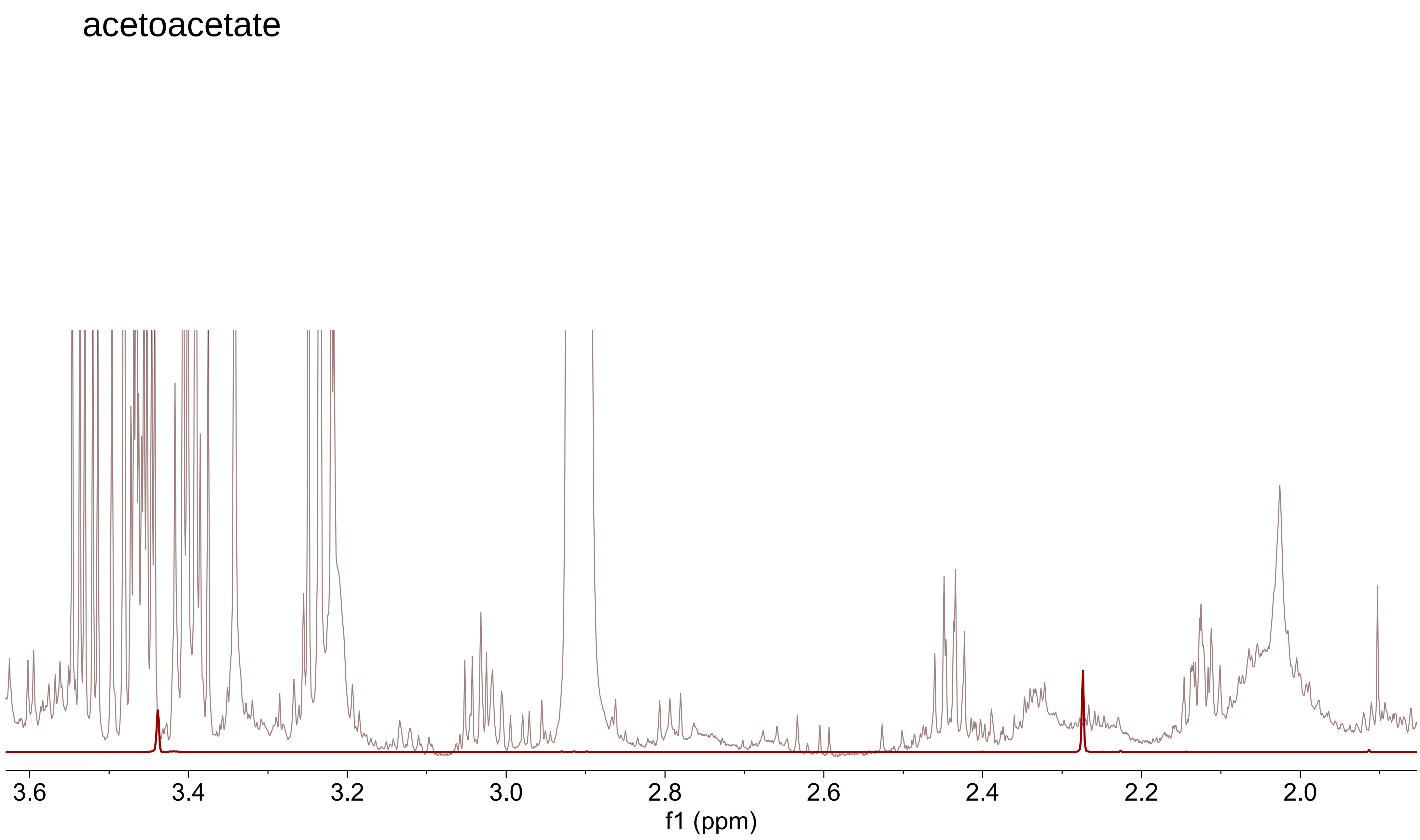
acetoacetate
3.6
3.4
3.2
3.0
2.8
2.6
2.4
2.2
2.0
f1 (ppm)

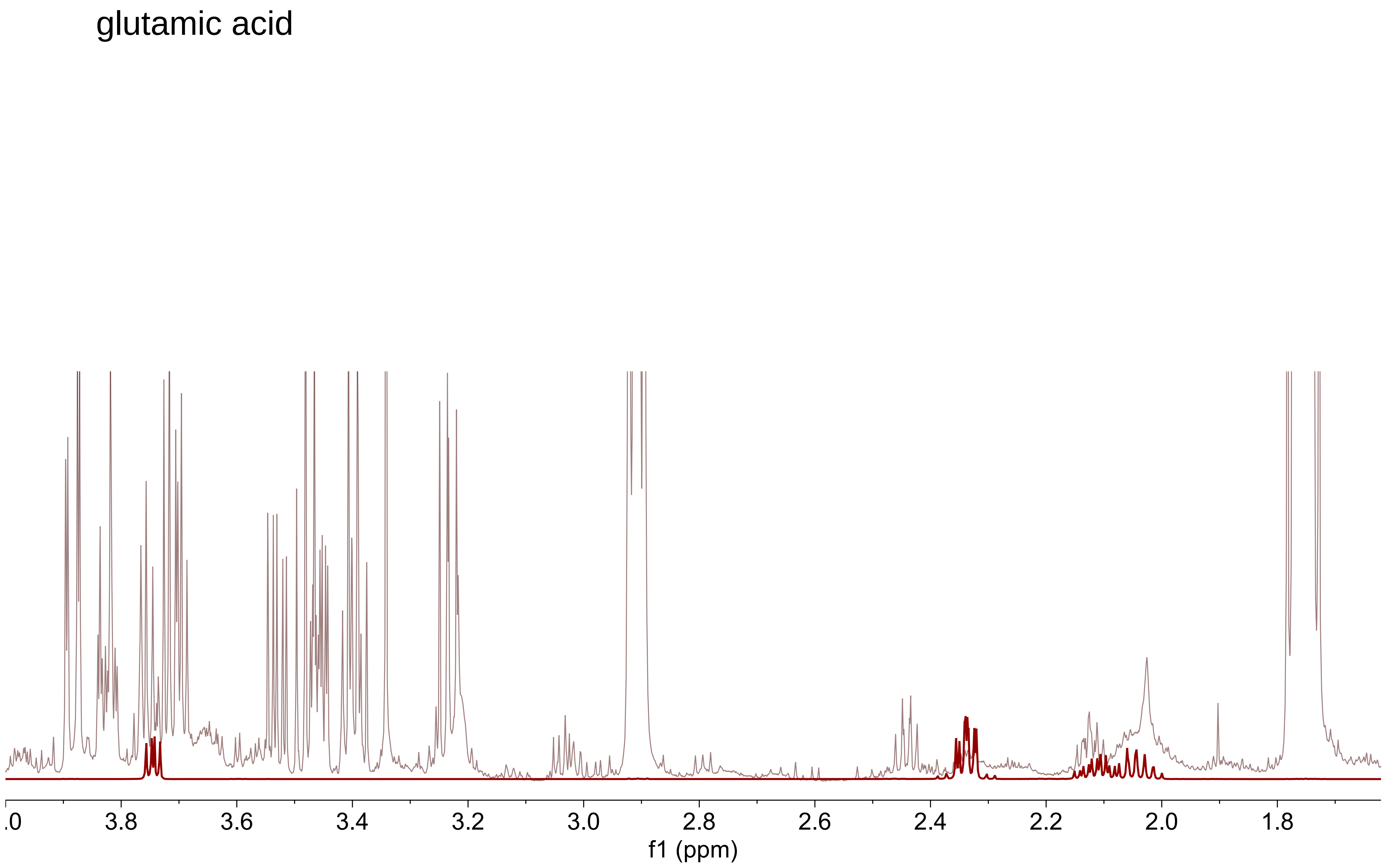
glutamic acid
.0
3.8
3.6
3.4
3.2
3.0
2.8
2.6
2.4
2.2
2.0
1.8
f1 (ppm)

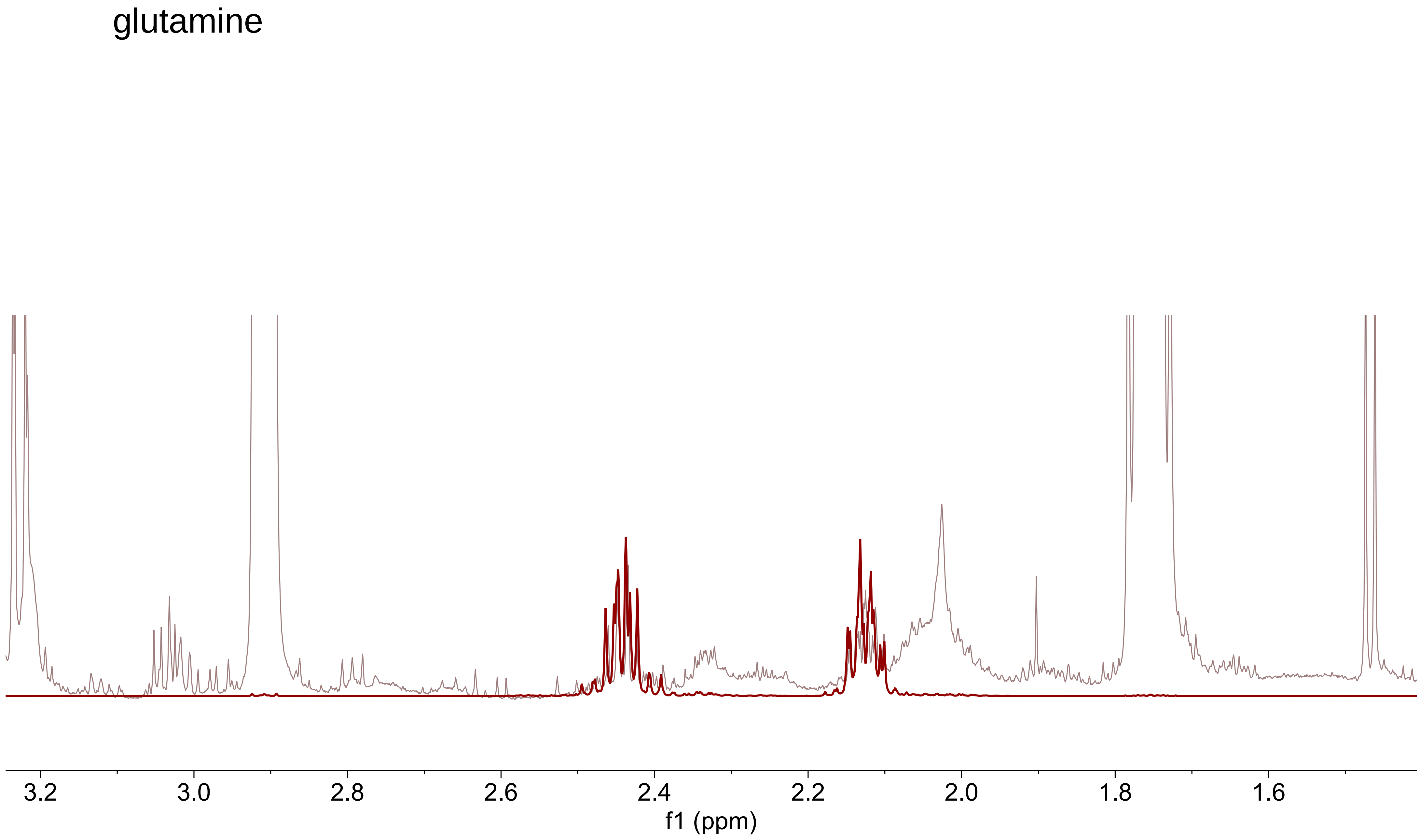
glutamine
3.2
3.0
2.8
2.6
2.4
2.2
2.0
1.8
1.6
f1 (ppm)

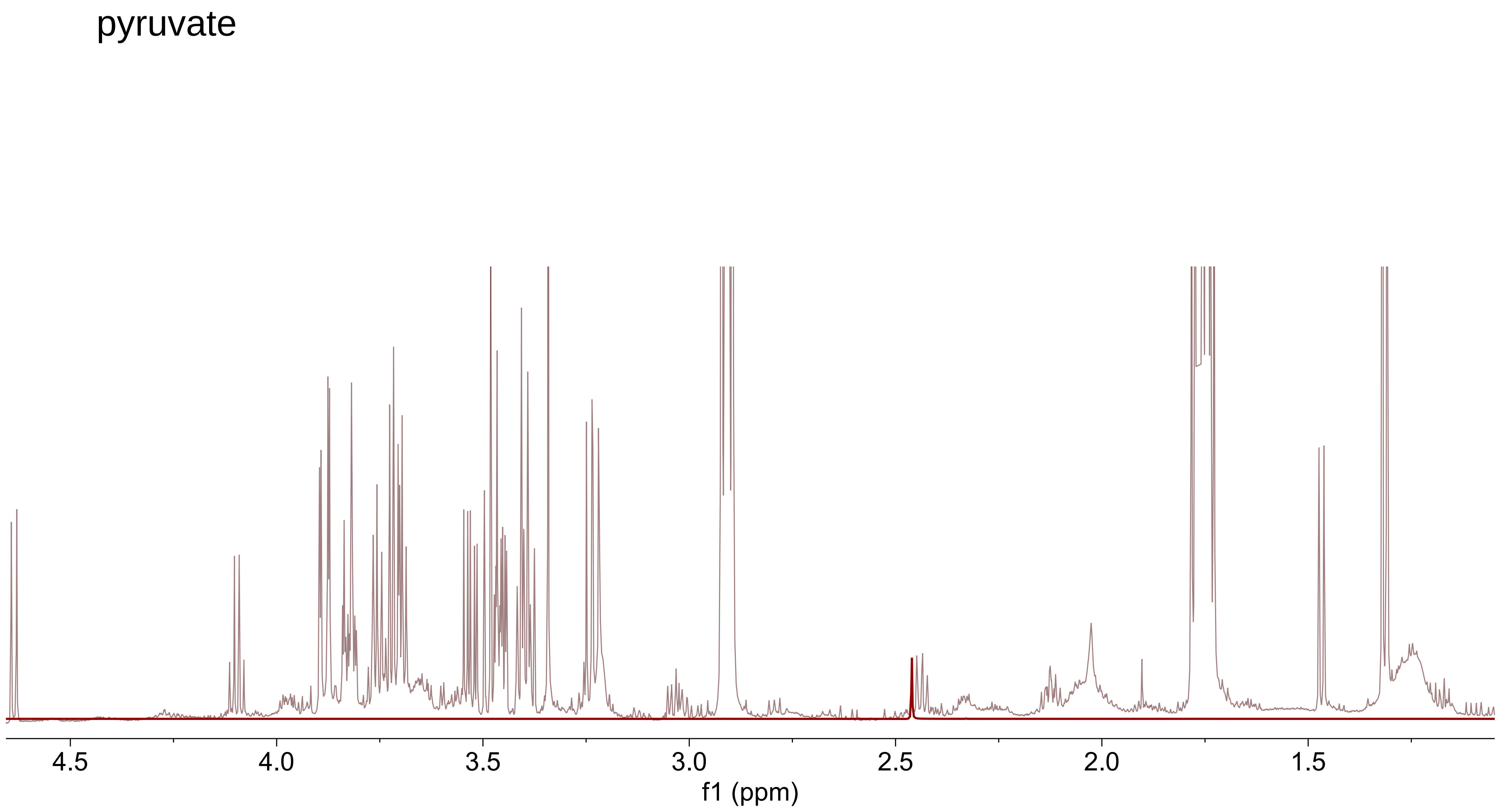
pyruvate
4.5
4.0
3.5
3.0
2.5
2.0
1.5
f1 (ppm)

N-acetylglycine

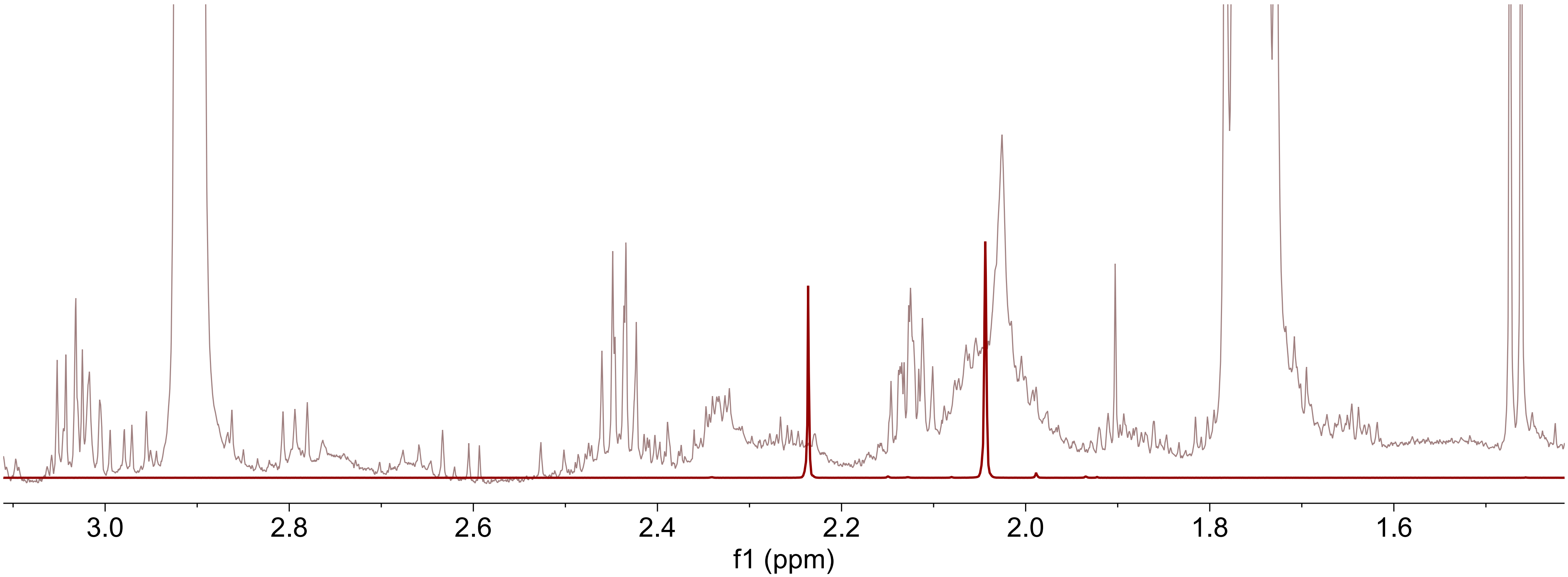

citric acid

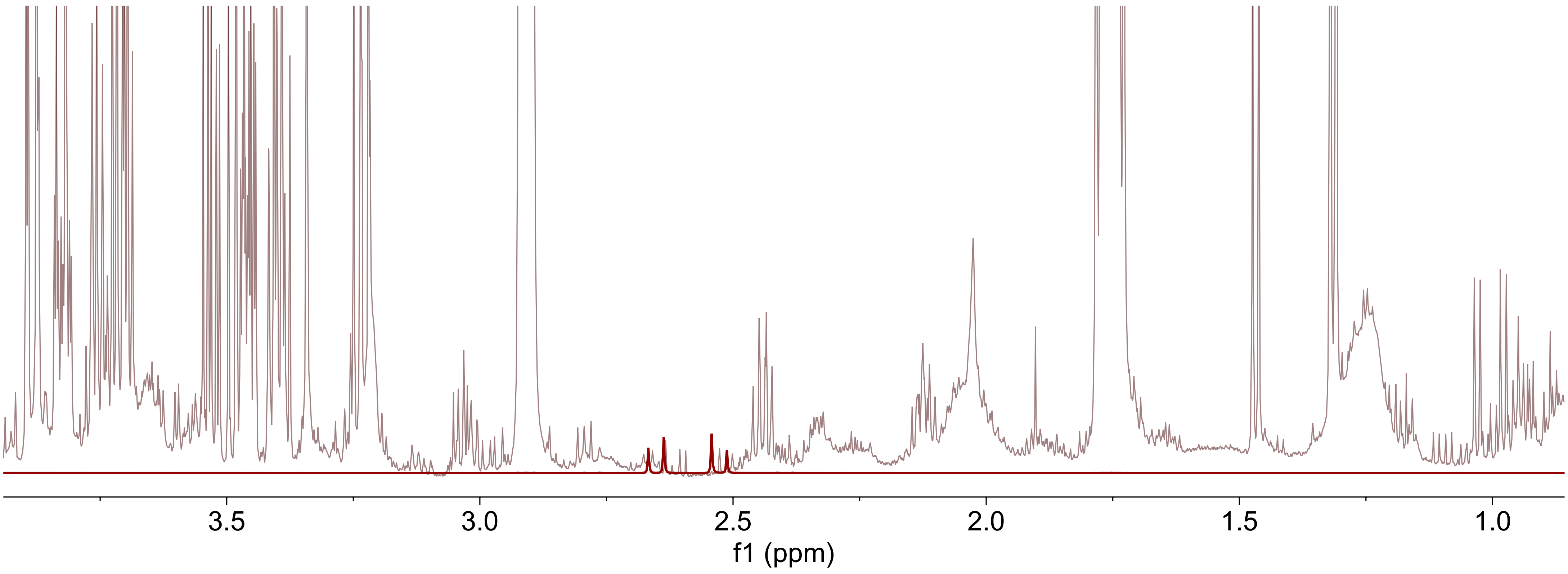

methionine

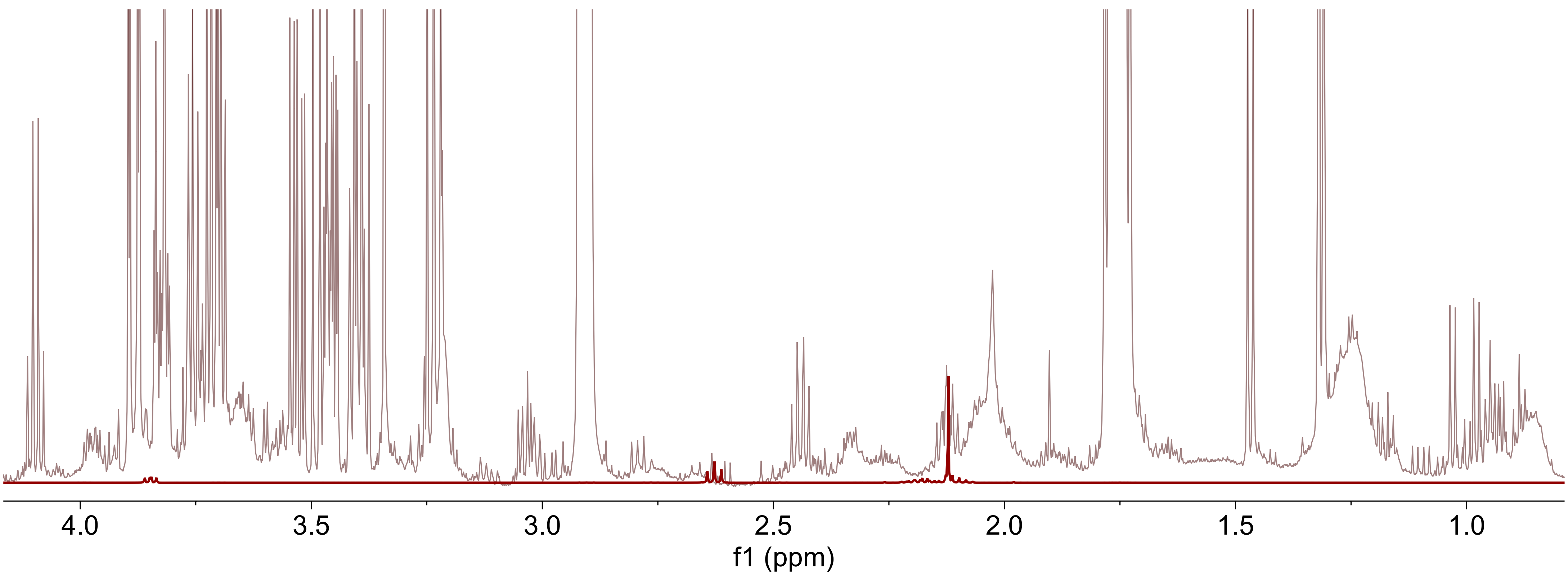

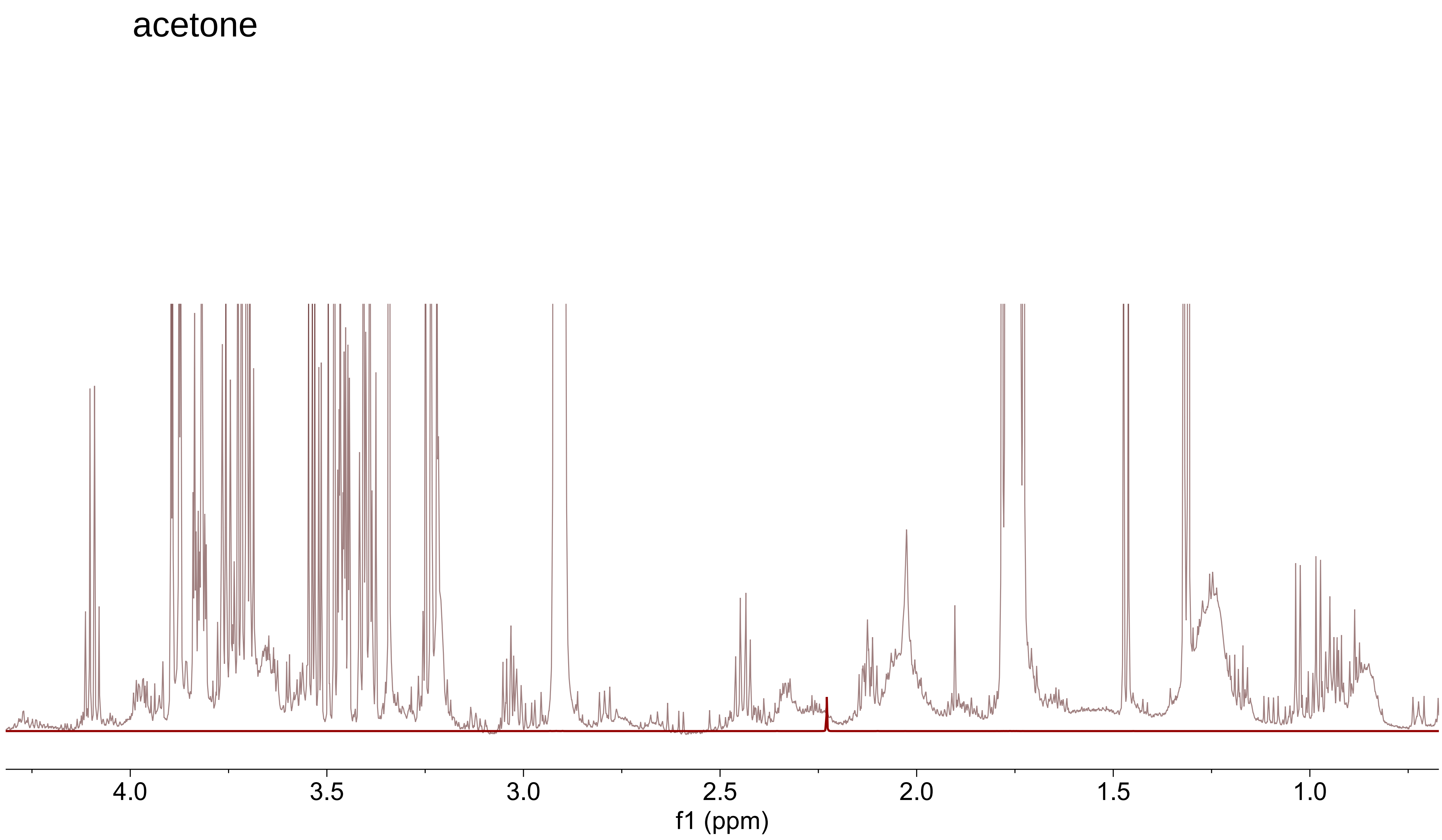

acetone
4.0
3.5
3.0
2.5
2.0
1.5
1.0
f1 (ppm)

aspartic acid

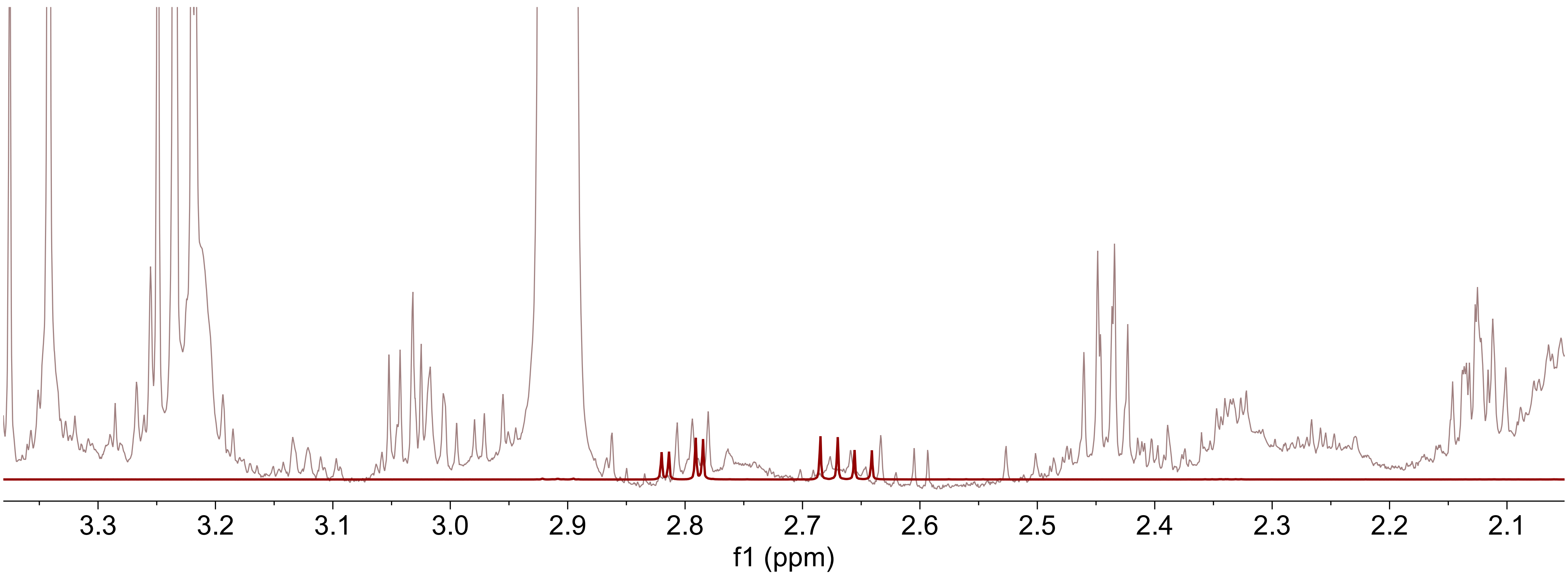

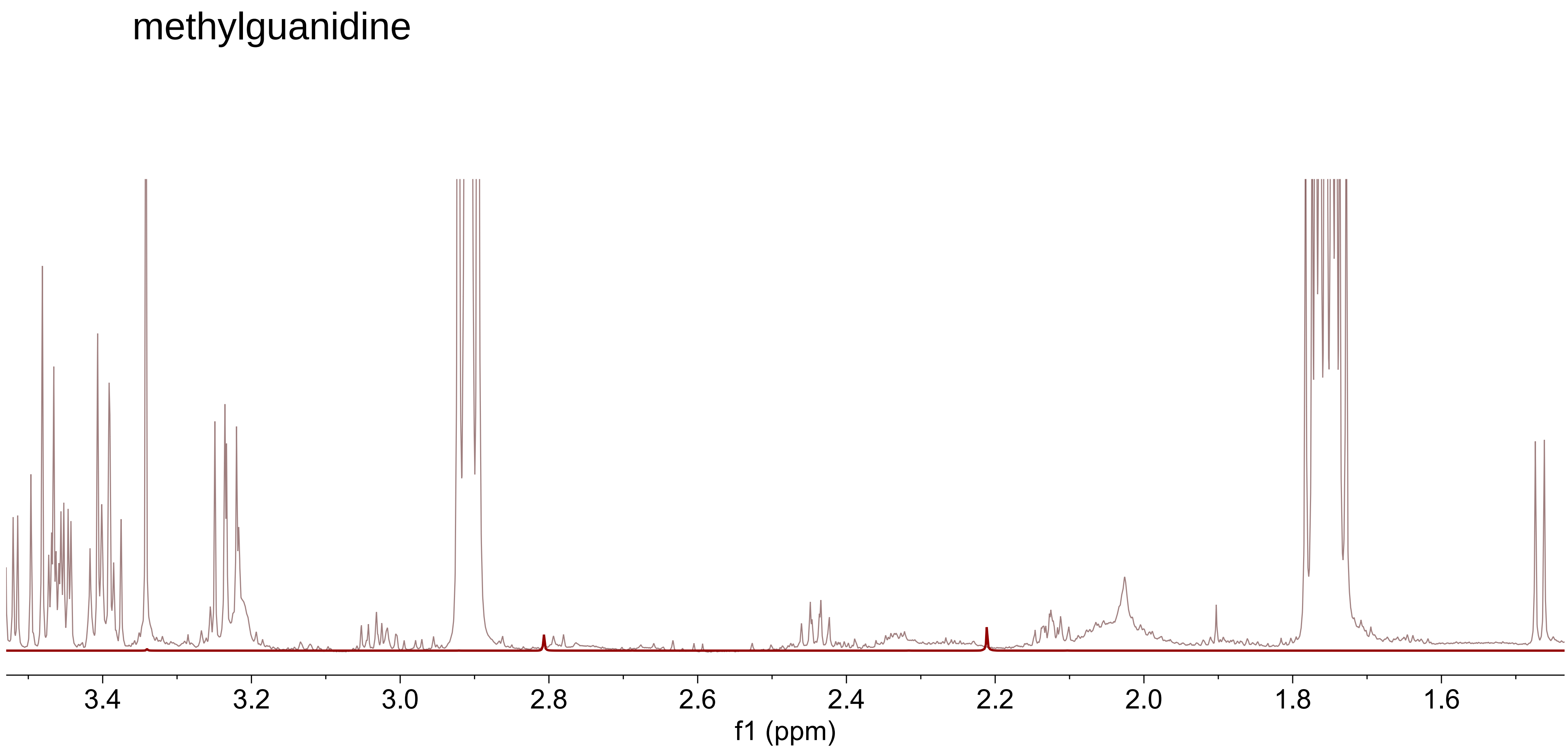
methylguanidine
3.4
3.2
3.0
2.8
2.6
2.4
2.2
2.0
1.8
1.6
f1 (ppm)

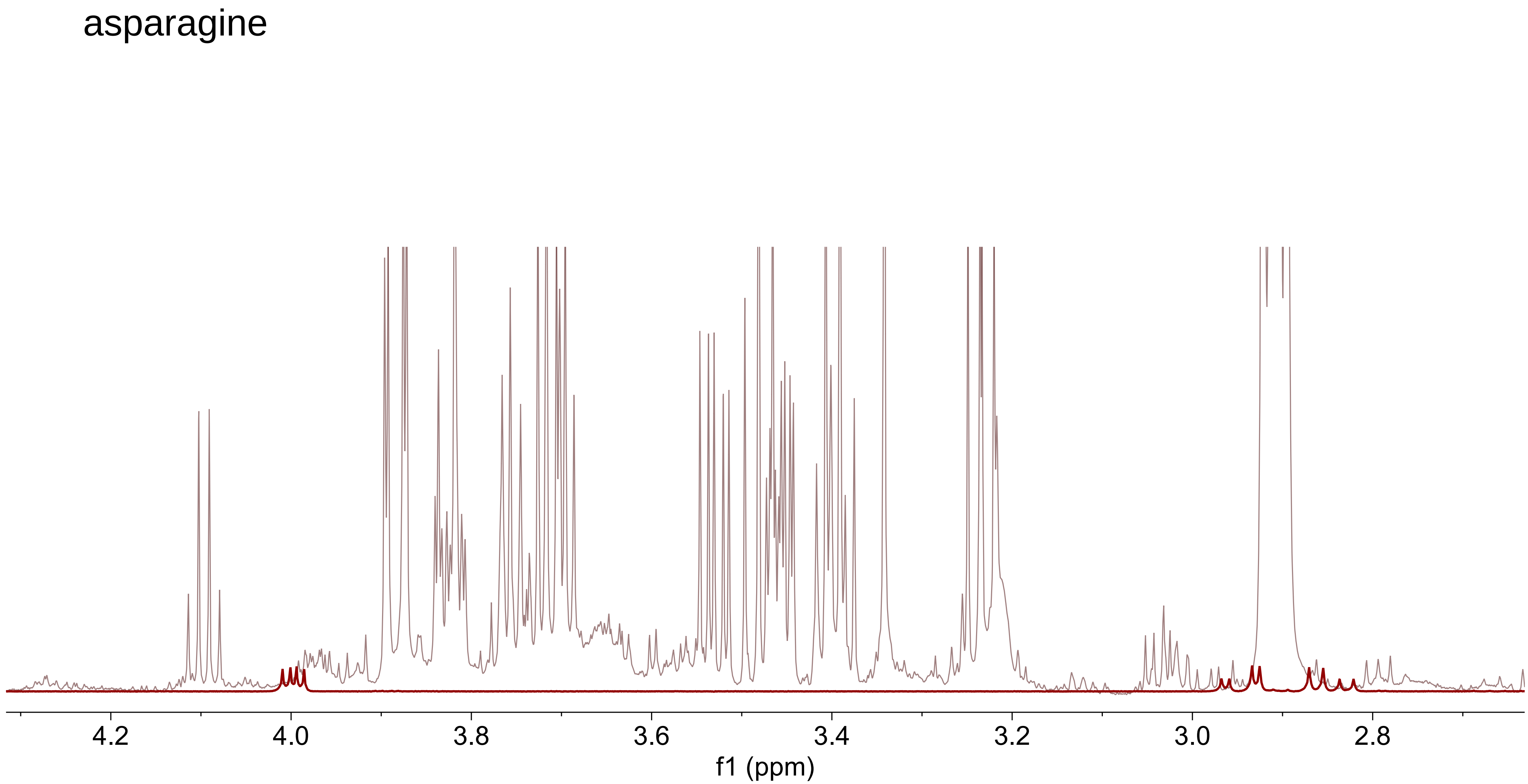
asparagine
4.2
4.0
3.8
3.6
3.4
3.2
3.0
2.8
f1 (ppm)

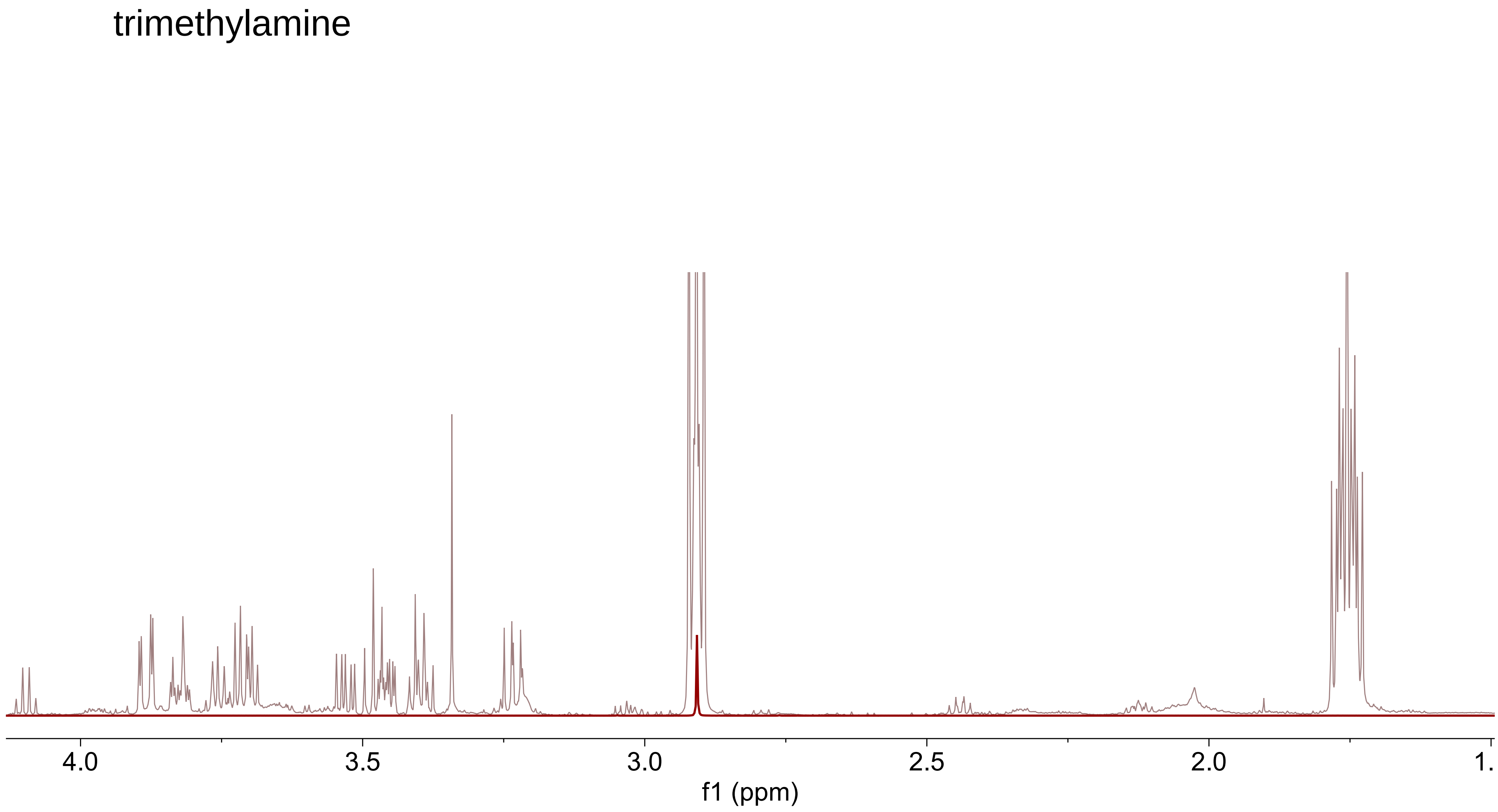
trimethylamine
4.0
3.5
3.0
2.5
2.0
1.
f1 (ppm)

sarcosine

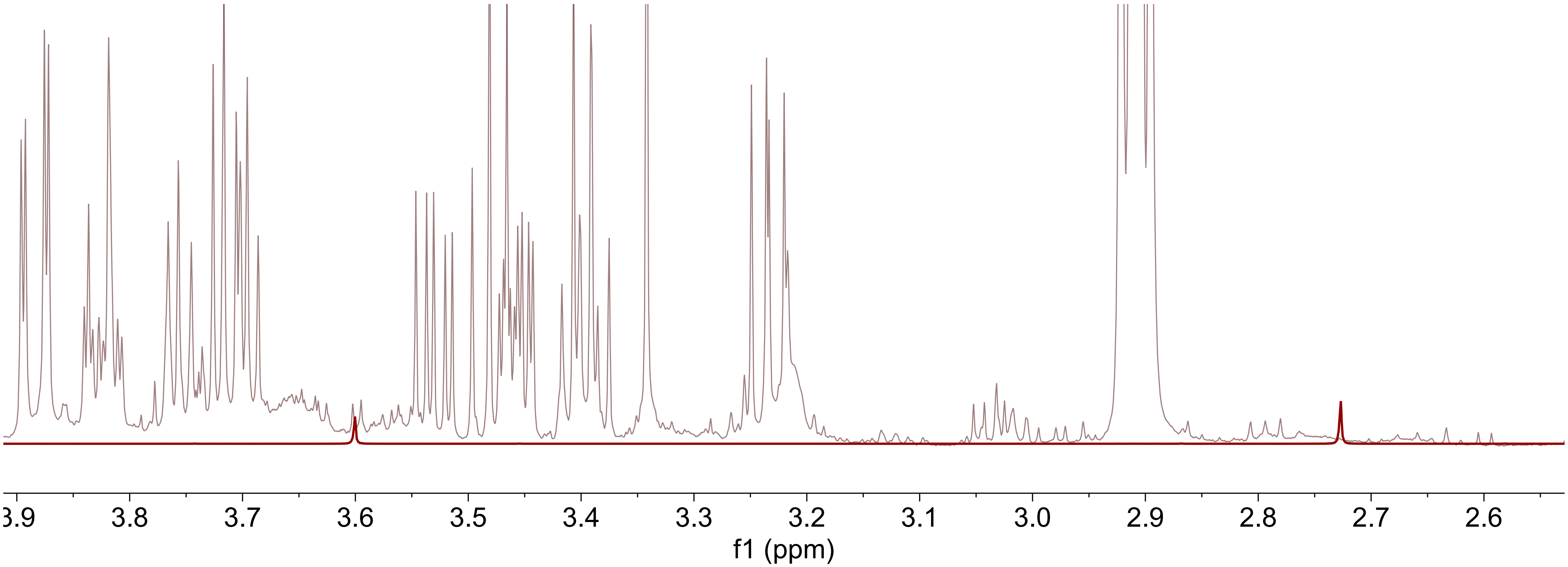

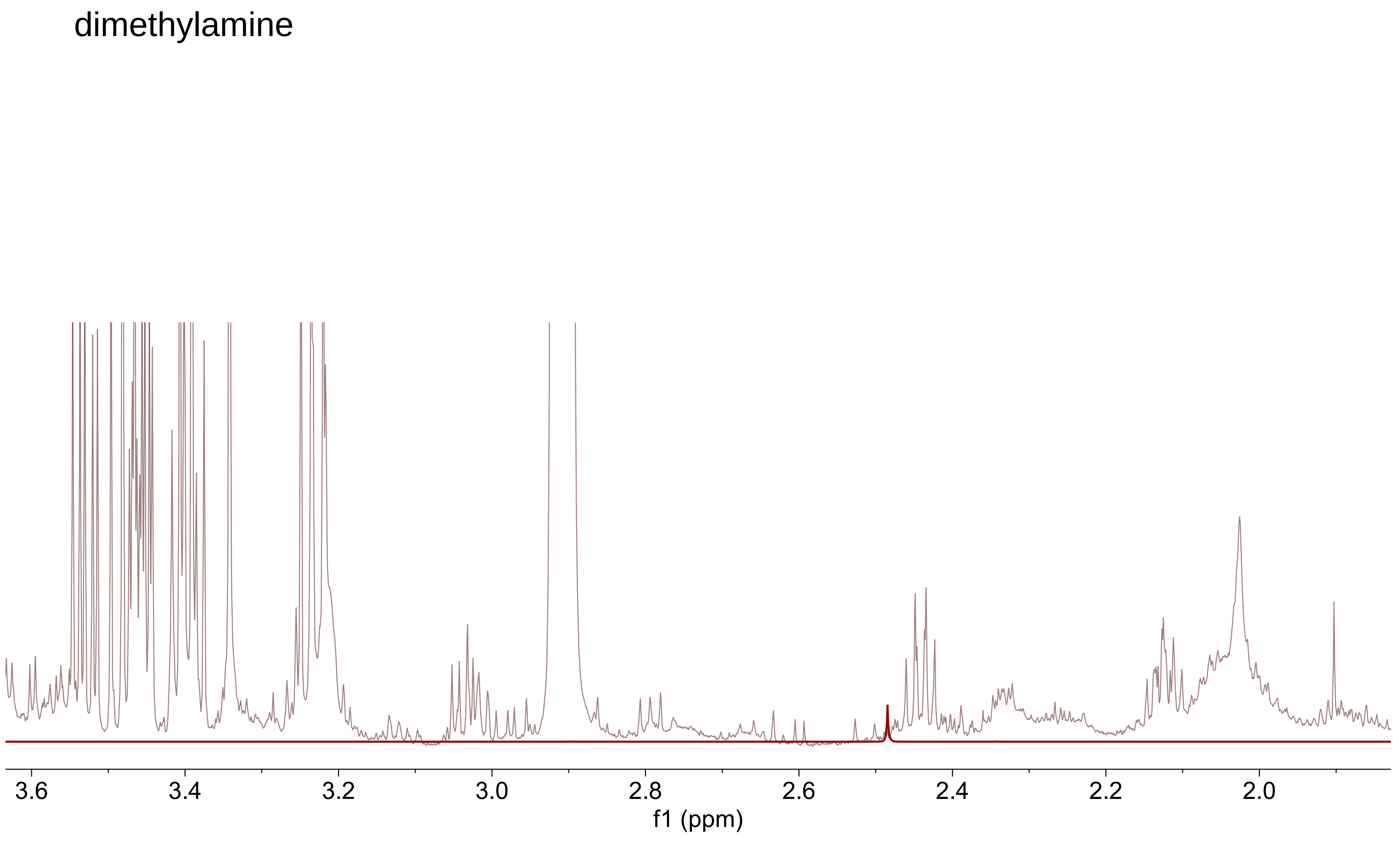
dimethylamine
3.6
3.4
3.2
3.0
2.8
2.6
2.4
2.2
2.0
f1 (ppm)

N,N-dimethylglycine

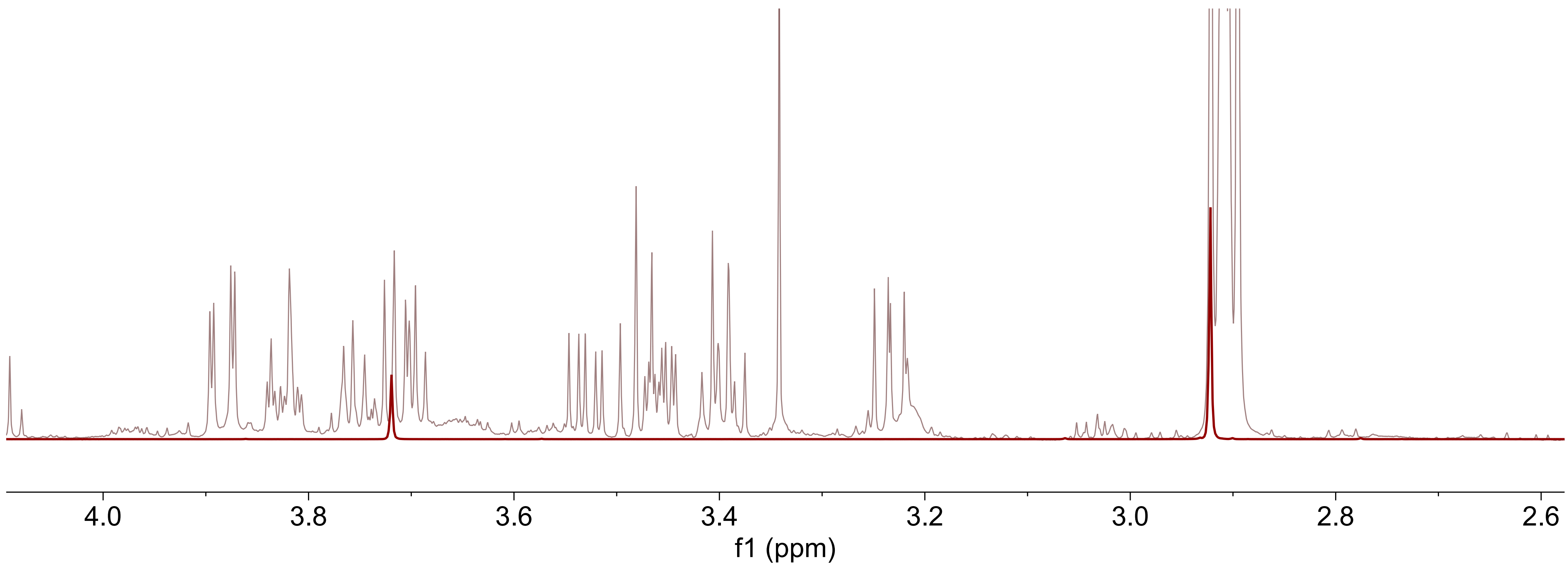

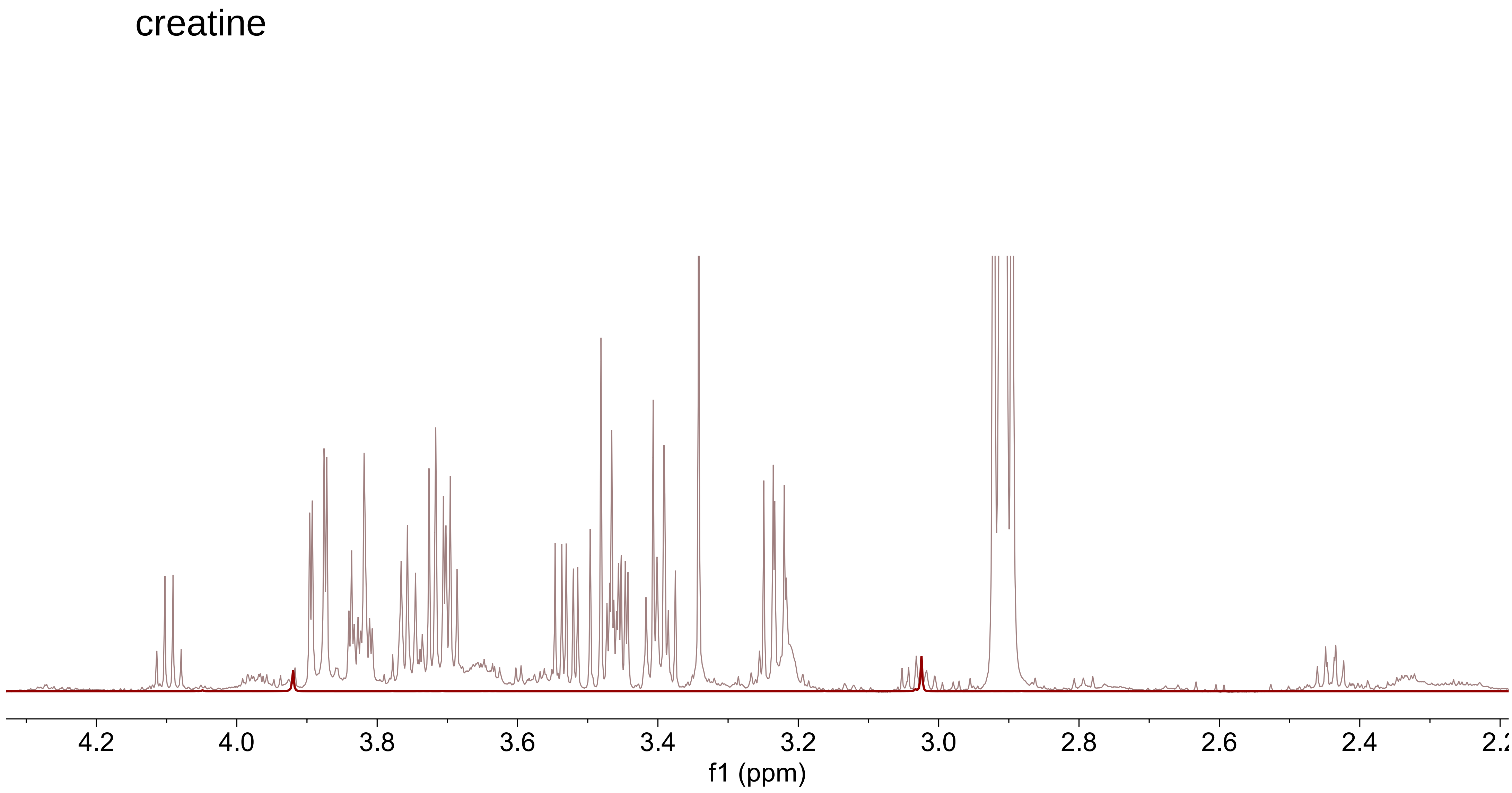
creatine
4.2
4.0
3.8
3.6
3.4
3.2
3.0
2.8
2.6
2.4
f1 (ppm)

dimethyl sulfone

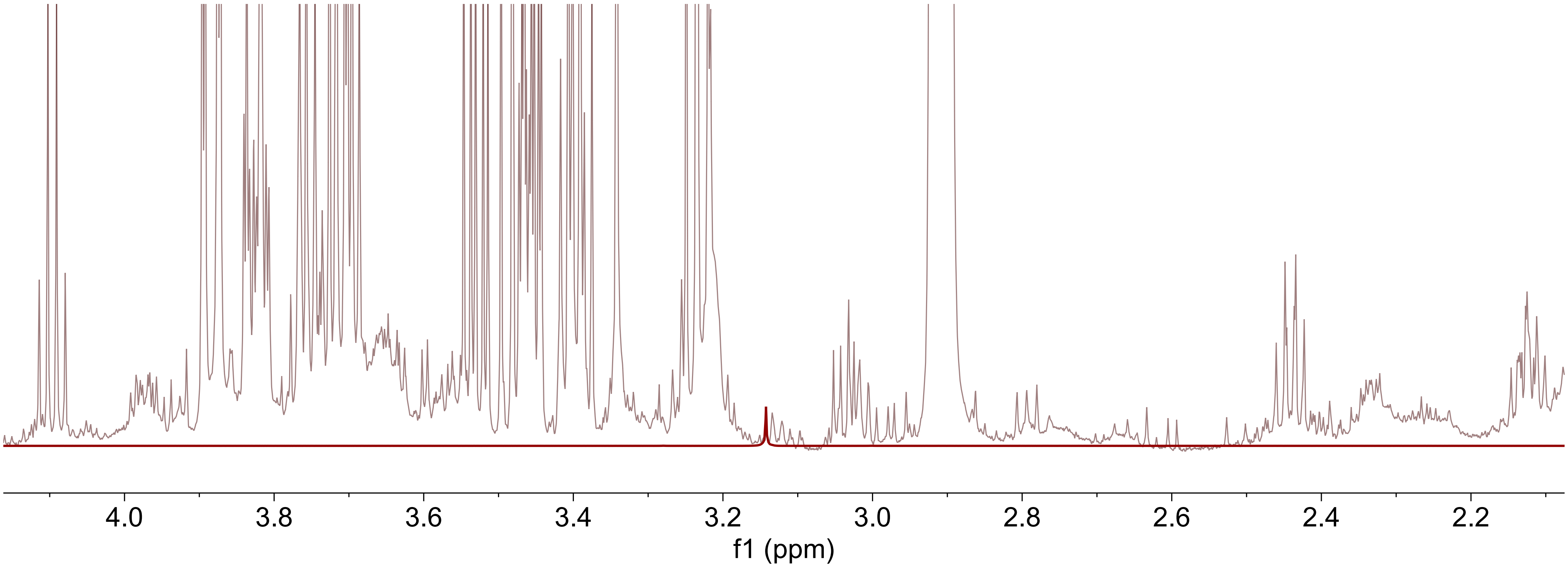

choline

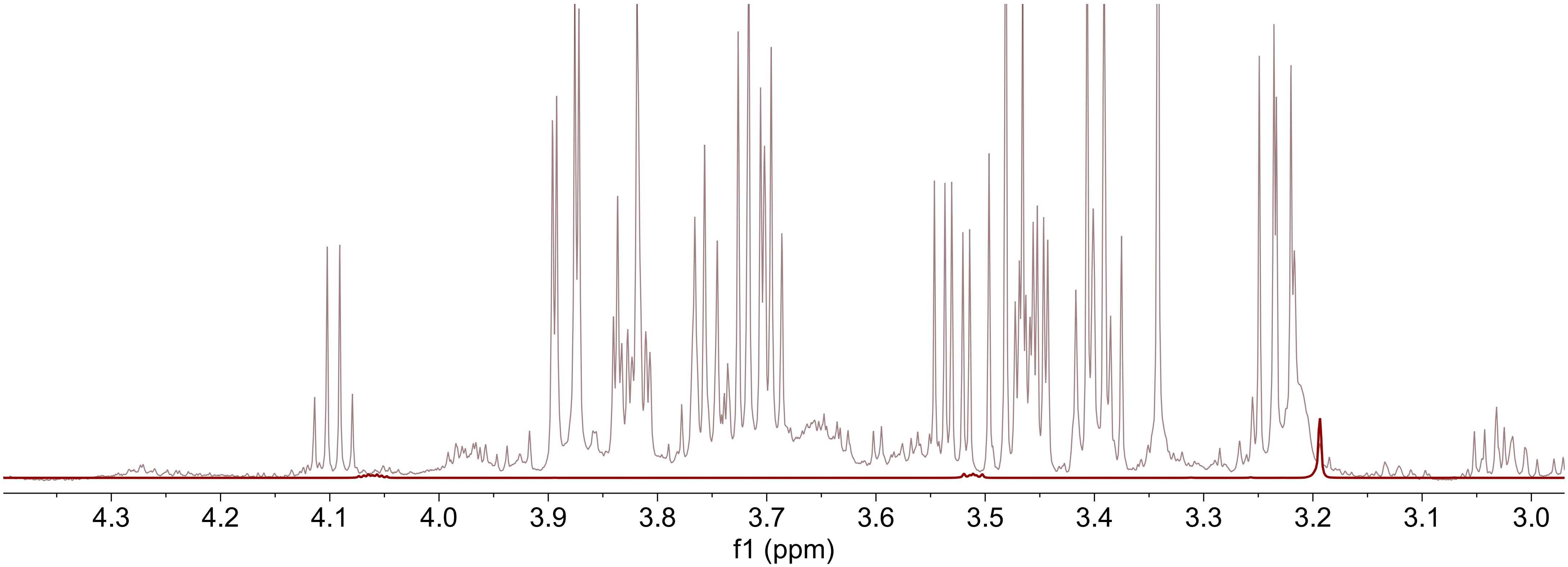

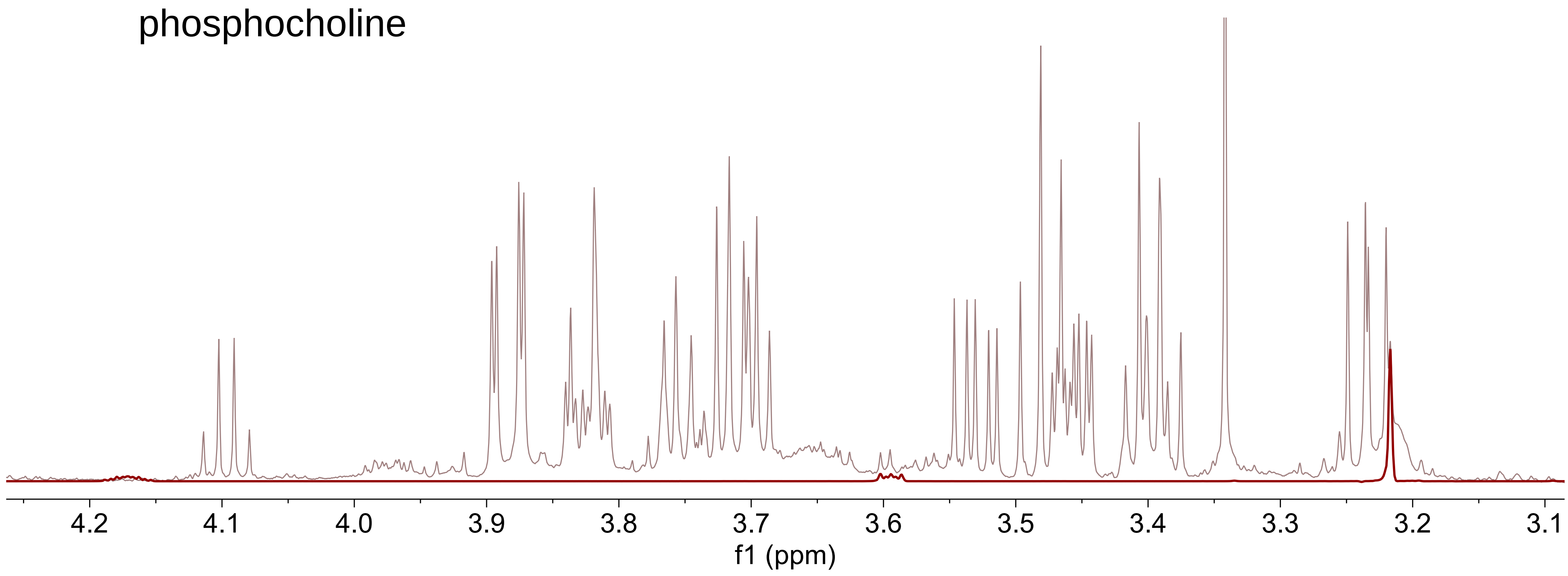
phosphocholine
4.2
4.1
4.0
3.9
3.8
3.7
3.6
3.5
3.4
3.3
3.2
3.1
f1 (ppm)

glycerophosphocholine

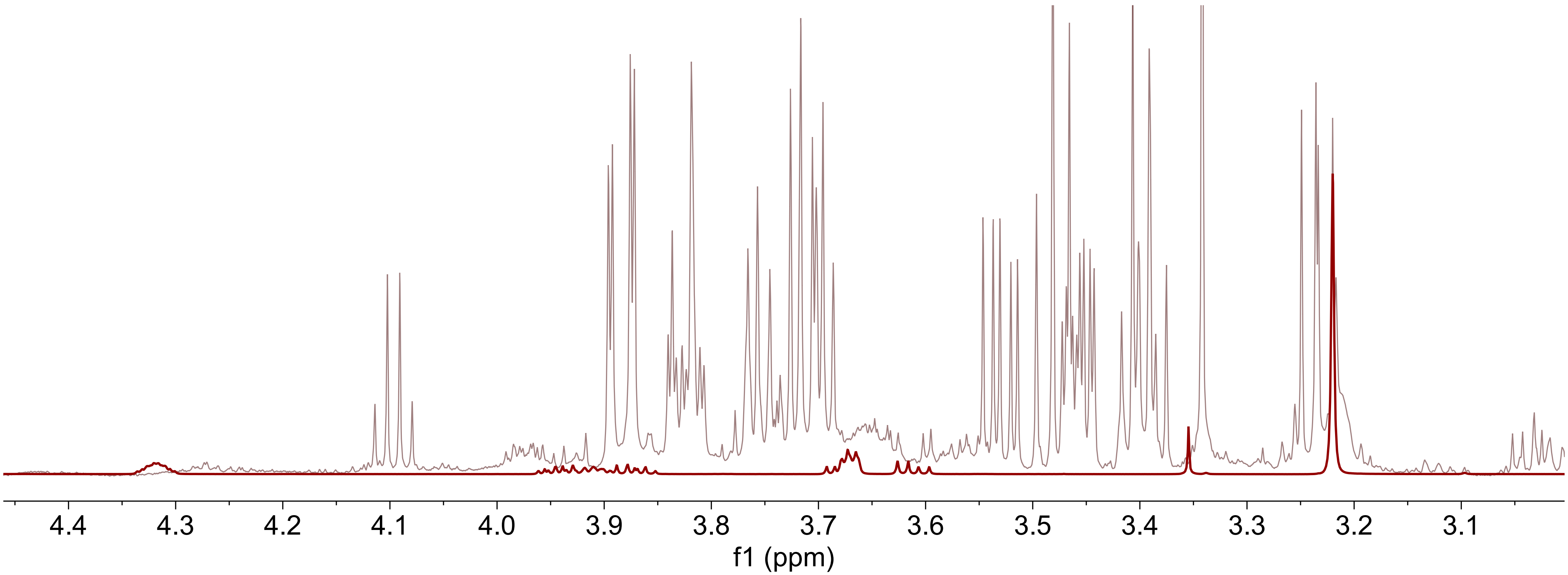

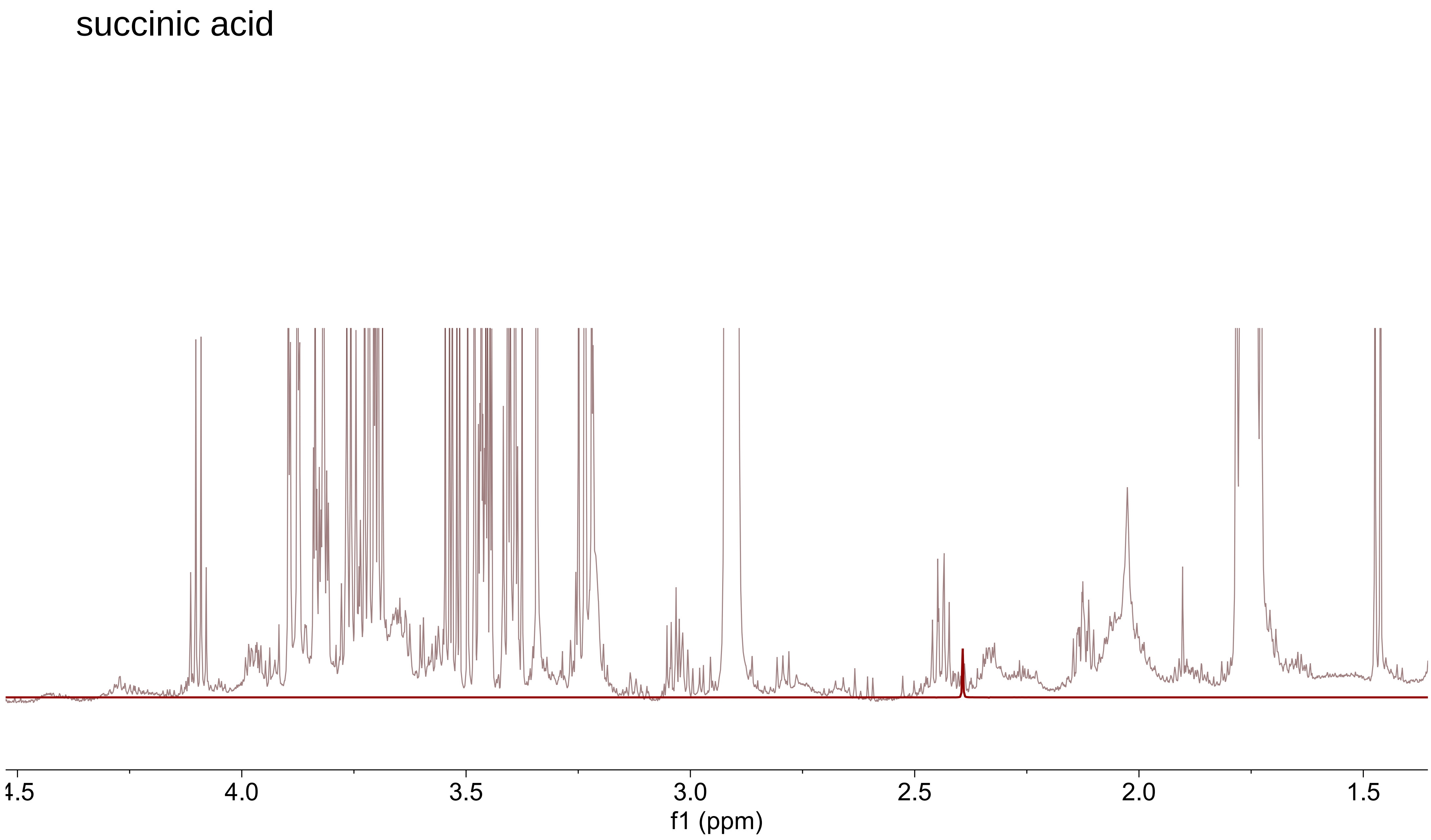

succinic acid
4.5
4.0
3.5
3.0
2.5
2.0
1.5
f1 (ppm)

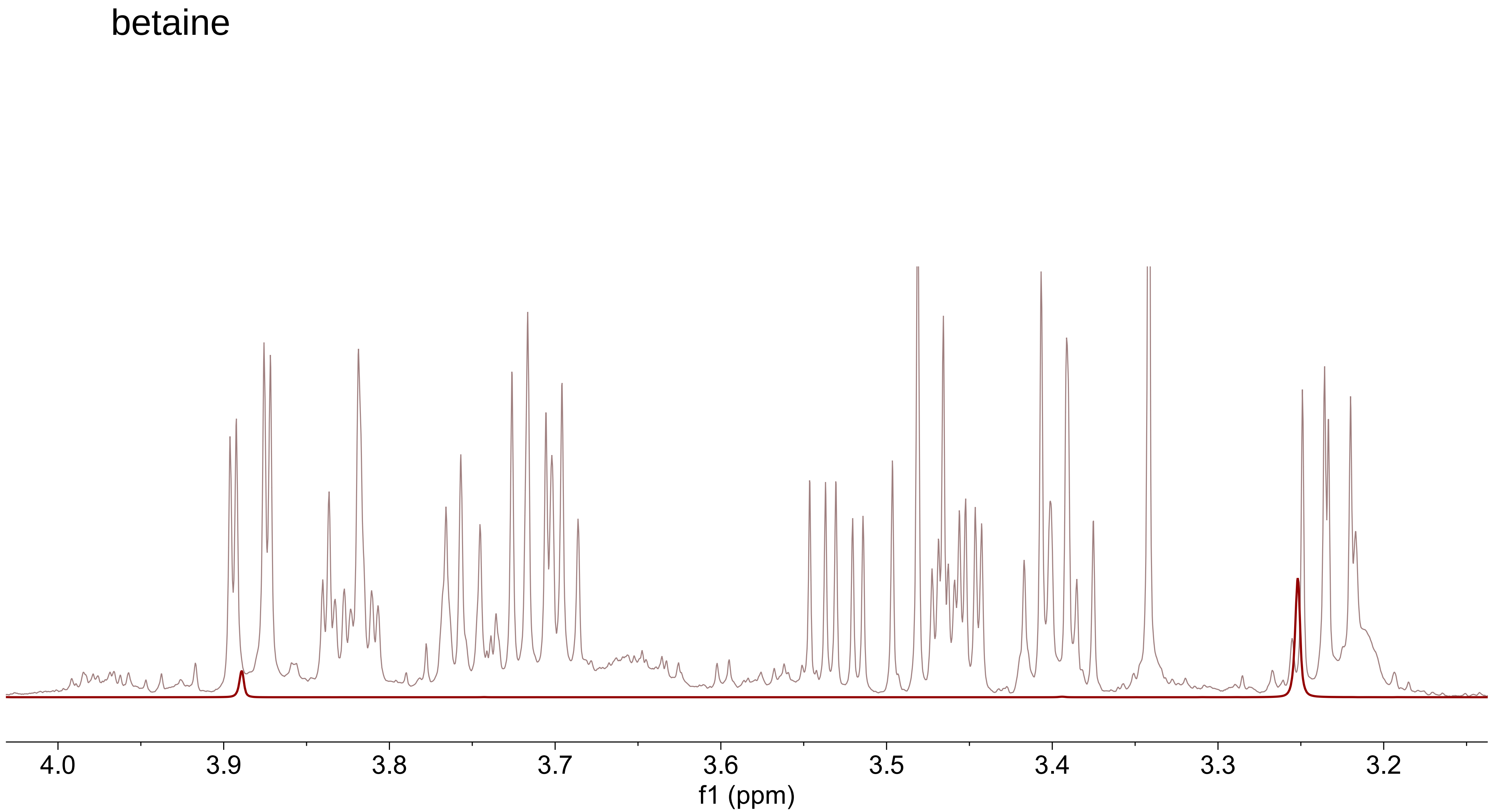
betaine
4.0
3.9
3.8
3.7
3.6
3.5
3.4
3.3
3.2
f1 (ppm)

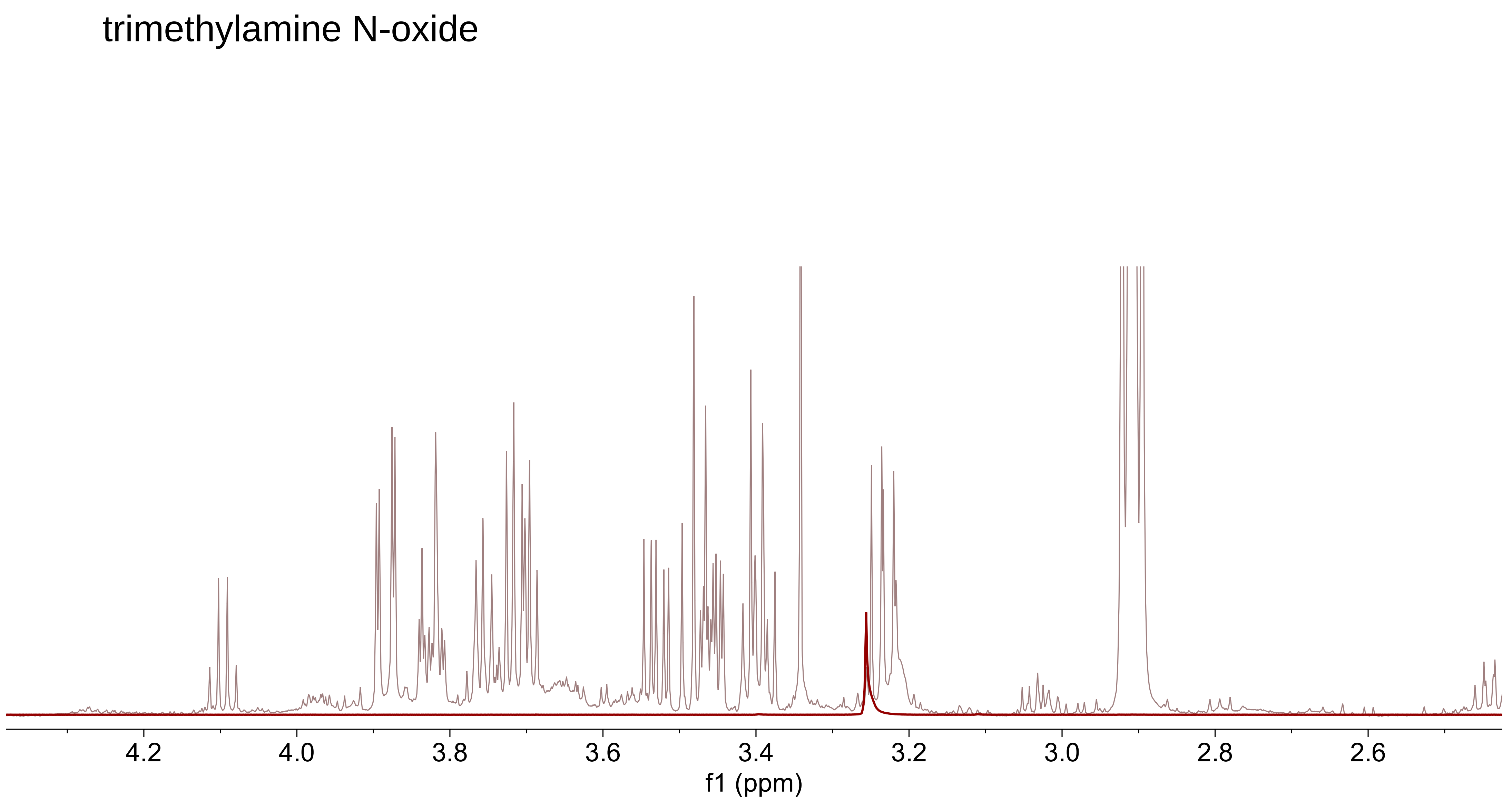
trimethylamine N-oxide
4.2
4.0
3.8
3.6
3.4
3.2
3.0
2.8
2.6
f1 (ppm)

myo-Inositol

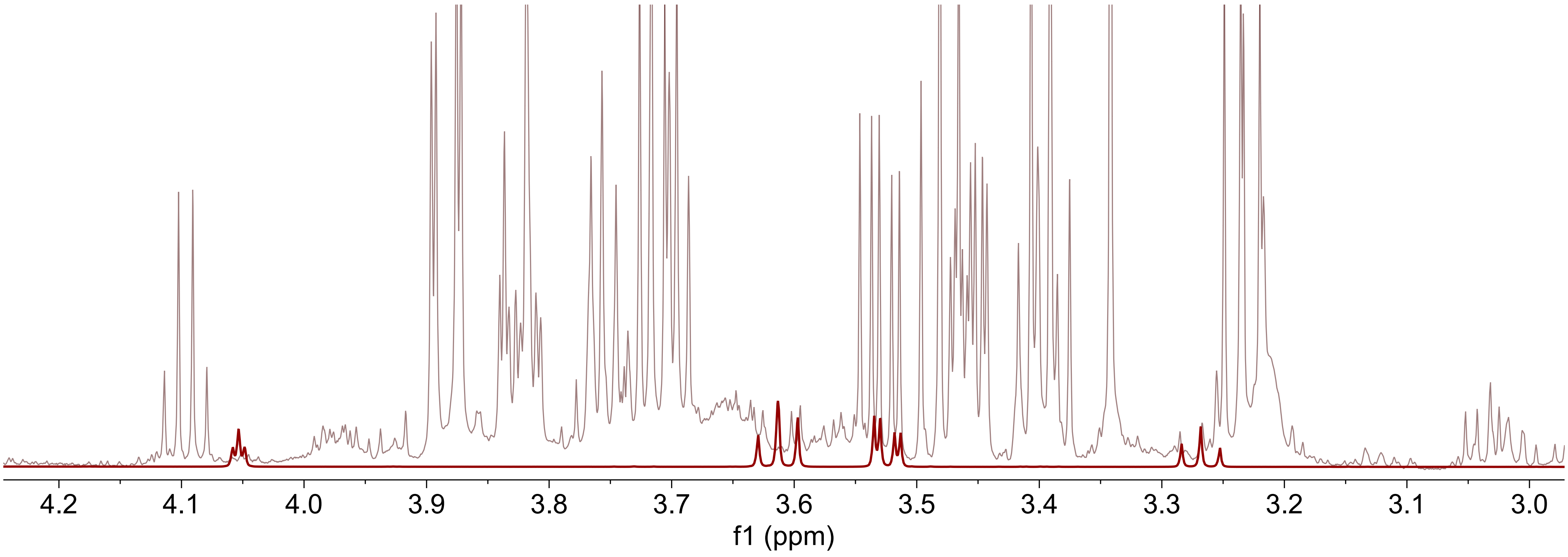

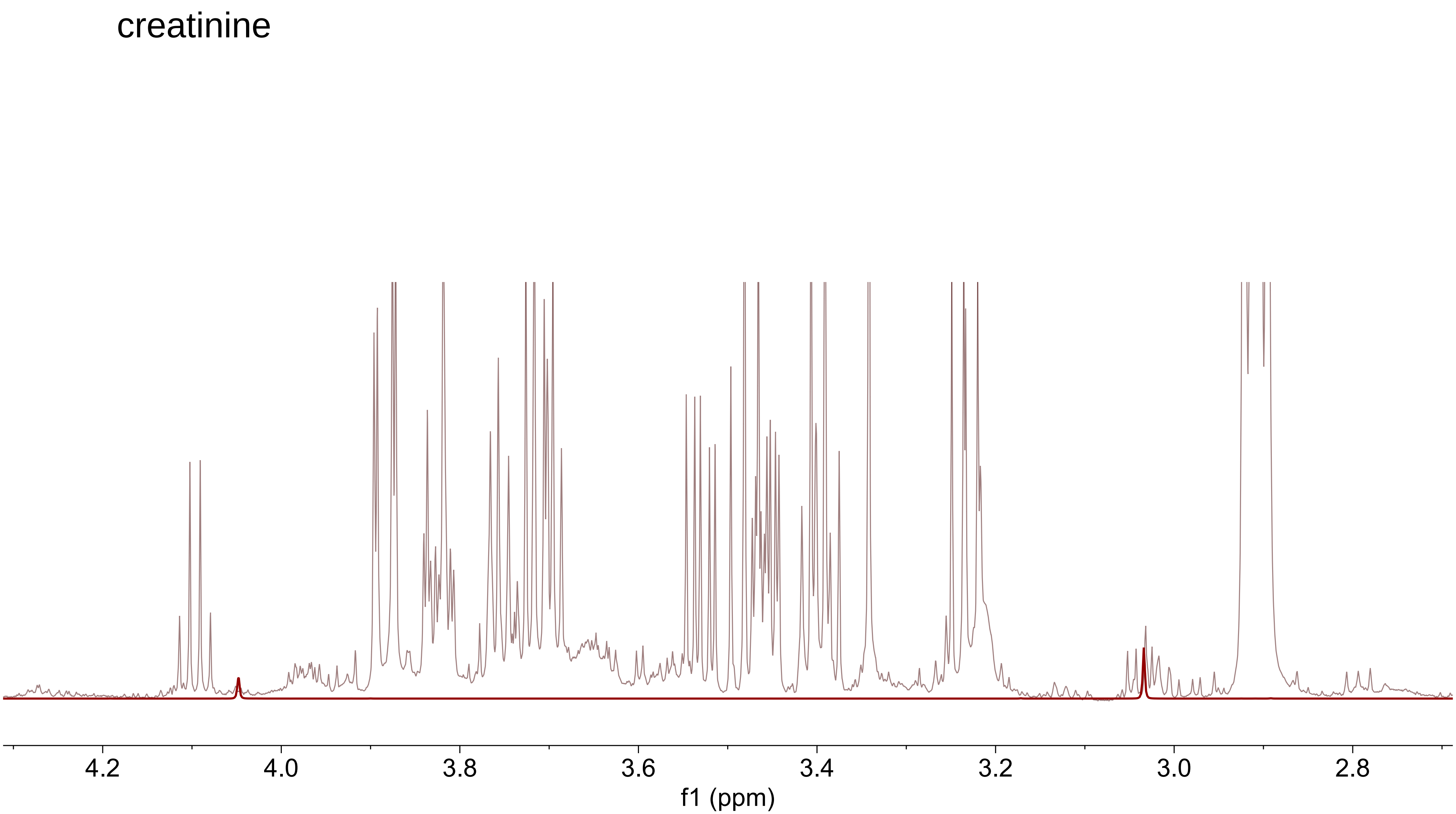
creatinine
4.2
4.0
3.8
3.6
3.4
3.2
3.0
2.8
f1 (ppm)

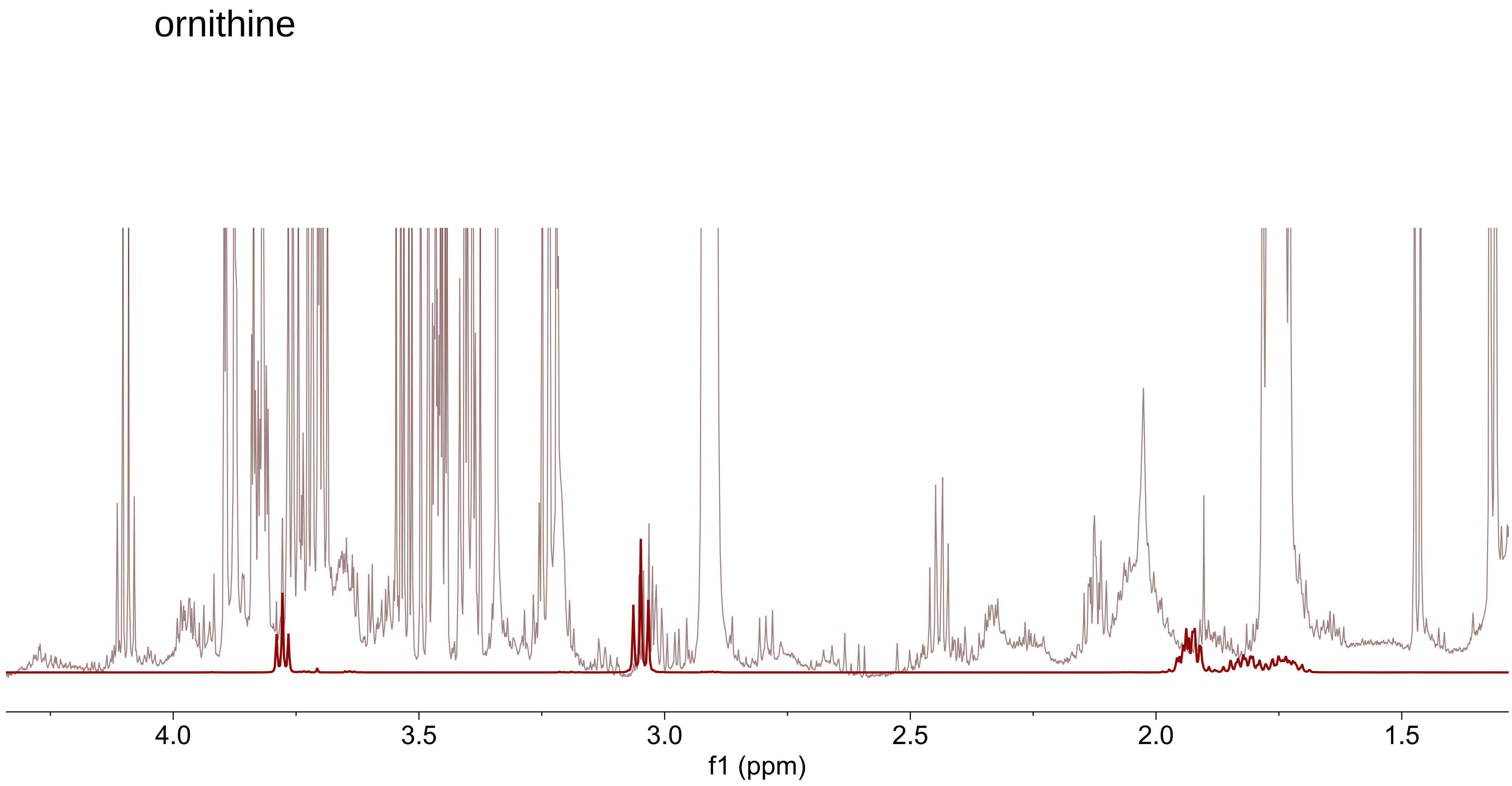
ornithine
4.0
3.5
3.0
2.5
2.0
1.5
f1 (ppm)

methanol

The methanol was introduced during the sample preparation process and was subtracted as the background peak

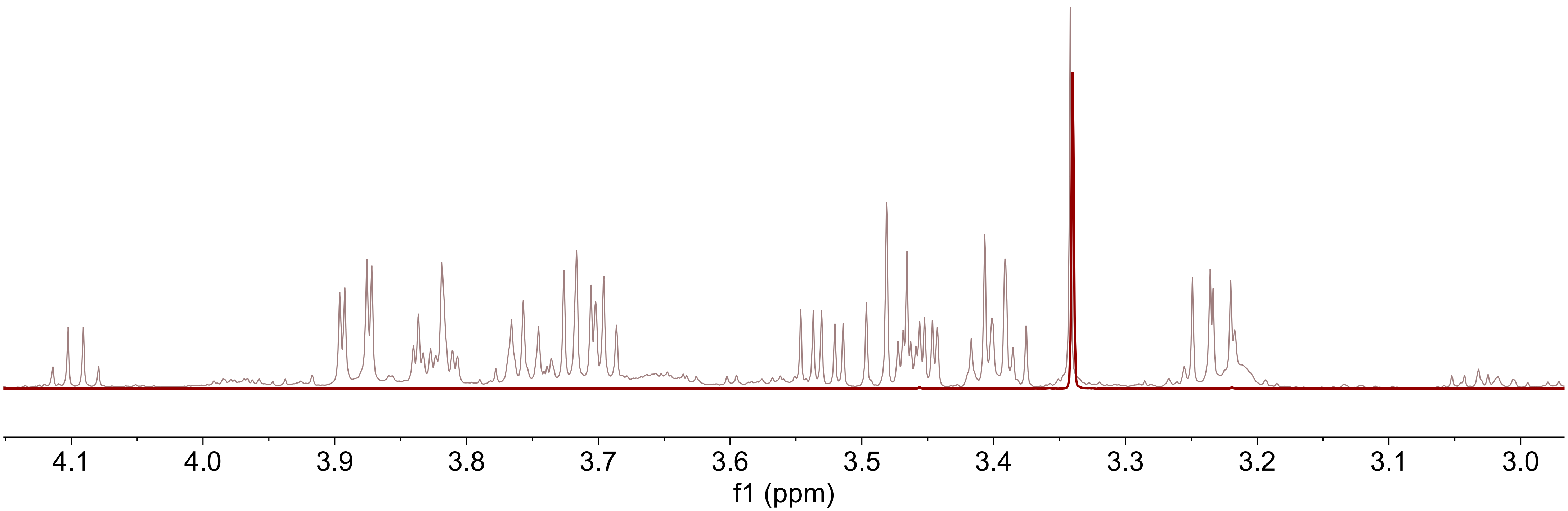

Figure S2

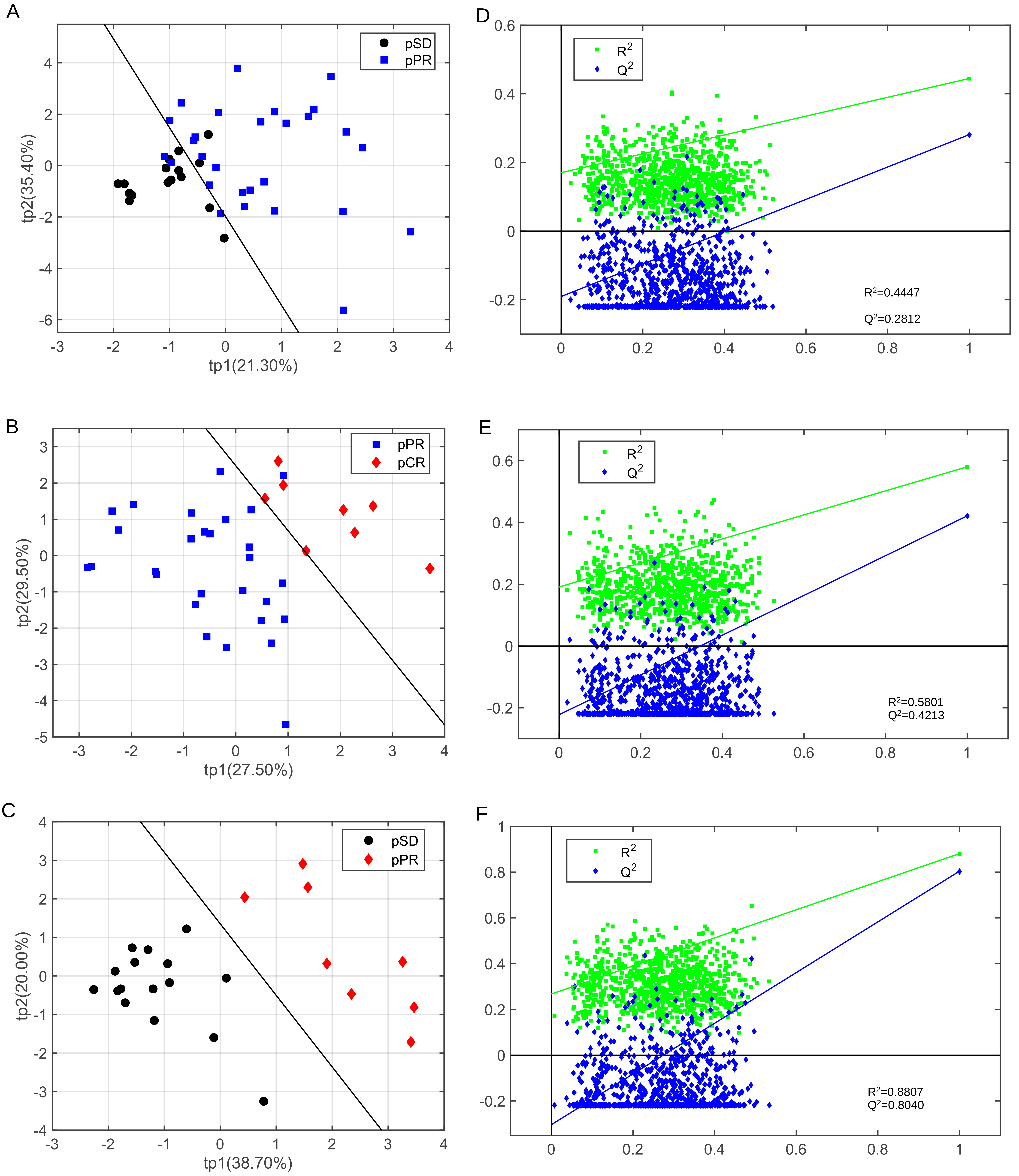
A
pSD
pPR
tp2(35.40%)
tp1(21.30%)
B
pPR
pCR
tp2(29.50%)
tp1(27.50%)
C
pSD
pPR
tp2(20.00%)
tp1(38.70%)
D
R2
Q2
R2=0.4447
Q2=0.2812
E
R2
Q2
R2=0.5801
Q2=0.4213
F
R2
Q2
R2=0.8807
Q2=0.8040

Figure S3

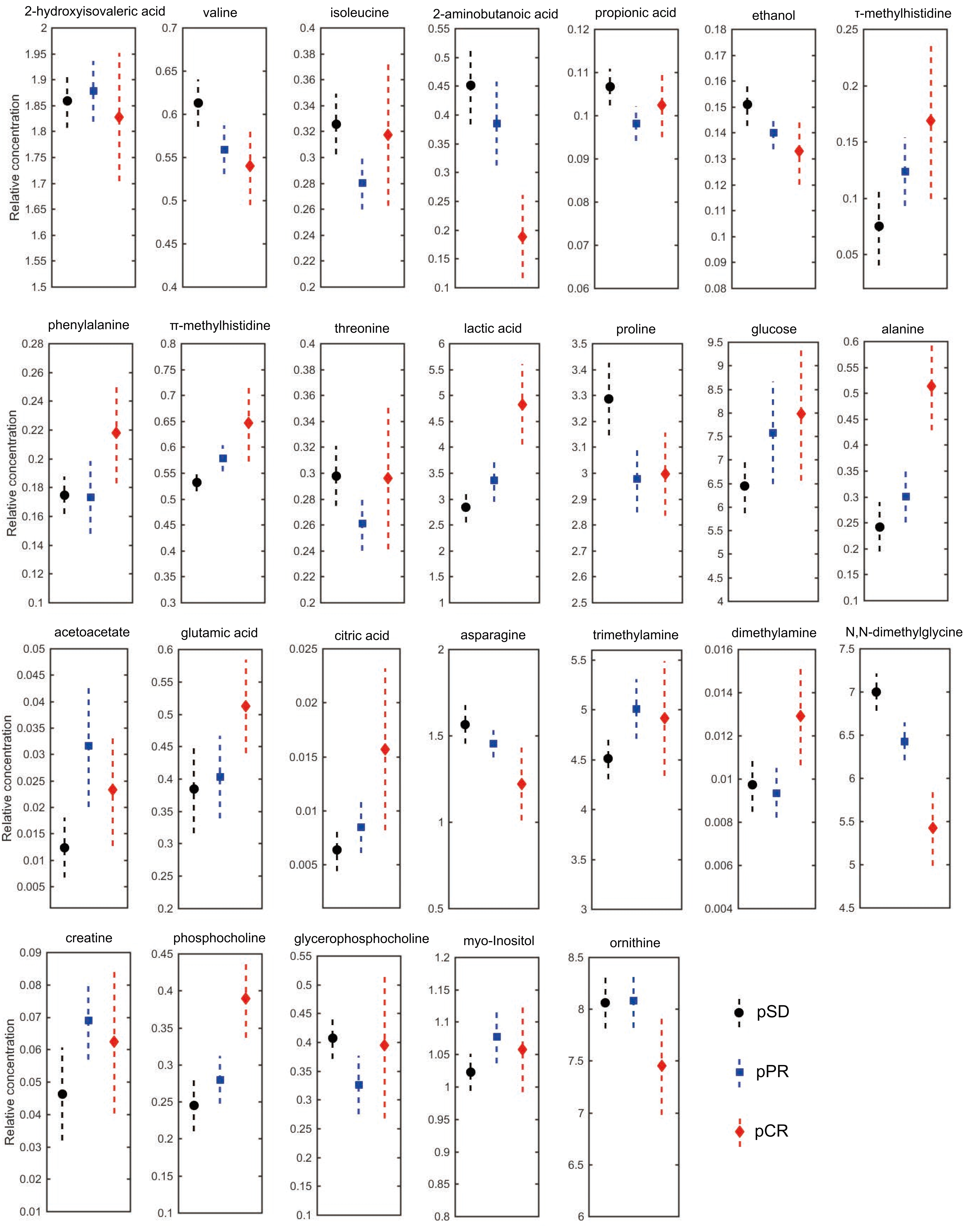
2-hydroxyisovaleric acid
valine
isoleucine
2-aminobutanoic acid
propionic acid
ethanol
τ-methylhistidine
phenylalanine
π-methylhistidine
threonine
lactic acid
proline
glucose
alanine
acetoacetate
glutamic acid
citric acid
asparagine
trimethylamine
dimethylamine
N,N-dimethylglycine
creatine
phosphocholine
glycerophosphocholine
myo-Inositol
ornithine
Relative concentration
pSD
pPR
pCR

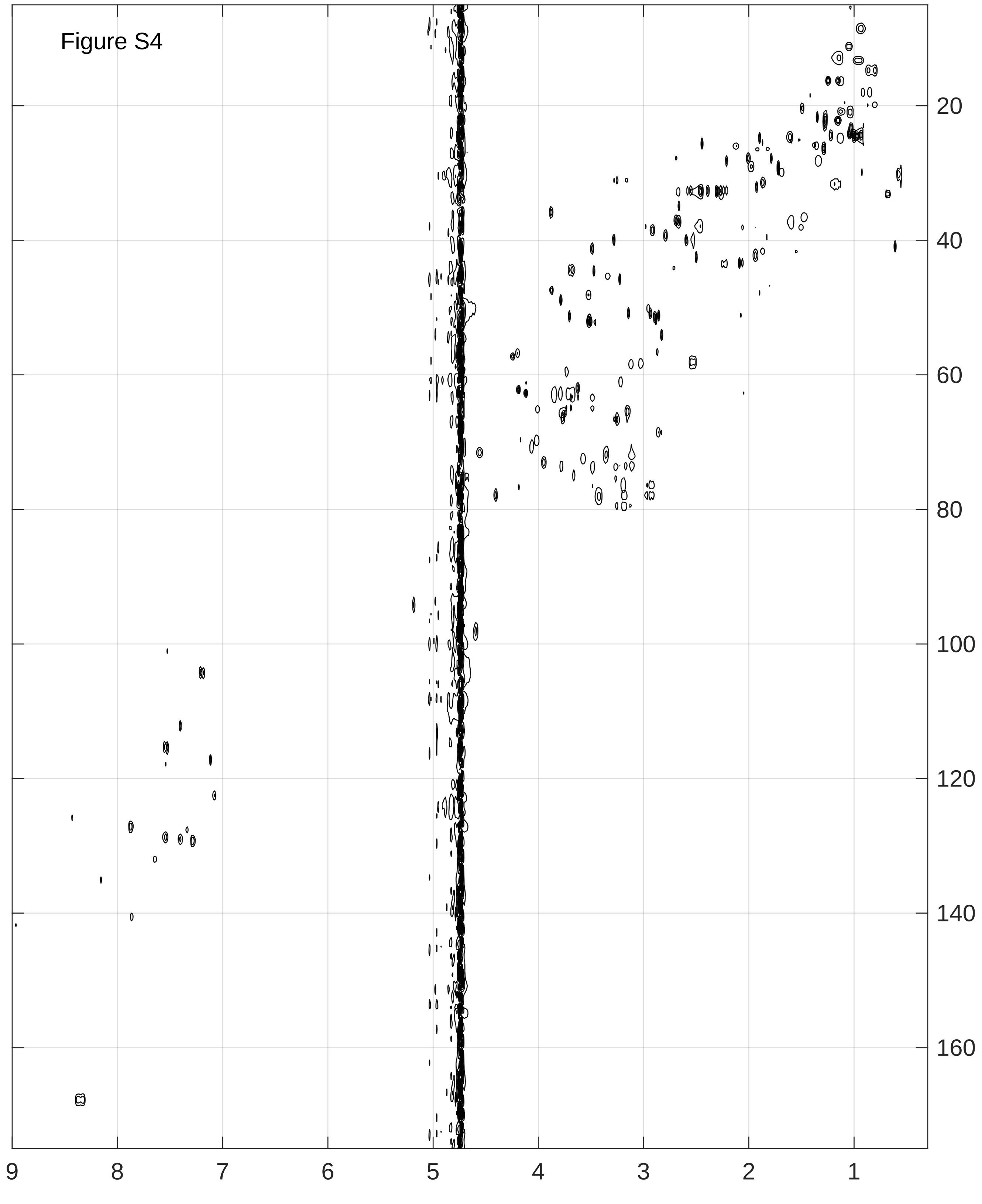
Figure S4
20
40
60
80
100
120
140
160
9
8
7
6
5
4
3
2
1
